# Venetoclax resistance in acute lymphoblastic leukemia is characterized by increased mitochondrial activity and can be overcome by co-targeting oxidative phosphorylation

**DOI:** 10.1038/s41419-024-06864-7

**Published:** 2024-07-03

**Authors:** Stefanie Enzenmüller, Alexandra Niedermayer, Felix Seyfried, Vera Muench, Daniel Tews, Ulrich Rupp, Eugen Tausch, Alexander Groß, Pamela Fischer-Posovszky, Paul Walther, Stephan Stilgenbauer, Hans A. Kestler, Klaus-Michael Debatin, Lüder Hinrich Meyer

**Affiliations:** 1 Department of Pediatrics and Adolescent Medicine, Ulm University Medical Center, Ulm, Germany; 2 International Graduate School in Molecular Medicine, Ulm University, Ulm, Germany; 3 Central Facility for Electron Microscopy, Ulm University, Ulm, Germany; 4 Division of Chronic Lymphocytic Leukemia, Department of Internal Medicine III, University of Ulm, Ulm, Germany; 5 Institute of Medical Systems Biology, Ulm University, Ulm, Germany

**Keywords:** Experimental models of disease, Acute lymphocytic leukaemia

## Abstract

Deregulated apoptosis signaling is characteristic for many cancers and contributes to leukemogenesis and treatment failure in B-cell precursor acute lymphoblastic leukemia (BCP-ALL). Apoptosis is controlled by different pro- and anti-apoptotic molecules. Inhibition of anti-apoptotic molecules like B-cell lymphoma 2 (BCL-2) has been developed as therapeutic strategy. Venetoclax (VEN), a selective BCL-2 inhibitor has shown clinical activity in different lymphoid malignancies and is currently evaluated in first clinical trials in BCP-ALL. However, insensitivity to VEN has been described constituting a major clinical concern. Here, we addressed and modeled VEN-resistance in BCP-ALL, investigated the underlying mechanisms in cell lines and patient-derived xenograft (PDX) samples and identified potential strategies to overcome VEN-insensitivity. Leukemia lines with VEN-specific resistance were generated in vitro and further characterized using RNA-seq analysis. Interestingly, gene sets annotated to the citric/tricarboxylic acid cycle and the respiratory electron transport chain were significantly enriched and upregulated, indicating increased mitochondrial metabolism in VEN-resistant ALL. Metabolic profiling showed sustained high mitochondrial metabolism in VEN-resistant lines as compared to control lines. Accordingly, primary PDX-ALL samples with intrinsic VEN-insensitivity showed higher oxygen consumption and ATP production rates, further highlighting that increased mitochondrial activity is a characteristic feature of VEN-resistant ALL. VEN-resistant PDX-ALL showed significant higher mitochondrial DNA content and differed in mitochondria morphology with significantly larger and elongated structures, further corroborating our finding of augmented mitochondrial metabolism upon VEN-resistance. Using Oligomycin, an inhibitor of the complex V/ATPase subunit, we found synergistic activity and apoptosis induction in VEN-resistant BCP-ALL cell lines and PDX samples, demonstrating that acquired and intrinsic VEN-insensitivity can be overcome by co-targeting BCL-2 and the OxPhos pathway. These findings of reprogrammed, high mitochondrial metabolism in VEN-resistance and synergistic activity upon co-targeting BCL-2 and oxidative phosphorylation strongly suggest further preclinical and potential clinical evaluation in VEN-resistant BCP-ALL.

## Introduction

Dysregulated cell death signaling is a hallmark of many cancers, including B-cell precursor acute lymphoblastic leukemia (BCP-ALL), and contributes to leukemogenesis and treatment failure. Apoptosis signaling is characterized by an interplay of different pro- and anti-apoptotic proteins controlling subsequent steps like caspase activation, chromatin condensation and DNA cleavage [1]. B-cell lymphoma 2 (BCL-2) family proteins are critical regulators of these processes. Anti-apoptotic members like BCL-2, BCL-XL or MCL-1 contain three or four BCL-2 Homology (BH) domains, while pro-apoptotic molecules comprise BH3-only proteins (BAD, BID, BIM, NOXA, PUMA) or proteins with two or three BH-domains (BAX, BAK) [2]. Anti-apoptotic proteins counteract apoptosis induction by directly binding to pro-apoptotic BH3-only proteins or preventing BAX and BAK activation [3]. BIM and BID, two major apoptosis activating proteins, directly interact with BAX and BAK, facilitating their oligomerization and subsequent integration into the mitochondrial outer membrane. This process triggers the initiation of Mitochondrial Outer Membrane Permeabilization (MOMP), release of apoptogenic factors like cytochrome c and caspase activation, ultimately resulting in cell death [4]. One possible strategy to evade apoptosis is to upregulate anti-apoptotic proteins. For example, genetic alterations such as the translocation t(14;18)(q32;q21) leading to the loss of negative regulatory miRNA-15a/16-1, or amplification of the chromosomal region 18q21, as frequently observed in mantle cell and diffuse large B cell lymphoma, result in upregulated BCL-2 [5, 6]. Therefore, BCL-2 represents an attractive target. Selective BCL-2 inhibition by Venetoclax (VEN) is a successful therapeutic strategy in various lymphoid malignancies, including preclinical activity in BCP-ALL [7–12]. However, despite its clinical effectivity, intrinsic insensitivity or secondary acquired resistance to VEN have been described, leading to disease progression and treatment failure [13, 14].

In this study, we addressed and modeled VEN-resistance in BCP-ALL and investigated underlying mechanisms using cell lines and patient-derived xenograft (PDX) samples to identify potential strategies to overcome VEN-insensitivity. VEN-resistant cells were characterized by increased and sustained mitochondrial activity, also upon re-exposure to VEN. In line, VEN-resistant leukemia was characterized by larger and elongated mitochondria, lower expression levels of the mitochondrial fission factor DRP1 and significantly higher mitochondrial DNA content, further corroborating our finding of augmented mitochondrial metabolism in VEN-resistant ALL. Using Oligomycin, an inhibitor of the complex V/ATPase subunit in combination with VEN resulted in synergistic induction of apoptosis in BCP-ALL cell lines and PDX samples. This highlights the potential to overcome both acquired and intrinsic VEN-insensitivity by co-targeting BCL-2 and the oxidative phosphorylation (OxPhos) pathway, thus providing the basis for further preclinical and clinical evaluation.

## Materials and Methods

Detailed information is additionally provided in the data supplement.

### BCP-ALL cell lines

RS4;11, MHH-CALL-2, KOPN-8, EU-3, RCH-ACV and NALM-6 cells were purchased (DSMZ, Braunschweig Germany) and cultured in RPMI-1640 medium (20% fetal bovine serum, 1% L-Glutamine, 1% Penicillin/Streptomycin; 5% CO_2_, 37 °C).

### Modeling of VEN-resistance in RS4;11 cell line

Starting from the BCP-ALL cell line RS4;11, five VEN-resistant lines were generated in parallel by exposure to increasing concentrations of VEN (Selleckchem, Houston Texas, USA) or solvent (DMSO, Serva, Heidelberg, Germany). Half maximal effective concentrations (EC_50_) were determined analyzing cell death by flow cytometry according to FSC/SSC criteria (Attune NxT Flow Cytometer, ThermoFisher, Waltham, Massachusetts, USA). VEN^ins^ lines were kept in culture with continuous exposure to VEN, while VEN^sens^ and drug holiday lines were kept in medium without VEN. Before subjecting the cells to the respective analysis, VEN was removed by washing.

### BCP-ALL Patient-Derived Xenograft (PDX) samples

Primary leukemia samples of BCP-ALL patients were collected after written informed consent in accordance with the institution’s ethical review board (Ethics Commission, Ulm University, #461/19). Patient-derived xenograft samples were generated by intravenous transplantation of ALL cells into female NOD/SCID mice (NOD.CB17-Prkdcscid, Charles River, Wilmington, Massachusetts, USA) as described before [15]. Animal experiments were approved (Regierungspräsidium Tübingen, Tierversuchsanzeige Nr. V.48).

### Targeted sequencing of BCL-2 family members

DNA of RS4;11 VEN^sens^ and VEN^ins^ samples was isolated (QIAamp DNA Blood Mini Kit, Qiagen, Hilden, Germany) according to the manufacturer´s protocol.

Gene mutation analysis of *BCL2*, *MCL1*, *BCL2L1* (BCL-XL), *BCL2L11* (BIM) and *BAX* was performed via targeted next generation DNA sequencing (tNGS) with Illumina AmpliSeq technology. We designed a custom amplicon panel with an amplicon size of up to 375 bases and a total panel size of 41 917 kb. Adjacent 5 intron bases were included to cover splice site mutations. Input of 200 ng was sufficient for libraries according to the AmpliSeq protocol. Sequencing was performed on an Illumina MiSeq™ with the 600-cycle MiSeq Reagent Kit v3.

### Cell viability assays

Cells were exposed to Venetoclax, Vincristine, Dexamethasone, Asparaginase, Daunorubicin, Staurosporine and Oligomycin or indicated combinations and cell death was analyzed either according to forward/side scatter criteria (cell lines, Supplementary Fig. 1E) or propidium iodide positivity (PDX samples). Cell death rates, EC_50_ values and dose-response matrix analyses were analyzed upon exposure to inhibitors for 72 h in cell lines and for 24 h in PDX samples. EC_50_ values were calculated using GraphPad Prism (version 9, Boston, Massachusetts USA, www.graphpad.com) based on percentages of cell death assessed by FSC/SSC criteria or propidium iodide positivity in flow cytometry as indicated. Percentages of cell death (y-axis) were plotted against drug concentrations (x-axis) (X = (log(X)) and dose-response curves were fitted using the function “*log(Agonist) vs. normalized response*”.

### Baseline BH3 profiling

BH3 profiling was performed as described [16–18]. Cells were permeabilized (digitonin), exposed to BH3-peptides and mitochondrial cytochrome c release was analyzed (anti-cytochrome c antibody). Cytochrome c median fluorescence intensities (MFI) were quantified and normalized to the MFIs of negative (DMSO) and positive (Alamethicin) controls.

### Immunoblotting

Proteins were isolated using a lysis buffer composed of 30 mM Tris-HCl pH 7.5, 150 mM NaCl, 1% Triton X-100 and 10% glycerol with the addition of cOmplete Proteinase Inhibitor Cocktail (Roche Diagnostics, Basel, Schweiz). Lysates were incubated on ice for 30 min and supernatant was collected after centrifugation at 14,000 rpm for 30 min at 4 °C. Immunoblots were developed using chemiluminescence and fluorescence, and densitometric analysis was performed using ImageJ Software.

### RNA-sequencing and gene set enrichment analyses

Total RNA was isolated from RS4;11 cells (Quick-RNA Miniprep Kit, Zymo Research, Freiburg, Germany) and RNA sequencing was performed (Core Facility Genomics, Ulm University, Germany). First, RNA purity was measured using a Nanodrop 1000 and RNA integrity (RIN-) values were determined by TapeStation analysis (Agilent, Technologies, Waldbronn, Germany). 200 ng of total RNA (measured by Qubit® analysis) was applied as input material for library construction using TruSeq RNA Sample Preparation Kit v2 (Illumina, San Diego, California, USA). Correct size distribution of the libraries was determined on a DNA1000 ScreenTape® using a Tape Station 4200 (Agilent). Multiplexed libraries were sequenced on an Illumina NextSeq550 using a NextSeq 500/550 High Output Kit v2.5 (75 Cycles) to generate ~30 M of single end 75 base pair reads per library. Differentially expressed gene-levels were identified by paired RNA analysis using the DESeq2 tool [19]. Gene set enrichment analysis (GSEA, v4.0.3; http://www.broadinstitute.org/gsea) was performed analyzing the enrichment of gene sets annotated in the Molecular Signature Database as described [20]. The ‘gene_set’ permutation configuration was used, gene sets with a NOM *p*-value ≤ 0.05 and a FDR q-value ≤ .05 were considered significant.

### Functional extracellular flux analysis

Cells were left untreated (BCP-ALL cell lines), treated with 10 nM and 100 nM VEN (RS4;11 VEN^sens^, RS4;11 VEN^ins^, BCP-ALL cell lines) or treated with 0.5, 1 and 2.5 µM VEN (PDX samples) for 3 h at 37 °C in RPMI-1640 medium (supplemented with 20% fetal bovine serum, 1% L-Glutamine and 1% Penicillin/Streptomycin; Gibco). XFe96 cell culture microplates (Agilent Technologies) were coated with 22.4 µg/ml Cell-Tak (Corning®) and RS4;11 VEN^sens^, RS4;11 VEN^ins^, BCP-ALL cell lines or PDX samples were plated (100,000 alive cells/well) in bicarbonate-free RPMI medium containing 5 mM HEPES, 10 mM glucose, 1 mM pyruvate, 2 mM glutamine into 96-well cell culture plates (XFe96, Agilent Technologies). Oxygen consumption rates (OCR) were measured using a Seahorse XFe96 Flux Analyzer (Agilent Technologies). Uncoupled (proton leak) respiration was profiled by injecting 1.25 mM Oligomycin (an ATP synthase inhibitor) and full substrate oxidation capacity was determined by injecting 0.75 µM carbonylcyanide-p-trifluoromethoxyphenylhydrazone (FCCP, a chemical uncoupler). Non-mitochondrial respiration was determined by injecting 0.5 mM antimycin A and 0.5 mM rotenone (inhibiting electron flux through complex I and III). OCRs were determined by plotting the partial oxygen pressure against time. The data obtained were normalized based on the cell numbers through Janus Green incorporation [21].

### Determination of mitochondrial mass and membrane potential

Cells were subjected to staining with 2 μg/ml Tetramethylrhodaminemethylesterperchlorate (TMRM, Sigma-Aldrich, St. Louis, Missouri, USA) or 50 nM MitoTracker Green (ThermoFisher Scientific) and incubated for 30 min at 37 °C. After washing (PBS), MFIs were measured (Attune NxT Flow Cytometer) and normalized to unstained control samples.

### Transmission electron microscopy

PDX cells were centrifuged and subsequently fixed in a solution containing phosphate buffer pH 7.3, 2.5% glutaraldehyde and 1% saccharose. Following fixation, the cells underwent a series of phosphate buffer washes (5 times,) and were then postfixed with 2% aqueous osmium tetroxide. Cells were dehydrated (graded series of 1-propanol), blockstained (1% uranyl acetate) and embedded in Epon (Sigma-Aldrich). Ultrathin sections (70 nm) were collected on copper grids, contrasted with 0.3% lead citrate for 1 min and subsequently imaged (JEM-1400 TEM, Jeol, Freising, Germany).

### qRT-PCR analyses

qRT-PCR analyses were conducted utilizing SsoAdvanced Universal SYBR Green Supermix (BioRad, Hercules, California, USA) using a Bio-Rad CFX Connect Real-Time PCR Detection System (95 °C for 30 s, 40 cycles of 95 °C for 5 s, corresponding melting temperatures for 30 s).

### Analysis of mitochondrial DNA content

DNA of PDX samples was isolated (QIAamp DNA Blood Mini Kit, Qiagen, Hilden, Germany) according to the manufacturer´s protocol. Nuclear and mitochondrial DNA content was quantified by real-time qPCR (specific primer probeset, Universal probe library 75 and 53, Roche Diagnostics). Mitochondrial to nuclear ratios were calculated as a relative measure of mitochondrial DNA content.

### Statistical analysis

Statistical analyses were performed with GraphPad Prism software (version 9). Data obtained from replicate analyses with numbers of biological and/or technical replicates as indicated in the corresponding figure legends were analyzed by two-sided T-test assuming equal variances, Spearman correlation or Chi-square test as indicated. Combination effects and synergy scores were analyzed (Synergyfinder, https://synergyfinder.fimm.fi/synergy/20240318114830462623/) using the Bliss independence model [22–24].

## Results

### Modeling VEN-specific apoptosis resistance

**Fig. 1:**
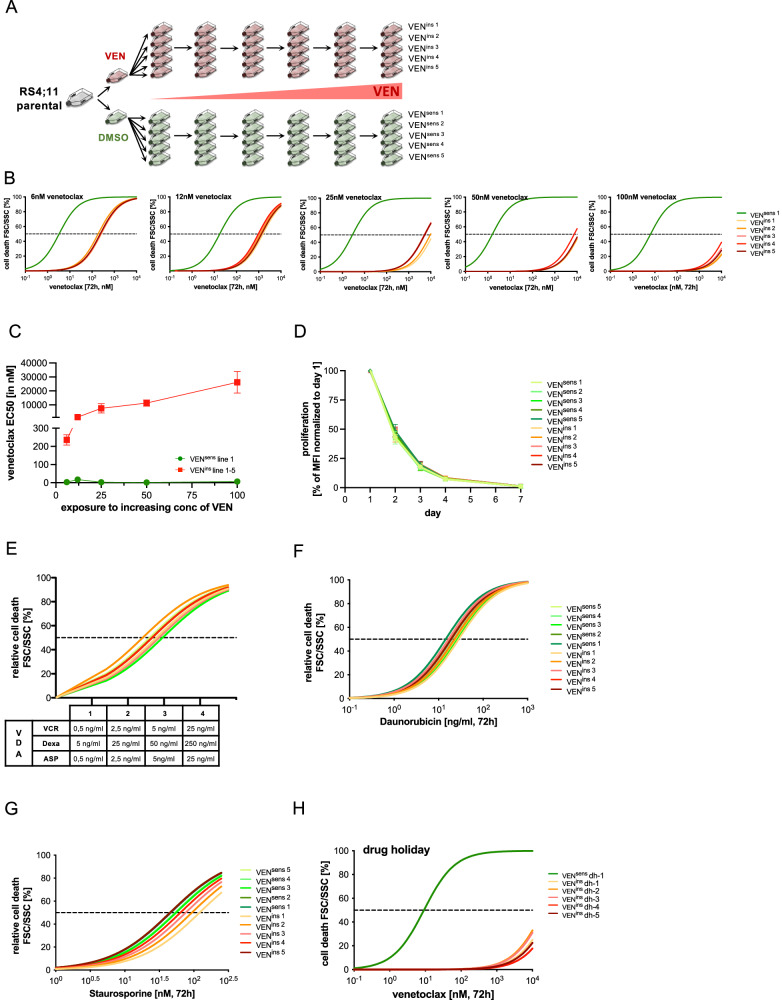
Characterization of VEN-resistance development in the BCP-ALL cell line RS4;11. **A**–**C** BCP-ALL cell line RS4;11 was cultured and treated with increasing concentrations of VEN (ranging from 6 nM, 12 nM, 25 nM, 50 nM and 100 nM) or the corresponding concentrations of solvent DMSO over time. Five DMSO control and VEN-resistant lines were generated each over a period of 8 months of continuous treatment. To test VEN resistance development, cells were treated with increasing concentrations of VEN (DMSO, 0.1 nM; 1 nM; 10 nM; 50 nM; 100 nM; 500 nM; 1 µM; 10 µM and 25 µM) for 72 h (*N* = 5 biological replicates/*N* = 1 in technical triplicates). Determination of half maximal effective concentration values (EC_50_) of VEN^sens^ line 1 compared to VEN^ins^ line 1-5 (FSC/SSC criteria, *N* = 1 in technical triplicates ± standard deviation) showed increasing EC_50_ values from 6.4 nM to 35.2 µM over time in all VEN treated lines. **D** VEN^sens^ and VEN^ins^ RS4;11 lines were stained with 1 μM CellTrace™ Violet and median fluorescence intensities (MFI) were determined every 24 h from day 1 to day 7. MFI values were normalized to MFIs of day 1 (*N* = 3 in triplicates, ± standard deviation). **E** VEN^sens^ and VEN^ins^ RS4;11 lines were treated with DMSO or the combination of Asparaginase, Dexamethasone and Vincristine for 72 h as stated. Cell death was assessed using FSC/SSC criteria and normalized to corresponding DMSO control. (*N* = 3 in triplicates). **F**, **G** VEN^sens^ and VEN^ins^ RS4;11 lines were incubated with increasing concentrations of Daunorubicine (DMSO; 0.1 ng/ml; 1 ng/ml; 10 ng/ml; 50 ng/ml; 100 ng/ml; 500 ng/ml and 1 000 ng/ml) and Staurosporine (DMSO; 1 nM; 5 nM; 10 nM; 25 nM; 50 nM; 100 nM; 250 nM) for 72 h. EC_50_ values were determined by FSC/SSC criteria and normalized to corresponding DMSO control (*N* = 3 in triplicates). **H** VEN^sens^ and VEN^ins^ RS4;11 lines were cultured under drug holiday conditions for 20 weeks. Cells were incubated with increasing concentrations of VEN (DMSO; 0.1 nM; 1 nM; 10 nM; 50 nM; 100 nM; 500 nM; 1 µM; 10 µM and 25 µM) for 72 h. Cell death was assessed using FSC/SSC criteria comparing VEN^sens^ line 1 and and VEN^ins^ RS4;11 lines 1-5 (*N* = 1 in technical triplicates).

To model VEN-resistance in BCP-ALL, we used the highly VEN-sensitive RS4;11 cell line and generated five lines by parallel exposure to increasing concentrations of VEN over time with five control lines exposed to corresponding solvent concentrations (Fig. 1A). VEN-sensitivities were consecutively analyzed (half maximal effective concentrations, EC_50_), showing a nearly thousand-fold increase in VEN-insensitivity in all exposed lines (Fig. 1B, C, Supplementary Fig. 1A, B). Compared to controls, the VEN-insensitive lines showed unchanged proliferation and maintained sensitivity to chemotherapeutic drugs typically employed in ALL induction therapy (combination of Vincristine/Dexamethasone/Asparaginase or Daunorubicin), as well as the apoptosis inducer Staurosporine, indicating acquisition of VEN-specific apoptosis resistance within this model (Fig. 1D–G, Supplementary Fig. 1C). Interestingly, all VEN-resistant lines retained their insensitivity upon 20 weeks culture without VEN (drug holiday; Fig. 1H, Supplementary Fig. 1D). Thus, specific VEN-insensitivity is maintained independently of VEN-exposure.

### MCL-1 contributes to VEN-resistance

**Fig. 2:**
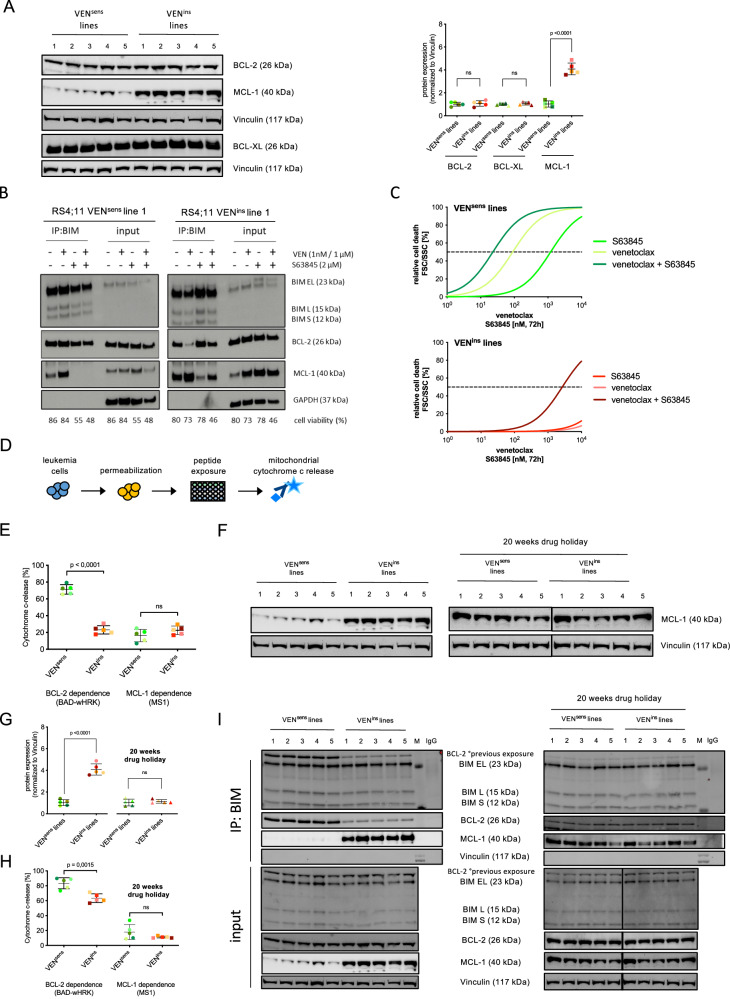
MCL-1, a possible mediator of VEN-resistance development. **A** Western Blot analysis of BCL-2, BCL-XL and MCL-1 protein expression comparing RS4;11 VEN^sens^ and VEN^ins^ lines under continuous VEN treatment. ImageJ analysis of protein expression normalized to Vinculin is shown. Unpaired two-tailed Student’s T-test was used to calculate *p*-values comparing five VEN^sens^ and five VEN^ins^ lines (± standard deviation is shown). **B** Immunoprecipitation (IP) analysis of BIM was performed in RS4;11 VEN^sens^ and VEN^ins^ line 1. Binding of BIM to BCL-2 or MCL-1 was analyzed upon exposure to VEN (1 nM in VEN^sens^ and 1 µM in VEN^ins^ cells), 2 µM S63845 or the combination of both inhibitors for 4 h. Cell death rates after drug treatment were analyzed by FSC/SSC criteria as stated. The IP:BIM lanes show the interaction of BIM with BCL-2 and MCL-1, while input lanes show the total proteins in the lysates (*N* = 1). **C** RS4;11 VEN^sens^ and VEN^ins^ lines were incubated with increasing concentrations of S63845, VEN (DMSO; 1 nM; 5 nM; 10 nM; 25 nM; 50 nM; 100 nM; 250 nM; 500 nM; 1 µM; 2.5 µM; 5 µM; 7.5 µM and 10 µM) or their combination for 72 h. Cell death was estimated using FSC/SSC criteria and normalized to the corresponding DMSO control. The mean of all five VEN^sens^ and VEN^ins^ lines per drug treatment is shown (*N* = 3, in triplicates). **D** A graphical schematic of baseline BH3 profiling analyzing RS4;11 VEN^sens^ and VEN^ins^ lines. **E** For baseline BH3 profiling, cells of all five RS4;11 VEN^sens^ and VEN^ins^ lines were permeabilized and incubated with the pro-apoptotic BH3-peptides BAD (indicating BCL-2 dependence), HRK (BCL-XL dependence) and MS1 (MCL-1 dependence). Cells were then fixed and stained with an anti-cytochrome c antibody binding exclusively to mitochondrial cytochrome c. Results show decreased BCL-2 priming in RS4;11 VEN^ins^ cells (BAD-HRK), while increased MS1 dependency upon VEN resistance was shown. (*N* = 5 biological replicates/*N* = 1 technical triplicates, ± standard deviation). Unpaired two-tailed Student’s T-test was used to calculate *p*-values comparing five VEN^sens^ to VEN^ins^ lines. **F** Western blot analysis of MCL-1 protein expression comparing RS4;11 VEN^sens^ and VEN^ins^ lines under continuous exposure to VEN (left panel) and RS4;11 VEN^sens^ and VEN^ins^ lines after 20 weeks drug removal (drug holiday, right panel) with respective loading controls (Vinculin) is shown. **G** Densitometric quantification of protein expression normalized to the corresponding Vinculin control and shown as a relative expression to the mean of the VEN^sens^ lines 1-5. Unpaired two-tailed Student’s T-test was used to calculate *p*-values comparing five VEN^sens^ and five VEN^ins^ lines (± standard deviation is shown). **H** Baseline BH3 profiling of 20 weeks drug holiday cells comparing all five RS4;11 VEN^sens^ and VEN^ins^ lines (± standard deviation is shown). **I** Immunoprecipitation (IP) analysis of BIM was performed in RS4;11 VEN^sens^ and VEN^ins^ line 1, also under 20 weeks drug holiday conditions. Basal binding of BIM to BCL-2 or MCL-1 was analyzed. The IP:BIM lanes show the interaction of BIM with BCL-2 and MCL-1, while input lanes show the total protein expression of whole cell lysates (*N* = 1).

Mutations in *BCL2* and other apoptosis regulators have been reported in VEN-resistance in other cancers [25, 26]. We performed targeted sequencing analysis comparing sensitive and resistant lines and found that VEN-resistance was not accompanied by acquisition of mutations in genes coding for BCL-2, BCL-XL, MCL-1, BIM or BAX. Interestingly, despite absence of *BAX*-mutations, BAX protein was not detected in VEN-resistant lines (Supplementary Fig. 2A). In cell lines and patient-derived xenograft samples however, BAX protein expression was found independently of VEN-sensitivity except no BAX expression in the VEN-insensitive NALM-6 line (Supplementary Fig. 2B, C). To further investigate mechanisms of VEN-resistance, we analyzed expression of the target molecule BCL-2 along with other anti-apoptotic BCL-2 family proteins (BCL-XL, MCL-1) comparing VEN-sensitive to insensitive cells. While expression of BCL-2 and BCL-XL remained unchanged, MCL-1 protein levels where significantly increased in VEN-resistant cells (Fig. 2A). Interestingly, upon withdrawal of VEN (drug holiday), MCL-1 expression returned to basal expression levels within 96 h (Supplementary Fig. 3A, B). At mitochondria, the apoptosis promoting proteins BAX and BAK are directly activated by pro-apoptotic BIM, which is counter-regulated by anti-apoptotic BCL-2 family proteins. To further address the mechanistic role of increased MCL-1 in VEN-insensitive ALL, we studied the interaction of BIM with BCL-2 and MCL-1. Co-immunoprecipitation of BIM together with BCL-2 and MCL-1 was performed in VEN-sensitive and insensitive cells, as well as after BCL-2 (VEN), MCL-1 (S63845) or combined inhibition using previously determined inhibitor concentrations resulting in similar cell death rates (Fig. 2B). In VEN-insensitive leukemia cells, a higher amount of MCL-1 was co-precipitated with BIM than in sensitive cells, indicating increased BIM to MCL-1 binding. Upon VEN exposure, displacement of BIM from BCL-2 and increased binding to MCL-1 was observed, particularly in VEN-insensitive cells. Accordingly, inhibition of MCL-1 disrupted the binding of BIM to MCL-1 along with a stronger compensatory binding of BCL-2. Importantly, combined BCL-2 and MCL-1 inhibition resulted in increased cell death induction.

Next, we assessed whether co-targeting of MCL-1 and BCL-2 would re-sensitize VEN-insensitive leukemia cells and investigated cell death induction by titrating concentrations of VEN, S63845, or their combination (Fig. 2C). VEN-sensitive cells displayed a moderate sensitivity to MCL-1 inhibition, which was further increased by combined inhibition compared to VEN alone (VEN^sens^ VEN-EC_50_ 89.41 nM vs VEN/S63845-EC_50_ 23.03 nM). In contrast, VEN-insensitive lines showed no sensitivity to either inhibitor alone but displayed clearly increased sensitivity to the combination compared to VEN, however not leading to complete re-sensitization (VEN^ins^ VEN-EC_50_ 144 359 µM vs. VEN/S63845-EC_50_ 2 677 µM).

Next, we addressed functional dependencies of the sensitive and insensitive leukemia cells on different mitochondrial apoptosis regulators (BH3 profiling) [18, 27, 28]. Cells were permeabilized and incubated with pro-apoptotic BH3-peptides that bind to the main anti-apoptotic BCL-2 family proteins BCL-2/BCL-XL and BCL-W (BAD), BCL-XL (wHRK), or MCL-1 (MS1). Early apoptosis induction was analyzed detecting mitochondrial cytochrome c release (Fig. 2D). A clearly decreased BCL-2 dependence was found in VEN-insensitive as compared to sensitive cells and there was an increase in MS1 priming, suggesting a higher MCL-1 dependence (Fig. 2E).

We also analyzed VEN-insensitive cells cultured without VEN for 20 weeks (drug holiday). Interestingly, despite remaining VEN-insensitivity with similar EC_50_ values, expression of MCL-1 returned to low levels (Fig. 2F, G). Functionally, these cells were characterized by decreased BCL-2 dependency, while a similar MCL-1 dependency and binding to BIM was observed when compared to VEN-sensitive cells (Fig. 2H, I).

Taken together, VEN-insensitive leukemia cells are characterized by increased MCL-1 expression and binding of pro-apoptotic BIM. However, further inhibition of MCL-1 did not result in complete re-sensitization. Although VEN-insensitive cells have lost their dependence on BCL-2, their dependence on MCL-1 was only slightly increased, challenging the idea that MCL-1 plays a major role in VENinsensitivity. Along this line, drug holiday lines with retained insensitivity showed normalized levels of MCL-1, less MCL-1 dependency and reduced binding of pro-apoptotic BIM, pointing to different or additional mechanisms of VEN-insensitivity other than MCL-1.

### Mitochondrial metabolic reprogramming upon VEN-insensitivity

**Fig. 3:**
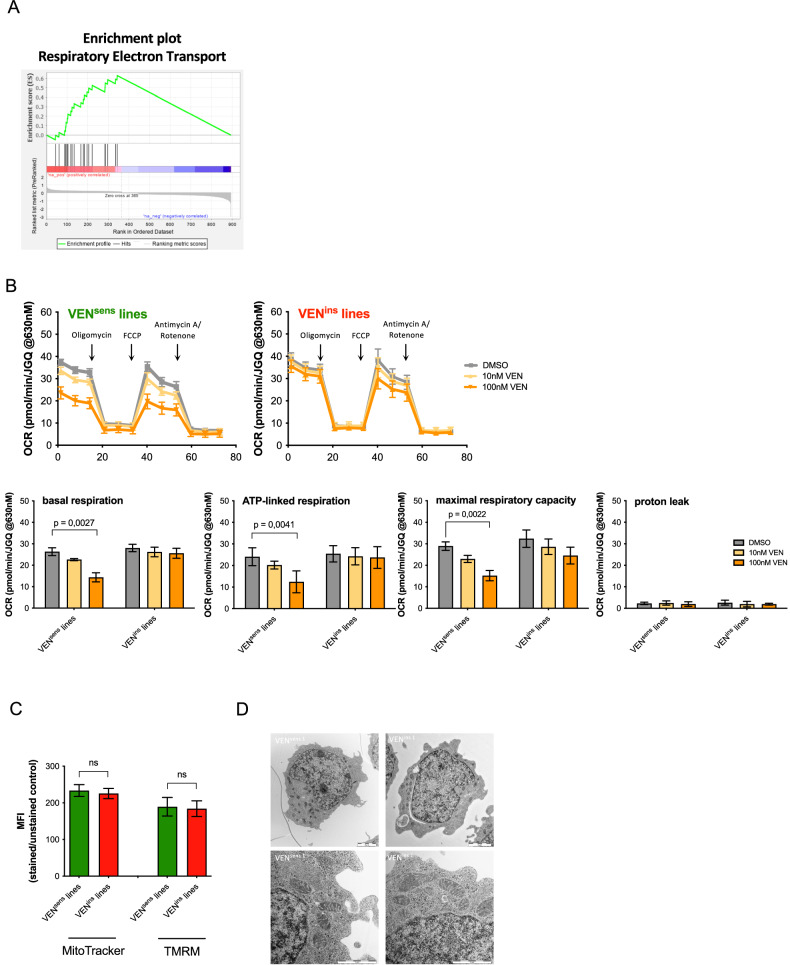
Altered metabolic profile, a hallmark of VEN resistance development. **A** Paired RNA-*Seq* analysis of five RS4;11 VEN^sens^ compared to five VEN^ins^ lines. Gene set enrichment analysis (GSEA, v4.0.3; http://www.broadinstitute.org/gsea) was performed to analyze the enrichment of gene sets annotated in the Molecular Signature Database as described. The ‘gene_set’ permutation configuration was used, and gene sets with a NOM *p*-value ≤ 0.05 and a FDR q-value ≤ 0.05 were considered significant. The enrichment plot of the Respiratory Electron Transport gene set is shown. **B** Basal oxygen consumption was measured in an XFe96 flux analyzer (Agilent) after treatment with DMSO or 10 nM and 100 nM VEN for 3 h. Mean values of five RS4;11 VEN^sens^ and VEN^ins^ lines are shown (*N* = 1, in six technical replicates, ± standard error of mean). Uncoupled respiration was profiled by injection of 1.25 μM Oligomycin and full respiration capacity was determined by injecting 0.75 μM carbonylcyanide-p-trifluoromethoxyphenylhydrazone (FCCP). Non-mitochondrial respiration was determined by injecting 0.5 μM antimycin A and rotenone, each. Oxygen consumption rates (OCRs) were determined by plotting the partial oxygen pressure against time. Basal respiration was analyzed by subtracting the minimal OCR after antimycin A/rotenone injection from the basal OCR. Data were normalized to cell number by Janus Green staining. Unpaired two-tailed Student’s T-test was used to calculate *p*-values. **C** To determine changes in mitochondrial membrane potential or mitochondrial mass, cells were stained with 2 μg/ml Tetramethylrhodaminemethylesterperchlorate (TMRM, Sigma-Aldrich) or 50 nM MitoTracker Green (ThermoFisher Scientific). Means of five RS4;11 VEN^sens^ and VEN^ins^ lines are shown and median fluorescence intensities (MFI) were determined and normalized to MFIs of unstained controls (*N* = 3 in triplicates, ± standard deviation). Unpaired two-tailed Student’s T-test was used to calculate *p*-values. **D** Mitochondrial morphology of RS4;11 VEN^sens^ line 1 and VEN^ins^ line 1 were analyzed by electron microscopy imaged in a JEM-1400 TEM (Jeol).

To further elucidate mechanisms of VEN-resistance, transcriptome profiles of VEN-sensitive and insensitive lines were analyzed and compared (VEN-sensitive, *N* = 5 and VEN-insensitive *N* = 5). Analyses of the identified differentially regulated genes using gene set enrichment analysis (GSEA) revealed a significant enrichment of gene sets annotated to the citric/tricarboxylic acid cycle and the respiratory electron transport chain, pointing to increased mitochondrial metabolism in VEN-resistant cells (Fig. 3A, Supplementary Fig. 4). Based on these findings, we investigated metabolic profiles and analyzed oxygen consumption rates (OCR, Agilent XF Mitostress Test) comparing VEN-sensitive to insensitive leukemias with or without prior exposure to VEN at low concentrations (10 and 100 nM, three hours), which did not yet induce mitochondrial apoptosis signaling as shown by unchanged mitochondrial membrane potential and cytochrome c release (Supplementary Fig. 5). While no differences of OCR were observed in the absence of VEN, basal respiration, ATP-linked respiration, as well as the maximal respiratory capacity were found to be significantly impaired in VEN-sensitive cells in contrast to sustained high oxidative metabolism upon low-dose VEN exposure in resistant cells (Fig. 3B).

Given these functional differences in mitochondrial metabolic profiles, we assessed mitochondrial mass and membrane potential but did not detect differences (Fig. 3C). In mitochondria, electron transport chain reactions occur at the cristae requiring correct cristae formation. However, no differences in mitochondrial structure and morphology as analyzed by electron microscopy were observed in sensitive and insensitive lines (Fig. 3D).

### Intrinsic VEN-resistance is characterized by increased mitochondrial metabolic activity

**Fig. 4:**
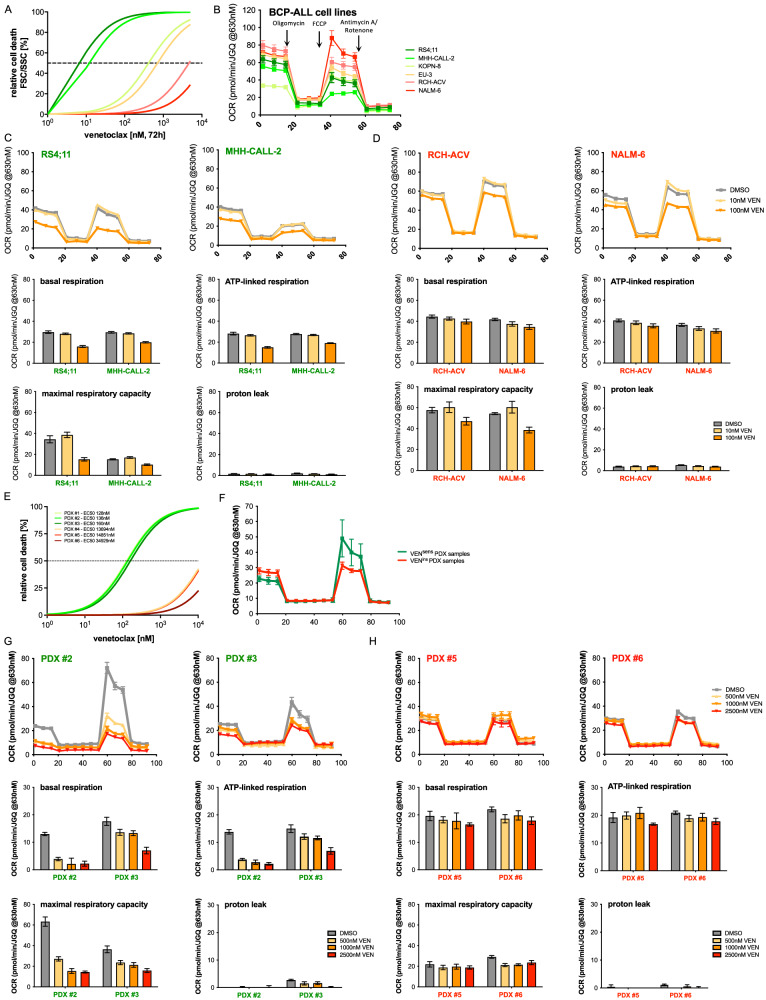
Intrinsic VEN-resistant BCP-ALL cell lines and patient-derived xenograft samples are characterized by increased metabolic activity. **A** Six BCP-ALL cell lines were incubated with increasing concentrations (DMSO; 10 nM; 100 nM; 500 nM; 1 μM; 5 μM) of VEN for 72 h before analysis of cell death by FSC/SSC criteria and normalized to the corresponding DMSO control (*N* = 3, in triplicates). **B** The basal metabolic profile of six BCP-ALL cell lines is shown. Basal oxygen consumption was measured in an XFe96 flux analyzer (Agilent) (*N* = 6 biological replicates/*N* = 1 in five technical replicates, ± standard error of mean). Uncoupled respiration was profiled by injection of 1.25 μM Oligomycin and full respiration capacity was determined by injecting 0.75 μM carbonylcyanide-p-trifluoromethoxyphenylhydrazone (FCCP). Non-mitochondrial respiration was determined by injecting 0.5 μM antimycin A and rotenone, respectively. Oxygen consumption rates (OCRs) were determined by plotting the partial oxygen pressure against time. Basal respiration was analyzed by subtracting the minimal OCR after antimycin A/rotenone injection from the basal OCR. Data were normalized to cell number by Janus Green staining. **C**, **D** Metabolic profile of two VEN^sens^ (**C**) compared to two VEN^ins^ (**D**) BCP-ALL cell lines after 3 h DMSO or 10 nM and 100 nM VEN treatment is shown (*N* = 1 in five technical replicates ± standard error of mean). **E** Six PDX samples were exposed to increasing concentrations (1 nM, 5 nM, 10 nM, 50 nM, 100 nM, 250 nM, 500 nM, 1 μM, 5 μM and 10 μM) of VEN for 24 h. Relative cell death rates were assessed by propidium iodide (PI) staining and normalized to DMSO controls (*N* = 1, in triplicates). **F** The basal metabolic profile of three VEN^sens^ compared to three VEN^ins^ PDX samples is shown (mean of *N* = 3 + 3 biological replicates, *N* = 1 in five technical replicates ± standard error of mean). **G**, **H** Metabolic profiles of two VEN^sens^ (**G**, labelled in green), and two VEN^ins^ (**H**, labelled in red) PDX samples after 3 h exposure to DMSO or 500 nM, 1000 nM or 2500 nM VEN (*N* = 1 in five technical replicates ± standard error of mean).

In addition to acquired VEN-insensitivity modeled in RS4;11 cells, we also addressed intrinsic VEN-resistance in a set of BCP-ALL cell lines characterized by VEN-resistance or sensitivity (*N* = 6, Fig. 4A, Supplementary Fig. 6, 10). Corresponding to sustained high metabolic profiles in VEN-insensitive RS4;11 cells, oxidative metabolism of VEN-resistant lines was also found to be increased (Fig. 4B). Upon VEN exposure with concentrations not inducing apoptosis (Supplementary Fig. 7), VEN-sensitive cell lines showed that basal respiration, ATP-linked respiration and maximal respiratory capacity were impaired, while oxidative metabolism was sustained at higher levels in VEN-insensitive lines (Fig. 4C, D; Supplementary Fig. 8A), in line with our findings in modeled aquired insensitivity.

BCP-ALL shows heterogenous sensitivities to VEN, with some leukemias showing low EC_50_ values indicating high sensitivity, while others show intrinsic resistance, as seen in a series of primary patient-derived xenograft (PDX) samples (Supplementary Figs. 8B, 9) [28]. Importantly, primary PDX BCP-ALL samples with high VEN-sensitivity demonstrated clearly lower metabolic rates compared to samples with strong resistance (Fig. 4E, F) and VEN-insensitivity was significantly correlated to higher basal and ATP-linked respiration rates (Supplementary Fig. 8C). In line with our findings in cell lines, exposure of PDX leukemias to VEN at concentrations (500, 1,000 and 2 500 nM; three hours) not inducing mitochondrial apoptosis signaling (Supplementary Fig. 11) resulted in significantly impaired OCR and ATP production in VEN-sensitive PDX leukemias, while metabolic activity stayed at high levels in VEN-resistant cells (Fig. 4G, H).

In summary, increased and sustained mitochondrial metabolic activity is a feature of VEN-resistant ALL, both in cell line models of acquired insensitivity and in cell lines and primary leukemia cells with intrinsic VEN-resistance.

### VEN-resistant leukemia is characterized by Increased mitochondrial content and elongated mitochondrial structures

**Fig. 5:**
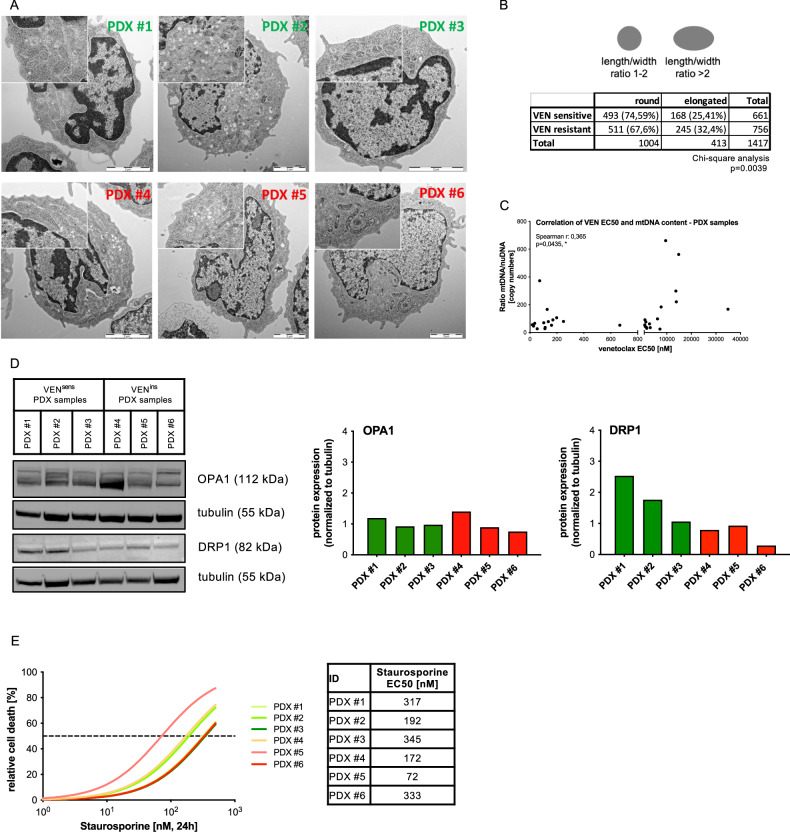
Intrinsic VEN-resistant PDX samples show elongated mitochondrial structures and increased mitochondrial DNA content. **A** Mitochondrial morphology of three sensitive and three highly VEN-resistant PDX samples were analyzed by electron microscopy imaged in a JEM-1400 TEM (Jeol). **B** The mitochondrial length/width ratio of 83 cells (661 and 756 Mitochondria) was analyzed using the ImageJ software. Chi-square analysis comparing the distribution of elongated mitochondria in VEN^sens^ and VEN^ins^ PDX samples is shown. **C** Mitochondrial DNA content was assessed by relative quantification and correlated to VEN EC_50_ values (Spearman correlation; r, correlation coefficient; p, significance). **D** Western Blot analysis of OPA1 and DRP1 is shown comparing three VEN-sensitive and three VEN-resistant PDX samples. Protein expression was normalized to tubulin using ImageJ analysis. **E** Three VEN-sensitive and three highly VEN-resistant PDX samples ( > 1 µM EC_50_) were incubated with increasing concentrations of Staurosporine (DMSO; 1 nM; 10 nM; 50 nM; 100 nM; 500 nM) for 24 h. Relative cell death rates were assessed by propidium iodide (PI) staining and normalized to DMSO controls. (*N* = 1 technical triplicates).

Next, we studied mitochondrial morphology by electron microscopy in primary PDX leukemia samples (VEN-resistant/high mitochondrial metabolism or VEN-sensitive/low mitochondrial metabolism, *N* = 3 each). VEN-resistant leukemia cells showed a higher ratio of mitochondrial length to width indicating elongated mitochondrial structures in VEN-resistant compared to VEN-sensitive leukemia cells and more mitochondria with an elongated structure (ratio of length to width >2) were found in VEN-resistant ALL (Chi-square analysis *p* = 0.0039; Fig. 5A, B). Mitochondria harbor their own mitochondrial DNA (mtDNA), which encodes proteins important for the assembly and activity of respiratory complexes. Mitochondrial DNA (mtDNA) content was analyzed in our cohort of PDX BCP-ALL samples (*N* = 31) showing a significant positive correlation between mtDNA levels and VEN EC_50_ values, consistent with increased mitochondrial metabolism observed in VEN-resistant leukemias (Fig. 5C). However, there were no significant differences between VEN-sensitive and resistant cells analyzing the overall number of mitochondria per cell, mitochondrial mass (MitoTracker incorporation), polarization of the mitochondrial outer membrane (TMRM positivity), or expression of the mitochondrial transcription factor A (TFAM), which regulates transcription, replication, and packaging of mtDNA [29] (Supplementary Fig. 12A), arguing against an overall increased mitochondrial biogenesis in VEN-resistant ALL. Mitochondria are highly dynamic organelles constantly changing their structure by fusion or fission in response to cellular demands and changed mitochondrial structures can be caused by dysregulated fusion or fission processes. The coordination of inner and outer mitochondrial membrane fusion is controlled by the GTPase Optic atrophy protein 1 (OPA1), Mitofusin (MFN) 1 and 2, while mitochondrial fission is regulated by Dynamin-related protein 1 (DRP1), a large dynamin-like GTPase that is recruited to the outer membrane by different adaptor proteins like mitochondrial fission 1 protein (FIS1) or mitochondrial fission factor (MFF) [30]. Comparing leukemias with different VEN-sensitivities, no significant differences in transcript expression of the fusion (*OPA1*, *MFN1*, *MFN2*) or fission (*DRP1*, *MFF*) factors were found (Supplementary Fig. 12B). However, a lower expression of fission factor DRP1 was found on protein level in VEN-resistant PDX samples (Fig. 5D), pointing to a shifted balance towards increased fusion and decreased fission resulting in elongated mitochondrial morphologies.

Importantly, also in PDX ALL samples the insensitivity is VEN-specific with comparable sensitivities to increasing concentrations of the apoptosis-inducing agent Staurosporine in VEN-sensitive and insensitive PDX-leukemias (Fig. 5E, Supplementary Fig. 13). This further highlights that differences in metabolic activity and mitochondrial structures identified in VEN-sensitive and insensitive cells are specific and not associated with an overall changed susceptibility to apoptosis induction.

Taken together, we observed significantly larger and elongated mitochondria in VEN-resistant ALL, along with increased mtDNA and lower protein expression of the mitochondrial fission regulator DRP1, consistent with the functional analyses showing an increased mitochondrial metabolism in VEN-resistant leukemias.

### Synergistic anti-leukemia activity upon combined inhibition of BCL-2 and oxidative phosporylation

Based on our findings of altered mitochondrial morphology and increased metabolic activity, we extended our analyses and evaluated whether the combination of BCL-2 inhibition together with OxPhos inhibition would overcome VEN-insensitivity. Using the complex V/ATPase subunit inhibitor Oligomycin, we first investigated cell death induction in cell lines with modeled VEN-insensitivity. Exposure to increasing concentrations of Oligomycin did not lead to substantial induction of cell death, but when combined with BCL-2 inhibition a significant, dose-dependent cell death induction was observed in VEN-insensitive lines and in drug holiday cells (Supplementary Fig. 16A, B). In contrast, combinations of Oligomycin together with different cell death inducing agents as the MCL-1 inhibitor S63845, Daunorubicin, Staurosporine or Vincristine-Dexamethasone-Asparaginase failed to synergize in cell death induction (Supplementary Figs. 14, 15).

Next, we investigated ALL lines with intrinsic VEN-insensitivity and also found high cell death induction upon combined BCL-2 and OxPhos inhibition (Supplementary Fig. 16C, D). Interestingly, two of the VEN-sensitive lines showed high sensitivity to Oligomycin on its own, in line with lower mitochondrial metabolism in VEN-sensitive as compared to higher metabolism in VEN-insensitive leukemias.

**Fig. 6:**
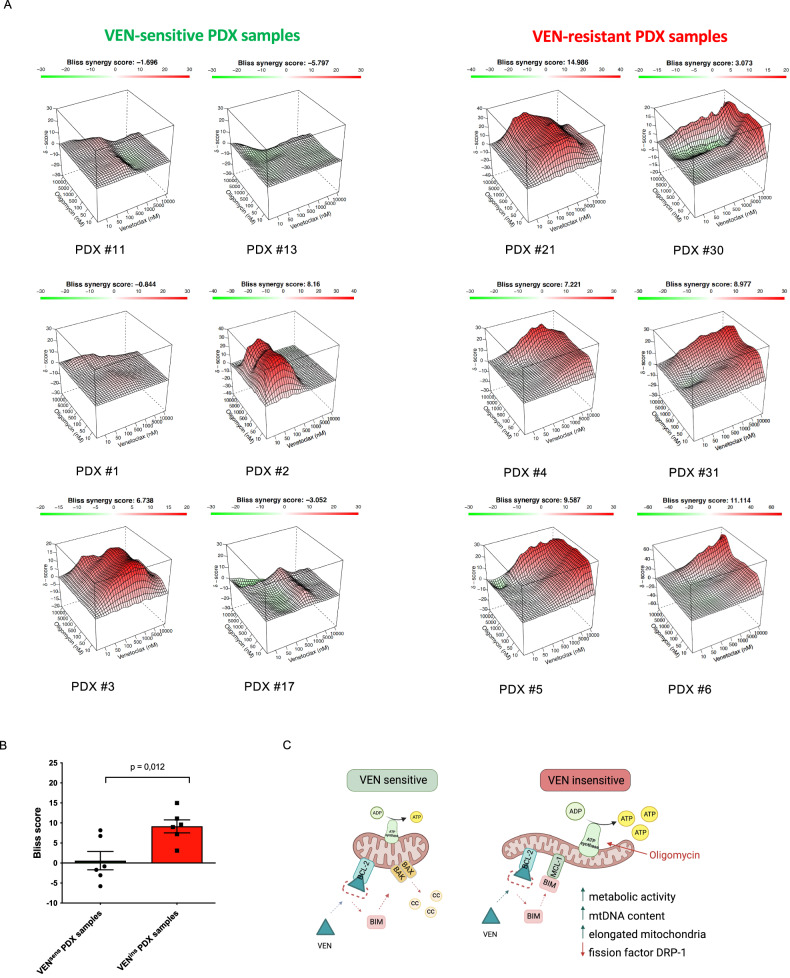
Co-treatment of BCL-2 and the OxPhos pathway re-sensitizes VEN-resistant PDX samples. **A** Twelve PDX samples were incubated with increasing concentrations of VEN (DMSO; 10 nM; 50 nM; 100 nM; 1 µM; 5 µM; 10 µM), Oligomycin (DMSO; 10 nM; 50 nM; 100 nM; 1 µM; 5 µM; 10 µM) or titrated in an one to one matrix combination and incubated for 24 h. Cell death was analyzed using flow cytometry after propidium iodide staining. Interaction landscapes of the dose-response matrix analyses are shown. To estimate synergy, δ-scores were calculated using synergyfinder. Synergistic effects are shown in red, additive effects in white and antagonistic effects in green. The Bliss synergy score shown indicates the average synergy score across the dose-response matrix. **B** Comparing VEN^sens^ and highly VEN^ins^ PDX samples (EC_50_ > 1 µM). Unpaired two-tailed Student’s T-test was used to calculate *p*-values (± standard deviation is shown). **C** When VEN^sens^ samples were treated with VEN, pro-apoptotic BIM is displaced from BCL-2 to MCL-1 thereby initiating BAX/BAK pore formation, cytochrome c release and cell death induction. Conversely, when VEN^ins^ samples are treated with VEN, BIM is liberated from BCL-2 but increased MCL-1/BIM binding is present. Additionally, VEN^ins^ samples are characterized by increased metabolic activity, increased mtDNA content and elongated mitochondrial structures. Of note, co-treatment of VEN and OxPhos inhibitors re-sensitizes VEN-resistant cells to cell death induction. Created with BioRender.com.

Further, we addressed anti-leukemia activity of combined BCL-2 and OxPhos inhibition in a series of PDX BCP-ALL samples characterized by different VEN-sensitivities titrating a dose-response matrix with combinations of increasing concentrations of VEN or Oligomycin. Synergistic activity was observed in all tested leukemias, however to varying degrees, as indicated by the Bliss synergy score, an estimate of synergistic drug activity (Fig. 6A). Interestingly, VEN-insensitive samples showed significantly higher synergy scores thus indicating a clearly higher susceptibility of VEN-resistant leukemias (Fig. 6B) to combined BCL-2 and OxPhos inhibition.

Taken together, by characterizing ALL cell lines with modeled VEN-insensitivity and primary samples with intrinsic VEN-resistance we identified altered gene expression profiles pointing to increased mitochondrial metabolism in leukemias with VEN-insensitivity. These leukemias showed, along with altered mitochondrial morphology and increased content of mitochondrial DNA, a sustained high metabolic activity in functional metabolic profiling analyses (Fig. 6C). Importantly, this increased high metabolic activity in VEN-resistant cells can serve as a target for therapeutic intervention and co-targeting of BCL-2 and OxPhos showed synergistic anti-leukemia activity, thus providing a basis for further preclinical and potential clinical evaluation.

## Discussion

Anti-apoptotic BCL-2 family proteins critically regulate the mitochondrial apoptosis process by counteracting its induction through direct binding to pro-apoptotic BH3-only proteins or by preventing BAX and BAK activation [2, 3]. Imbalance of pro- and anti-apoptotic BCL-2 family member expression leads to deregulated cell death pathways and is commonly found in hematological malignancies including BCP-ALL [31–33]. Among the anti-apoptotic proteins, BCL-2 plays an essential role in mediating chemotherapy resistance, thereby providing an important therapeutic target [34, 35]. The BH3-mimetic Venetoclax (VEN) selectively binds to the BH3-binding groove of BCL-2, thereby abrogating its inhibitory effect on BAX/BAK, ultimately leading to the execution of mitochondrial apoptosis signaling [7]. VEN offers advanced treatment options for patients with aggressive and poor prognosis lymphoid malignancies [8, 9, 11] and we and others have highlighted the ex vivo efficacy of VEN in BCP-ALL [10, 35–37]. In addition, first case studies in particular poor outcome (*TCF3*::*HLF*-rearranged) [38] BCP-ALL and ongoing investigation in first clinical trials [39, 40] show improved patient outcome. However, clinical VEN activity comes with a challenge: VEN-resistance, either due to intrinsic cellular features or secondary resistance, leads to disease progression and treatment failure [13, 14].

In this study, we addressed mechanisms of VEN-resistance in BCP-ALL, both in acquired and intrinsic resistance. Generating a cell line model of acquired VEN-resistance allowed us to characterize molecular changes in detail. While proliferation rates and sensitivity to chemotherapeutic drugs and other cell death inducing agents were unchanged, VEN-insensitivity of cells (in five biological replicates) was stable, irrespective of exposure to VEN. Previous studies have shown that adaptation of tumor cells to VEN is characterized by different mechanisms resulting in functional loss of pro-apoptotic regulators like BAD, BAX, NOXA and PUMA or by a shift in dependency from BCL-2 to other anti-apoptotic proteins like MCL-1 [26, 41–50].

We observed lost BAX protein expression in ALL cells with acquired but not intrinsic VEN-resistance, in line with a report on pathogenic *BAX* mutations in acute myeloid leukemia cases with acquired resistance after VEN therapy but not in primary VEN-refractory patients [26]. Loss of BAX has been described in VEN-resistant CLL cell lines and patient samples without concurrent *BAX* mutations [48], as we have observed upon modeled VEN-resistance in ALL. Interestingly, BAX knockout in B-cell lymphoma cells did not result in decreased VEN-sensitivity [50], further indicating that BAX-deficiency (due to gene alteration or other mechanisms) on its own impairs VEN-sensitivity in some contexts but does not seem to be a general mechanism of VEN-resistance.

The sequestration of pro-apoptotic BIM by MCL-1 after VEN-induced release from BCL-2 prevents apoptosis induction [51, 52]. Our data confirm the compensatory role of MCL-1 with increased protein expression, slightly increased MCL-1 dependency, increased BIM to MCL-1 binding in co-precipitation studies and partial sensitization of VEN-resistant cells by concomitant MCL-1 inhibition. However of note, VEN-insensitive cells cultured without VEN (drug holiday) did not show this mechanism of increased MCL-1, suggesting that MCL-1 might contribute to, but is not the key factor driving VEN-resistance in BCP-ALL.

By transcriptome analysis and functional characterization, we identified higher basal respiration and ATP-linked respiration rates in VEN-resistant ALL. When challenged by exposure to the BCL-2 inhibitor, VEN-sensitive samples showed a rapid reduction of basal and ATP-linked respiration, whereas VEN-resistant samples were able to compensate drug-induced impairment of mitochondrial metabolic activity, indicating reprogramming of mitochondrial metabolism in VEN-resistant leukemia. Interestingly, other studies have described metabolic changes associated with resistance to BCL-2 inhibition in other cancer models, consistent with our findings in BCP-ALL cells [53, 54]. In addition, BCL-2 proteins have been reported to control cellular bioenergetics in colon carcinoma cells, and BCL-2 inhibition by VEN or WEHI-539 reduced mitochondrial ATP production at concentrations that did not induce cell death [55].

Mitochondria are essential not only as the cell´s powerhouse, but are also critically involved in different cellular functions [56] and processes regulating mitochondrial function and architecture are modulated during cancer development and tumorigenesis [57, 58]. For instance, mitochondrial configuration is tightly regulated in response to the required cellular metabolism by events controlling mitochondrial fission and fusion. Increased fusion of mitochondria has been observed in situations of dependence on oxidative phosphorylation, starvation or drug-induced autophagy [59, 60]. In contrast, inhibited fission protects mitochondria from autophagic degradation and supports maximizing ATP production under stress conditions [59, 60]. We observed that VEN-resistant cells were characterized by enlarged and elongated mitochondria along with clearly higher levels of mitochondrial DNA. This was not accompanied by higher numbers of mitochondria, changed outer membrane potential or increased expression of mitochondrial transcription factor A (TFAM), arguing against an overall increased mitochondrial biogenesis in VEN-resistant ALL. Mitochondrial fusion and fission processes are mainly initiated and orchestrated by two important factors, GTPase Optic atrophy protein 1 (OPA1, fusion) and the dynamin-like GTPase Dynamin-related protein 1 (DRP1, fission) [30]. Elongated mitochondria result from either excessive fusion or blocked fission events. While VEN-resistant primary PDX-ALL showed clearly downregulated DRP1 fission factor, OPA1 was not differentially expressed when compared to sensitive samples. This is consistent with previous observations linking structural mitochondrial changes to cellular survival and has been targeted to sensitize myeloid leukemia to VEN treatment [61].

In line with our findings, different studies suggest that cancer cells shift to increased oxidative phosphorylation during tumor progression or drug resistance. For example, tumor biopsies from patients with progressed melanoma and MAPK inhibitor resistance showed increased mitochondrial biogenesis and mitochondrial bioenergetics [62] and in primary metastatic colorectal cancer higher levels of the transcription factor TFAM, mtDNA copy numbers and ADP-triggered oxygen consumption rate (OCR) were found as compared to non-metastatic primary cells [63]. Metastasized ovarian cancer showed dependence on high oxidative phosphorylation to maintain high proliferation [64] and in multiple myeloma activity of electron transport chain Complex I and II was associated with VEN-sensitivity [65].

These data together with our findings suggest that the increased mitochondrial OxPhos activity in VEN-resistant cells provides an achilles heel to bypass and overcome insensitivity to BCL-2 inhibition in cancer, including BCP-ALL. Accordingly, co-treatment with VEN and OxPhos inhibitors like Oligomycin significantly re-sensitized VEN-resistant samples leading to increased cell death. Particularly VEN-resistant ALL showed higher vulnerability to combined BCL-2 and OxPhos inhibition, pointing to a potential therapy target. However, due to its significant side effects, direct OxPhos inhibition is limited in clinical application and further evaluation of alternative strategies will be required.

In summary, modeling of VEN-resistance in BCP-ALL by in vitro drug exposure resulted in sustained and VEN-specific insensitivity, also in the absence of the drug. Transcriptome profiling revealed significantly differentially regulated genes annotated to key mitochondrial metabolic processes, including the respiratory electron transport chain and the citric/tricarboxylic acid cycle. Functionally, metabolic profiling analyses showed a reprogrammed, high mitochondrial metabolism in VEN-resistant leukemia, which was also found in VEN-insensitive, primary PDX-ALL samples. Accordingly, electron microscopy studies revealed altered mitochondrial morphology associated with disbalanced mitochondrial fusion/fission and higher mitochondrial DNA content. Given an increased mitochondrial metabolism in VEN-resistant leukemia, we investigated targeting of oxidative phosphorylation and found re-sensitization of resistant ALL to VEN warranting further evaluation for potential clinical application.

### Supplementary information

Supplementary Data

Supplementary Figures

Original Data File

## Data Availability

Gene expression data are deposited at the Gene Expression Omnibus (GEO) database (https://www.ncbi.nlm.nih.gov/geo/) and can be assessed through https://www.ncbi.nlm.nih.gov/geo/query/acc.cgi?acc=GSE245284.
