# Supplementary material for: Venetoclax resistance in acute lymphoblastic leukemia is characterized by increased mitochondrial activity and can be overcome by co-targeting oxidative phosphorylation
Source: Cell Death Dis. 2024 Jul 3;15(7):475. doi: 10.1038/s41419-024-06864-7 (PMC11222427; doi:10.1038/s41419-024-06864-7)
Supplement: Supplementary file 1 — Supplementary Data [file 41419_2024_6864_MOESM1_ESM.pdf]

Venetoclax Resistance in Acute Lymphoblastic Leukemia is characterized by Increased Mitochondrial Activity and Can Be Overcome by Co-targeting Oxidative Phosphorylation

Supplementary Methods:

BCP-ALL Cell Lines

RS4;11, MHH-CALL-2, KOPN-8, EU-3, RCH-ACV and NALM-6 cells were purchased (DSMZ, Germany) and cultured in RPMI-1640 medium (20 % fetal bovine serum, 1 % L-Glutamine, 1 % Penicillin/Streptomycin; 5 % CO_2, 37 °C). Lines were authenticated using short-tandem repeat (STR)-profiling (GenePrint® 10 System, Promega Madison, Wisconsin, USA) and regularly tested negative for Mycoplasma negativity (MycoAlert® Mycoplasma Detection Kit, Lonza, Basel, Switzerland).

VEN Resistance Development in RS4;11 Cell Line

Starting from the BCP-ALL cell line RS4;11, five VEN resistant lines were generated in parallel by exposure to increasing concentrations of VEN (Selleckchem, Houston, Texas, USA), over time (49 passages, 8 months of continuous treatment) and five control lines were exposed to corresponding concentrations of solvent (DMSO, Serva, Heidelberg, Germany). In the beginning, RS4;11 lines were exposed to very low VEN concentrations of 1 nM and cell viability was regularly determined by FSC/SCC (every 2-3 days, FACS measurement). As soon as all five lines showed viability over 90 %, VEN concentrations were increased (4 nM, 6 nM, 12 nM, 25 nM 50 nM and 100 nM). RS4;11 lines exposed to VEN showed different recovery times between two and eight weeks as summarized in Supplementary Figure 1A. At

increase of concentrations, half maximal effective concentrations (EC50) were determined (forward/side scatter criteria, Attune NxT Flow Cytometer). VEN exposed lines showed increasing EC_{50} values from 15.67 to 35.2 µM over time, indicating acquired resistance development in these cells.

BCP-ALL Patient-Derived Xenograft Samples

Primary leukemia samples of BCP-ALL patients were collected after written informed consent in accordance with the institution’s ethical review board. Patient-derived xenograft samples were generated by intravenous transplantation of ALL cells into female NOD/SCID mice (NOD.CB17-Prkdcscid, Charles River Laboratories, Wilmington, Massachusetts, USA) as previously described[1]. Immunophenotyping of PDX samples were done according to standard protocols using an LSR-II flow cytometer (BD Biosciences). Genetic alterations were assessed by Multiplex Ligation-dependent Probe Amplification (MLPA) and RT-PCR as previously described[2]. PDX cells were cultured in RPMI (Gibco Life Technologies, Carlsbad, California, USA) supplemented with 20 % FCS at 5 % CO2 and 37 °C. Animal experiments were approved by the Regierungspräsidium Tübingen (Tierversuchsanzeige Nr. V.48).

Celltrace Violet

Control and VEN resistant RS4;11 lines were stained with 1µM CellTrace™ Violet (Molecular Probes™ Thermo Fisher Scientific, Waltham, Massachusetts, USA CellTrace™ Violet Cell Proliferation Kit) as instructed by the manufacturer. Briefly 2 × 10^6 cells were stained for 20 min at 37 °C, staining was stopped by adding FCS for 5 min at 37 °C and washed with PBS followed by culturing in RPMI-1640 media (supplemented with 20 % fetal bovine serum, 1 % L-Glutamine, 1 %

Penicillin/Streptomycin) for indicated timepoints. CTV median fluorescence intensities (MFI) were determined (Attune NxT Flow Cytometer, Thermo Fisher Scientific) and normalized to MFIs of day 1.

Cell Viability Assays

Cells were exposed to Venetoclax, S63845, Staurosporine, Oligomycin, Daunorubicin (all Selleckchem), Vincristine, Dexamethasone and Asparaginase (kindly provided by the Pharmacy, Ulm University Medical Center) or indicated combinations and cell death was analyzed either according to forward/side scatter criteria (cell lines) or propidium iodide positivity (PDX samples). Cell death rates and half maximal effective concentration values (EC_{50}) were analyzed upon exposure to inhibitors for 72 hours in cell lines and for 24 hours in PDX samples (Attune NxT Flow Cytometer, Thermo Fisher Scientific).

Immunoprecipitation and Immunoblotting

Proteins were isolated using a lysis buffer (30 mM Tris-HCl pH 7.5, 2 mM KCl, 2 mM EDTA, 1 % Triton X-100, 10 % glycerol) supplemented with 1X cOmplete™ Proteinase Inhibitor Cocktail (Roche Diagnostics, Basel, Schweiz). Lysates were incubated on ice for 30 min and supernatant was collected after centrifugation at 14 000 rpm for 30 min at 4°C. Protein concentrations were assessed via Pierce™ BCA Protein Assay Kits (Thermo Fisher). For immunoprecipitation, cell lysates were incubated with 1µg of BIM Rabbit mAb (#2933 (C34C5), Cell Signaling Technology; Danvers, Massachusetts, USA; overnight, 4 °C) followed by Protein A Agarose beads (#9863S, Cell Signaling Technology; 2 h, 4 °C), afterwards beads were washed 5x with lysis buffer. Protein lysates or precipitates were mixed with Bolt™ LDS sample buffer (Invitrogen, Waltham, Massachusetts, USA) and Bolt™ sample reducing agent

(Invitrogen), heated for 10min at 70 °C and separated in Bolt^{TM} 4-12 % Bis-Tris Plus gels, following by a transfer to a nitrocellulose membrane (iBlot Gel Transfer Device, Invitrogen). Western Blot analysis of protein lysates and precipitates were performed using anti-BCL-2 (catalog #15071, clone 124), anti-BCL-XL (#2764, clone 54H6), anti-MCL-1 (#94296, clone D2W9E), anti-BIM (#2933, clone C34C5), anti-BAX (#2772), anti-BAK (#12105, clone D4E4), anti-DRP1 (#5391, clone D8H5), anti-OPA1 (#67589, clone D7C1A) (Cell Signaling Technology); anti-ß-Actin (#A5441, clone AC-15), anti-Vinculin (#VU505, clone 7F9) (Sigma-Aldrich); anti-Tubulin (#2148, Cell Signaling Technology) or anti-GAPDH (#ADI-CSA-33S-E, clone 1D4, Enzo) antibodies; and mouse anti-rabbit IgG-HRP (#sc-2357), goat anti-mouse IgG_1 (#sc-516102) (Santa Cruz); StarBright Blue 700 goat anti-mouse IgG (#12004158, clone M700), StarBright Blue 700 goat anti-rabbit IgG (#12004161, clone R700) (Bio-Rad Laboratories, Hercules, California, USA) secondary antibodies. Immunoblots were developed using chemiluminescence and fluorescence and densitometric analysis was performed using ImageJ Software.

Determination of Mitochondrial Mass and Membrane Potential

Cells were stained with 2 µg/ml Tetramethylrhodaminemethylesterperchlorate (TMRM, Sigma-Aldrich) or 50nM MitoTracker^{TM} Green (ThermoFisher Scientific) and incubated for 30 min at 37 °C. Cells were washed with PBS and Mitochondrial Membrane Potential (TMRM) or Mitochondrial Mass (MitoTracker^{TM} Green) was analyzed (Attune NxT Flow Cytometer). Median fluorescence intensities (MFI) were determined and normalized to MFIs of unstained controls.

Intracellular Cytochrome C Staining

Cells were permeabilized (Digitonin) and mitochondrial Cytochrome c release was analyzed (staining with DAPI (Sigma) and anti-Cytochrome c antibody (BioLegend, San Diego, California, USA; Attune NxT Flow Cytometer). Release of cytochrome c was analyzed in percentage according to control treated cells.

CellTiterGlo Staining

Cells were incubated with indicated drugs for 24 h in a total volume of 50 µl per well in an opaque 384 well plate. After incubation, treated cells were equilibrated to room temperature and 50 µl of CellTiterGlo® reagent was added to each well. Contents were mixed on an orbital shaker for 2min and incubated for 10min at room temperature. Luminescence was recorded with a Tecan plate reader and signals were normalized to control wells.

qRT-PCR Analysis

Total RNA was isolated from PDX cells (Quick-RNA Miniprep Kit, Zymo Research) and 500 ng total RNA was transcribed using SuperScript™ II Reverse Transkriptase (ThermoFisher). qRT-PCRs were performed using SsoAdvanced Universal SYBR Green Supermix (BioRad) using a Bio-Rad CFX Connect Real-Time PCR Detection System with the following protocol: 95 °C for 30 sec, then 40 cycles of 95 °C for 5 sec followed by different melting temperatures as indicated for each primer pair for 30 sec. The following human primer pairs were used: *TFAM* (3´-GTGGTTTTCATCTGTCTTGGCAAG-5´ and 5´-TTCCCTCCAACGCTGGGCAATT-3´) 61 °C, *OPA1* (3´-GCTCTGGAATAAAAGGAAGTTTACCA-5´ and 5´-GCGAAGTTTTAAGAGTCTCGTAGC-3´)[3] 65 °C, *MFN1* (3´-GGTGAATGAGCGGCTTTCCAAG-5´ and 5´-TCCTCCACCAAGAAATGCAGGC-3´)

61 °C, *MFN2* (3´-ATTGCAGAGGCGGTTCGACTCA-5´ and 5´-TTCAGTCGGTCTTGCCGCTCTT-3´) 65 °C, *MFF* (3´-GCTGTATTCACGTCTGAGCA-5´ and 5´-AGTTGGGTGGACTGATGAGG-3´) 64 °C, *DRP1* (3´- GATGCCATAGTTGAAGTGGTGAC-5´ and 5´-CCACAAGCATCAGCAAAGTCTGG-3´) 64 °C and *B2M* (3´-GTGGAGCATTCAGACTTGTCTTTCAGCAAGGAC-5´ and 5´-CACTTAACTATCTTGGCCTGTGACAAAGTCACATGG-3´) 65 °C was used. All primer sequences, except OPA1 were derived from OriGene Technologies, INC. (Rockville, Maryland, USA). Expression values of fusion and fission factors were normalized to *B2M* and log-transformed and $2^{(-\Delta\Delta)CT}$ values are shown.

Statistical Analysis

Statistical analyses were performed with GraphPad Prism software (version 9). Data obtained from replicate analyses with numbers of biological and/or technical replicates as indicated in the corresponding figure legends were analyzed by two-sided T-test assuming equal variances, Spearman correlation or Chi-square test as indicated. Combination effects and synergy scores were analyzed (Synergyfinder, https://synergyfinder.fimm.fi/synergy/20240318114830462623/) using the Bliss independence model.

Supplementary Figure Legends:

Supplementary Figure 1: Estimation of EC_{50} values after VEN, Daunorubicin and Staurosporine treatment in RS4;11 VEN resistant lines

(A) Starting from the BCP-ALL cell line RS4;11, five parallel VEN insensitive lines were generated by exposure to increasing concentrations of VEN over time (49 passages and 8 months of continuous treatment). In the beginning, RS4;11 lines were exposed to low VEN concentrations of 1 nM and cell viability was regularly determined by FSC/SCC (FACS measurement). As soon as all five lines showed viability over 90 %, VEN concentrations were increased and indicated along with the respective time to recovery (> 90% viability). **(B)** Comparison of EC_{50} values (after VEN treatment) in RS4;11 VEN^{sens} line 1 and VEN^{ins} line 1-5 after cultivation with increasing VEN concentrations (ranging from 4 nM up to 100 nM). **(C)** RS4;11 VEN^{sens} line 1 – 5 and VEN^{ins} line 1 – 5 were treated with increasing concentrations of Daunorubicin and Staurosporine and EC_{50} values were estimated. **(D)** RS4;11 VEN^{sens} line 1 and VEN^{ins} line 1 – 5 were cultivated for 20 weeks under drug holiday conditions and EC_{50} values were estimated after VEN treatment for 72 hours. **(E)** Example of cell death analysis and gating applied according to forward and side scatter criteria (flow cytometry) in ALL cells: VEN^{sens} line 1 was exposed to DMSO or to 10 nM and 25 µM VEN for 72 h showing increasing cell death with increasing VEN concentrations.

Supplementary Figure 2: BAX and BAK expression in VEN^{sens}/VEN^{ins} BCP-ALL cells and RS4;11 VEN^{sens} and VEN^{ins} lines and PDX samples

(A) Western Blot analysis of BAK and BAX protein levels in five RS4;11 VEN^{sens} lines and five VEN^{ins} lines. **(B)** Western Blot analysis of BAK and BAX protein levels in

VEN^{sens} cell lines RS4;11, MHH-CALL-2, KOPN-8 and EU-3 compared to VEN^{ins} cell lines RCH-ACV and NALM-6 (one representative blot of three independent experiments). **(C)** Western Blot analysis of three VEN^{sens} and three VEN^{ins} PDX samples. BAK and BAX protein expression levels are shown (densitometric analysis relative to geometric mean of all samples, normalized to GAPDH loading control).

Supplementary Figure 3: MCL-1 protein expression upon withdrawal of VEN in VEN^{ins} lines

(A) Western Blot analysis of MCL-1 protein expression in RS4;11 VEN^{ins} line 1. Cells were analyzed after VEN removal for indicated time points (N=1). MCL-1 protein levels (densitometric analysis relative to 72 h/100 nM VEN exposure and normalized to β-actin loading control) are shown. **(B)** Western Blot analysis of RS4;11 VEN^{sens} line 1 and VEN^{ins} lines 1-5 exposed to VEN or 96 hours after VEN removal (drug holiday) (N=1). MCL-1 protein expression levels are shown (densitometric analysis relative to VEN^{sens} line 1 after 72 h exposure to solvent control, normalized to β-actin loading control). Unpaired two-tailed Student’s T-test was used to calculate p-values.

Supplementary Figure 4: RNA-Seq and GSEA analysis comparing RS4;11 VEN^{sens} and VEN^{ins} lines 1 - 5

Significantly enriched Reactome pathway gene sets in RS4;11 VEN^{ins} lines 1 - 5 compared to VEN^{sens} lines 1 – 5 are ordered due to the number of analyzed observed genes overlapping with corresponding Reactome gene sets. Upregulated and downregulated pathways (in VEN^{ins}) are shown in green and red, respectively. Additionally, the top 20 up- and down-regulated genes are shown.

Supplementary Figure 5: Accompanied analysis of cell death induction, loss of Mitochondrial outer membrane potential and cytochrome *c* release after acute VEN treatment and metabolic profiling – VEN^{sens} and VEN^{ins} lines

RS4;11 VEN^{sens} lines 1 – 5 and VEN^{ins} lines 1 – 5 were treated for 3 hours with DMSO, 10 nM or 100 nM VEN. Cell death induction was analyzed by FSC/SSC criteria (N=1). MMP loss (TMRM staining) and cytochrome *c* release (intracellular cytochrome c staining of fixed cells) were estimated and median fluorescence intensities (MFI) were determined and normalized to MFIs of unstained controls (N=1, in triplicates, ± standard deviation).

Supplementary Figure 6: Characteristics of BCP-ALL cell lines

Association of BCP-ALL cell line characteristics with their sensitivity towards VEN sensitivity (n.k.; not known).

Supplementary Figure 7: Accompanied analysis of cell death induction, loss of Mitochondrial outer membrane potential and cytochrome *c* release after acute VEN treatment and metabolic profiling – BCP-ALL cell lines

BCP-ALL cell lines were treated for 3 hours with DMSO, 10 nM, 100 nM VEN and cell death induction was analyzed by FSC/SSC criteria (N=1). MMP loss (TMRM staining) and cytochrome *c* release (intracellular cytochrome c staining of fixed cells) were estimated and median fluorescence intensities (MFI) were determined and normalized to MFIs of unstained controls (N=1, in triplicates, ± standard deviation).

Supplementary Figure 8: Intrinsic VEN resistant BCP-ALL cell line and patient derived xenograft samples are characterized by increased metabolic activity

(A) Metabolic profiles of two BCP-ALL cell lines (KOPN-8 and EU-3) after 3 hours exposure to DMSO or 10 nM and 100 nM VEN (N=1 in five technical replicates, ± standard error of mean). **(B)** N=31 PDX samples were exposed to increasing concentrations (1 nM, 5 nM, 10 nM, 50 nM, 100 nM, 250 nM, 500 nM; 1 µM, 5 µM and 10 µM) of VEN for 24 hours. Relative cell death rates were assessed by propidium iodide staining and normalized to DMSO controls (N=1, in triplicates). **(C)** Basal and ATP-linked respiration of three VEN^{sens} (green) and three VEN^{ins} (red) PDX samples (± standard error of mean) and correlation analysis of basal and ATP-linked respiration to corresponding VEN EC_{50} values is shown (Spearman correlation; r, correlation coefficient; p, significance).

Supplementary Figure 9: Characteristics of PDX-samples

Association of PDX sample characteristics with their sensitivity towards VEN sensitivity.

Supplementary Figure 10: Estimation of VEN EC_{50} values in cell lines and PDX samples

BCP-ALL cell lines and PDX samples were exposed to increasing VEN concentrations for 72 or 24 hours, respectively, and EC_{50} values were estimated.

Supplementary Figure 11: Accompanied analysis of cell death induction, loss of Mitochondrial outer membrane potential and cytochrome *c* release after acute VEN treatment and metabolic profiling – PDX samples

PDX samples were treated for 3 hours with DMSO, 500 nM, 1 000 nM or 2 500 nM of VEN and cell death induction was analyzed by FSC/SSC criteria (N=1). MMP loss (TMRM staining) and cytochrome *c* release (intracellular cytochrome c staining of

fixed cells) were estimated and median fluorescence intensities (MFI) were determined and normalized to MFIs of unstained controls (N=1, in triplicates, ± standard deviation).

Supplementary Figure 12: Analysis of mitochondrial mass and outer membrane potential in PDX samples

(A) To determine the number of mitochondria, electron microscopy images of VEN sensitive and VEN resistant PDX samples were counted (N=3+3 biological replicates, 30 cells each). Expression levels of *TFAM* RNA was analyzed relative to *B2M* expression in sensitive compared to highly resistant PDX samples (N=30) (> 1 µM EC_{50}), Unpaired two-tailed Student's T-test was used to calculate p-values. Three VEN sensitive and resistant PDX samples were stained with MitoTracker or TMRM to assess mitochondrial mass and mitochondrial outer membrane potential. Median fluorescence intensities (MFI) were determined and normalized to MFIs of unstained controls (N=1, in triplicates, ± standard deviation). **(B)** *OPA1*, *MFN1*, *MFN2*, *DRP1* and *MFF* RNA expression was analyzed relative to *B2M* expression in sensitive compared to highly resistant PDX samples (> 1µM EC_{50}) (N=30; for analysis of *MFF* N=28). Unpaired two-tailed Student's T-test was used to calculate p-values (± standard deviation).

Supplementary Figure 13: Staurosporine sensitivity in PDX samples

Eight PDX samples with VEN EC_{50} values ranging from 54 to 9 526 nM were exposed to DMSO or increasing concentrations of Staurosporine (1 nM, 10 nM, 50 nM, 100 nM, 500 nM) for 24 h and cell death induction was analyzed using the CellTiterGlo assay. The Luminescence readout, which is directly proportional to the

number of metabolically active/alive cells, was normalized to control treatment. Curves are represented as 100 - relative activity (N=1 in triplicates).

Supplementary Figure 14: Similar sensitivities in RS4;11 VEN^{sens} and VEN^{ins} lines to combinations of Oligomycin along with the MCL-1 inhibitor S63845, Staurosporine, Daunorubicin, Vincristine+Dexamethasone+Asparaginase (VDA)

Interaction landscapes of dose-response matrix analyses are shown. Synergies (δ-scores) were calculated using synergyfinder. Synergistic effects are shown in red, additive effects in white and antagonistic effects in green. Bliss synergy scores indicate the average synergy score across the dose-response matrix. **(A)** RS4;11 VEN^{sens} and VEN^{ins} line 1-5 were incubated with DMSO, increasing concentrations of S63845 (10 nM, 100 nM, 250 nM, 500 nM, 1 000 nM), or Oligomycin (5 nM, 50 nM, 100 nM, 500 nM and 5 000 nM), or titrated in an one to one matrix combination. **(B)** RS4;11 VEN^{sens} and VEN^{ins} line 1-5 were incubated with DMSO, increasing concentrations of Staurosporine (1 nM, 5 nM, 10 nM, 25 nM and 50 nM), or Oligomycin (concentrations as in A) or titrated in an one to one matrix combination. **(C)** RS4;11 VEN^{sens} and VEN^{ins} line 1-5 were incubated with DMSO, increasing concentrations of Daunorubicin (1 nM, 5 nM, 10 nM, 25 nM, 50 nM), or Oligomycin (concentrations as in A), or titrated in an one to one matrix combination. **(D)** RS4;11 VEN^{sens} and VEN^{ins} line 1-5 were incubated with DMSO, increasing concentrations of the combination of Vincristine/Dexamethasone/Asparaginase (0.00025 ng/ml / 0.00125 ng/ml / 0.000125 U/ml; 0.0005 ng/ml / 0.0025 ng/ml / 0.00025 U/ml; 0.005 ng/ml / 0.005 ng/ml / 0.0005 U/ml; 0.25 ng/ml / 2.5 ng/ml / 0.25 U/ml and 0.5 ng/ml / 5 ng/ml / 0.5 U/ml), or Oligomycin (concentrations as in A), or titrated in an one to one matrix combination. Analysis of cell death after 72 h according to FSC/SSC criteria

(flow cytometry) is shown. Comparison of Bliss scores of RS4;11 VEN^{sens} and VEN^{ins} lines 1-5. Unpaired two-tailed Student's T-test (± standard deviation); ns, not significant.

Supplementary Figure 15: Similar sensitivities of VEN-sensitive and VEN-insensitive BCP-ALL cell lines to combinations of Oligomycin along with the MCL-1 inhibitor S63845, Staurosporine, Daunorubicin, Vincristine/Dexamethasone/Asparaginase (VDA)

Interaction landscapes of dose-response matrix analyses are shown. Synergies (δ-scores) were calculated using synergyfinder. Synergistic effects are shown in red, additive effects in white and antagonistic effects in green. Bliss synergy scores indicate the average synergy score across the dose-response matrix.

VEN-sensitive (RS4;11 and EU-3) and VEN-insensitive (RCH-ACV and NALM-6) BCP-ALL cell lines were either incubated with DMSO, increasing concentrations of S63845 (10 nM, 100 nM, 250 nM, 500 nM, 1 000 nM), Staurosporine (1 nM, 5 nM, 10 nM, 25 nM and 50 nM), Daunorubicin (1 nM, 5 nM, 10 nM, 25 nM, 50 nM) or the combination of Vincristine/Dexamethasone/Asparaginase (0.00025 ng/ml / 0.00125 ng/ml / 0.000125 U/ml; 0.0005 ng/ml / 0.0025 ng/ml / 0.00025 U/ml; 0.005 ng/ml / 0.005 ng/ml / 0.0005 U/ml; 0.25 ng/ml / 2.5 ng/ml / 0.25 U/ml and 0.5 ng/ml / 5 ng/ml / 0.5 U/ml) and were combined with increasing concentrations of Oligomycin (5 nM, 50 nM, 100 nM, 500 nM and 5 000 nM) in an one to one matrix combination. Analysis of cell death after 72 h according to FSC/SSC criteria (flow cytometry) is shown. Comparison of Bliss scores of RS4;11 VEN^{sens} and VEN^{ins} lines 1-5, unpaired two-tailed Student's T-test (± standard deviation); ns, not significant.

Supplementary Figure 16: Co-treatment with VEN and Oligomycin significantly induces cell death in VEN resistant RS4;11 cells and in BCP-ALL cell lines

(A) RS4;11 VEN^{sens} and VEN^{ins} cells, **(B)** RS4;11 VEN^{sens} and VEN^{ins}-dh lines, **(C)** three VEN^{sens} BCP-ALL cell lines and **(D)** VEN^{ins} BCP-ALL cell lines were treated with DMSO or increasing concentrations of Oligomycin (5 nM, 50 nM, 100 nM, 250 nM, 500 nM or 5 000 nM) for 72 hours and cell death was determined via FSC/SSC criteria (N=3, triplicates). *$p < 0.05$, **$p < 0.005$, ***$p < 0.001$, ****$p < 0.0001$, unpaired two-tailed Student’s T-test (± standard deviation).

References to Supplementary Methods:

1. Meyer, L. H.; Eckhoff, S. M.; Queudeville, M.; Kraus, J. M.; Giordan, M.; Stursberg, J.; Zangrando, A.; Vendramini, E.; Möricke, A.; Zimmermann, M.; Schrauder, A.; Lahr, G.; Holzmann, K.; Schrappe, M.; Basso, G.; Stahnke, K.; Kestler, H. A.; te Kronnie, G.; Debatin, K.-M. Early Relapse in ALL Is Identified by Time to Leukemia in NOD/SCID Mice and Is Characterized by a Gene Signature Involving Survival Pathways. *Cancer Cell* **2011**, *19* (2), 206–217. https://doi.org/10.1016/j.ccr.2010.11.014.

2. Boldrin, E.; Gaffo, E.; Niedermayer, A.; Boer, J. M.; Zimmermann, M.; Weichenhan, D.; Claus, R.; Münch, V.; Sun, Q.; Enzenmüller, S.; Seyfried, F.; Demir, S.; Zinngrebe, J.; Cario, G.; Schrappe, M.; Den Boer, M. L.; Plass, C.; Debatin, K.-M.; te Kronnie, G.; Bortoluzzi, S.; Meyer, L. H. MicroRNA-497/195 Is Tumor Suppressive and Cooperates with CDKN2A/B in Pediatric Acute Lymphoblastic Leukemia. *Blood* **2021**, *138* (20), 1953–1965. https://doi.org/10.1182/blood.2020007591.

3. Sciezynska A, Ruszkowska E, Szulborski K, Rydz K, Wierzbowska J, Kosinska J, Rekas M, Ploski R, Szaflik P, Oldak M, Processing of OPA1 with a novel N-terminal mutation in patients with autosomal dominant optic atrophy: Escape from nonsense mediated decay, PlosOne 2017, 12(8): e0183866. https://doi.org/10.1371.
