# Supplementary material for: Venetoclax resistance in acute lymphoblastic leukemia is characterized by increased mitochondrial activity and can be overcome by co-targeting oxidative phosphorylation
Source: Cell Death Dis. 2024 Jul 3;15(7):475. doi: 10.1038/s41419-024-06864-7 (PMC11222427; doi:10.1038/s41419-024-06864-7)

Suppl. Figure 1

A

**VEN concentration**	**1nM**	**4nM**	**6nM**	**12nM**	**25nM**	**50nM**	**100nM**	
**recovery time (in weeks)**	2	4	8	5	5	4	4	**=32 weeks**

B

**Exposure concentration (VEN)**	**4nM**	**6nM**	**12nM**	**25nM**	**50nM**	**100nM**
VEN^{sens} line 1	2,35nM	3,3nM	17,4nM	2,8nM	1,6nM	6,4nM
VEN^{ins} line 1	26,6nM	263,1nM	1550nM	12420nM	13040nM	31880nM
VEN^{ins} line 2	24,7nM	188,3nM	1190nM	9420nM	12570nM	35220nM
VEN^{ins} line 3	39,4nM	251,2nM	1040nM	5340nM	11800nM	22540nM
VEN^{ins} line 4	27,2nM	240,1nM	950nM	5090nM	7410nM	15670nM
VEN^{ins} line 5	21,7nM	233,7nM	1350nM	5250nM	11650nM	25650nM

C

**RS4;11 VEN resistant development**	**Daunorubicin EC50 [nM]**
VEN^{sens} line 1	25
VEN^{sens} line 2	20
VEN^{sens} line 3	27
VEN^{sens} line 4	17
VEN^{sens} line 5	14
VEN^{ins} line 1	28
VEN^{ins} line 2	23
VEN^{ins} line 3	17
VEN^{ins} line 4	23
VEN^{ins} line 5	19

**RS4;11 VEN resistant development**	**Staurosporine EC50 [nM]**
VEN^{sens} line 1	54
VEN^{sens} line 2	53
VEN^{sens} line 3	55
VEN^{sens} line 4	45
VEN^{sens} line 5	46
VEN^{ins} line 1	121
VEN^{ins} line 2	94
VEN^{ins} line 3	76
VEN^{ins} line 4	64
VEN^{ins} line 5	45

D

**20 weeks drug holiday**	**Venetoclax EC50 [nM]**
VEN^{sens} line 1	14
VEN^{ins} line 1	8767
VEN^{ins} line 2	26015
VEN^{ins} line 3	22331
VEN^{ins} line 4	24332
VEN^{ins} line 5	23692

E

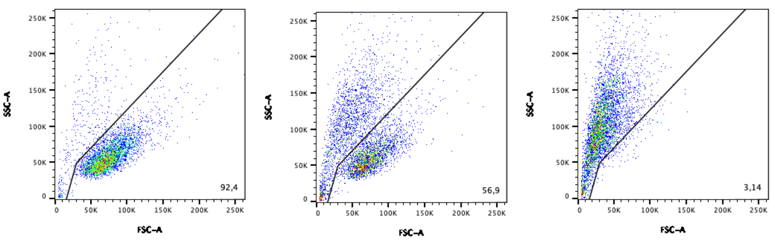

VEN^{sens} line 1 DMSO | VEN^{sens} line 1 10nM VEN | VEN^{sens} line 1 25μM VEN

Suppl. Figure 2

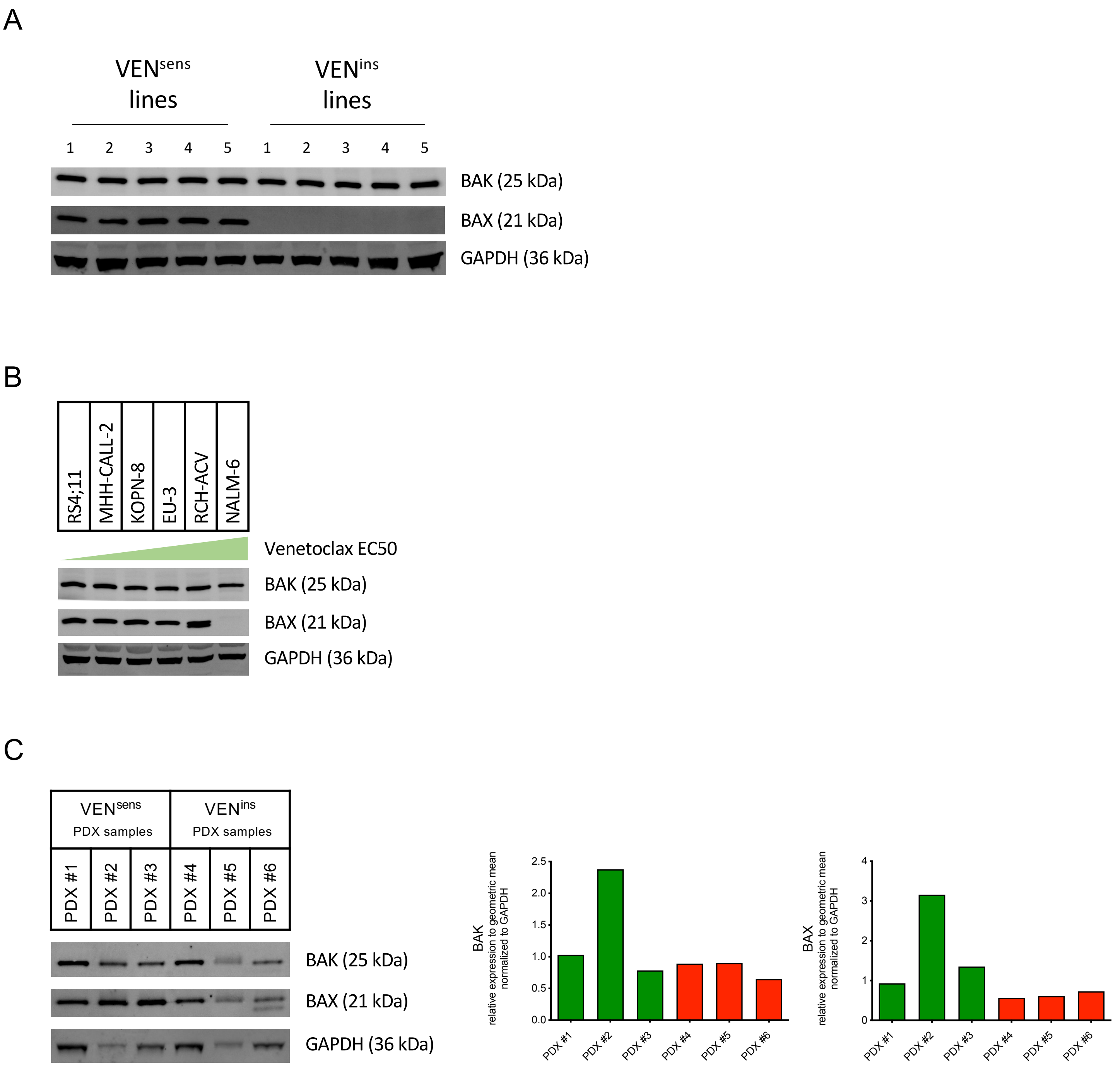

Suppl. Figure 3

A

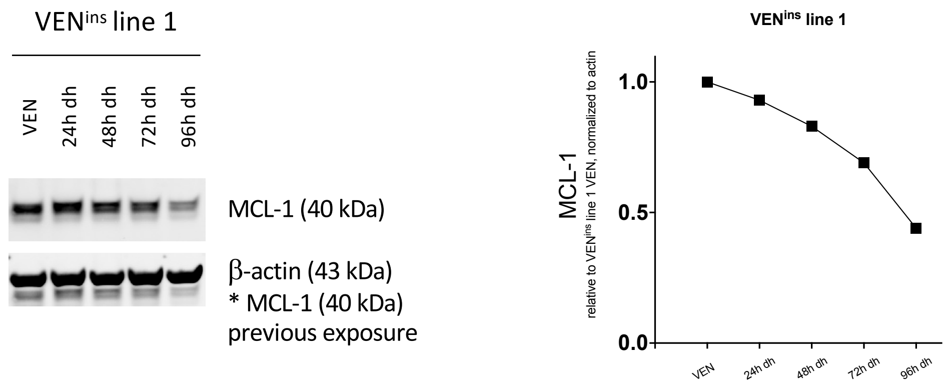

B

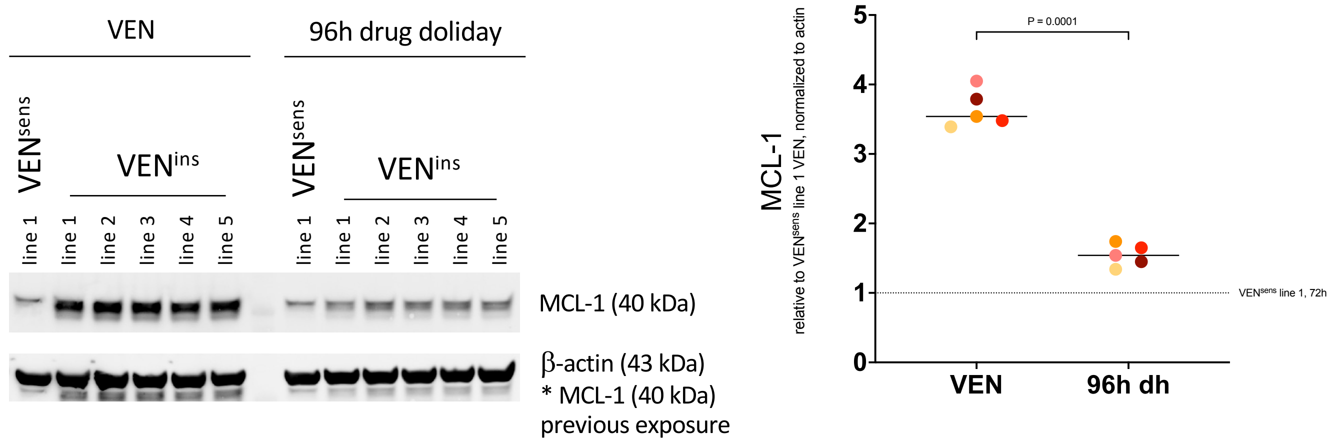

Suppl. Figure 4

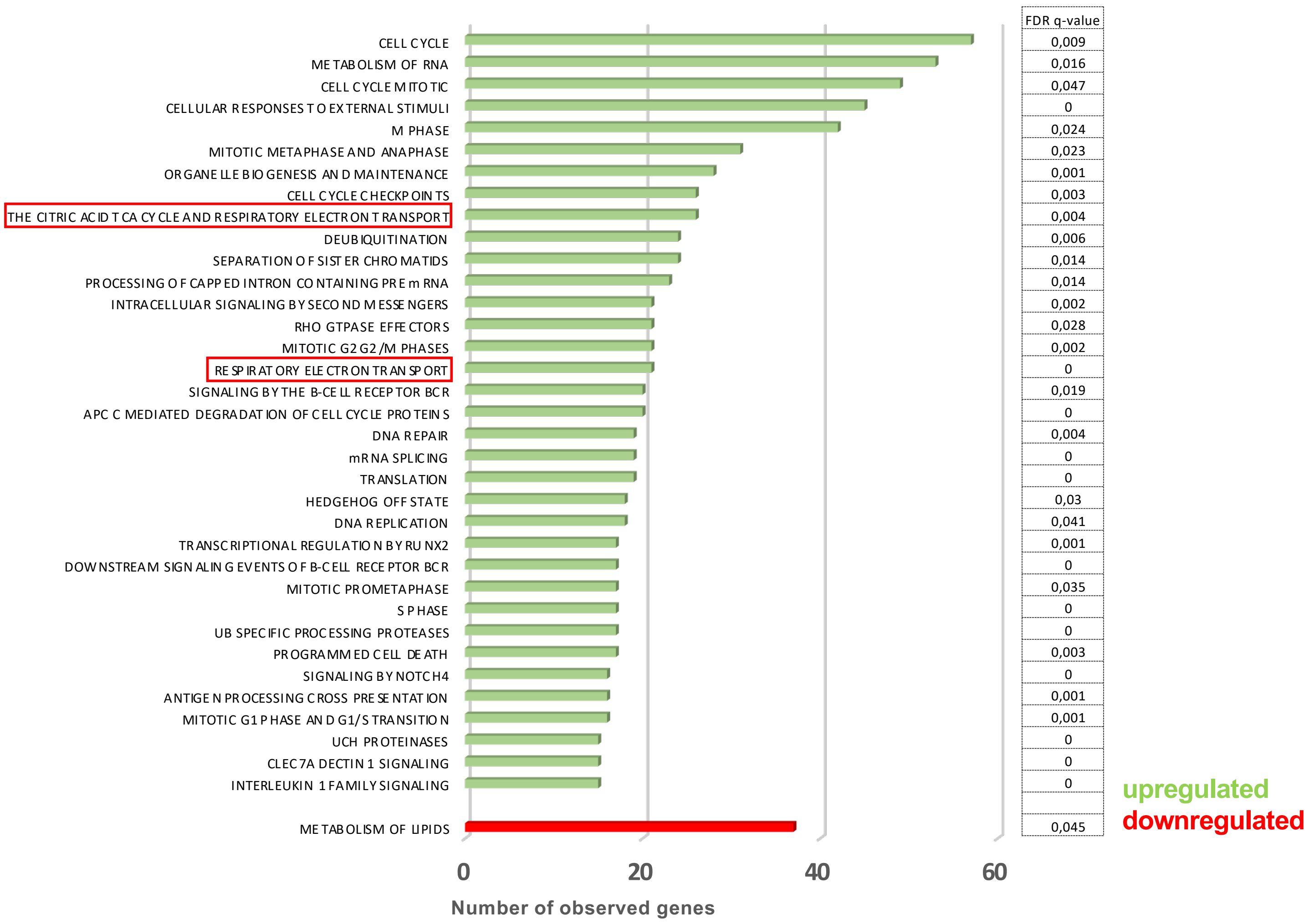

Top 20 significantly upregulated		Top 20 significantly downregulated	
AGPAT3	2,297	SERPINE1	-0,825
AC104849.1	1,155	CSPG4P13	-0,844
ID2	0,792	HLA-DQA1	-0,849
CBFA2T3	0,690	ADAM19	-0,852
AC017104.1	0,567	MTUS2	-0,882
SNAI3	0,541	AC113191.1	-0,895
AF064858.1	0,540	SNORD17	-0,904
UBE2CP4	0,517	GBP2	-0,915
CASC10	0,514	C1orf115	-0,953
TBC1D27P	0,497	ITGB5	-0,965
SORBS3	0,489	KDM5B	-0,980
AC021087.4	0,465	TBXA2R	-0,982
LINC00963	0,464	GPR82	-1,036
ETNK2	0,457	FRMPD1	-1,074
AC244502.1	0,453	HLA-DRB5	-1,089
TNFRSF13B	0,449	SPRY4	-1,097
HMX2	0,446	FRY	-1,204
MYO1C	0,423	TOX	-1,269
FKBP4	0,421	ANGPT1	-1,294
SKAP2	0,411	OCIAD2	-3,002

Suppl. Figure 5

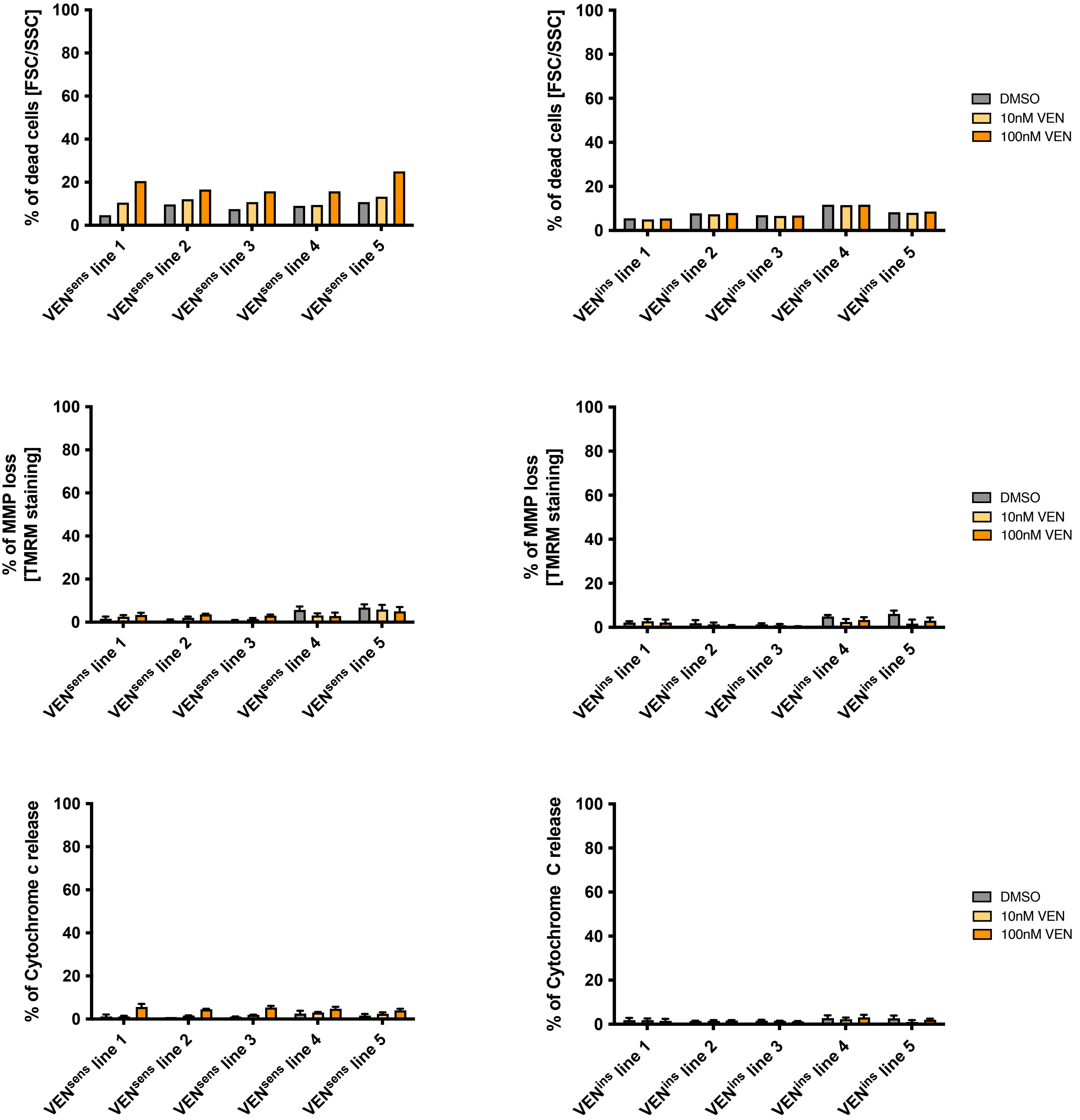

Suppl. Figure 6

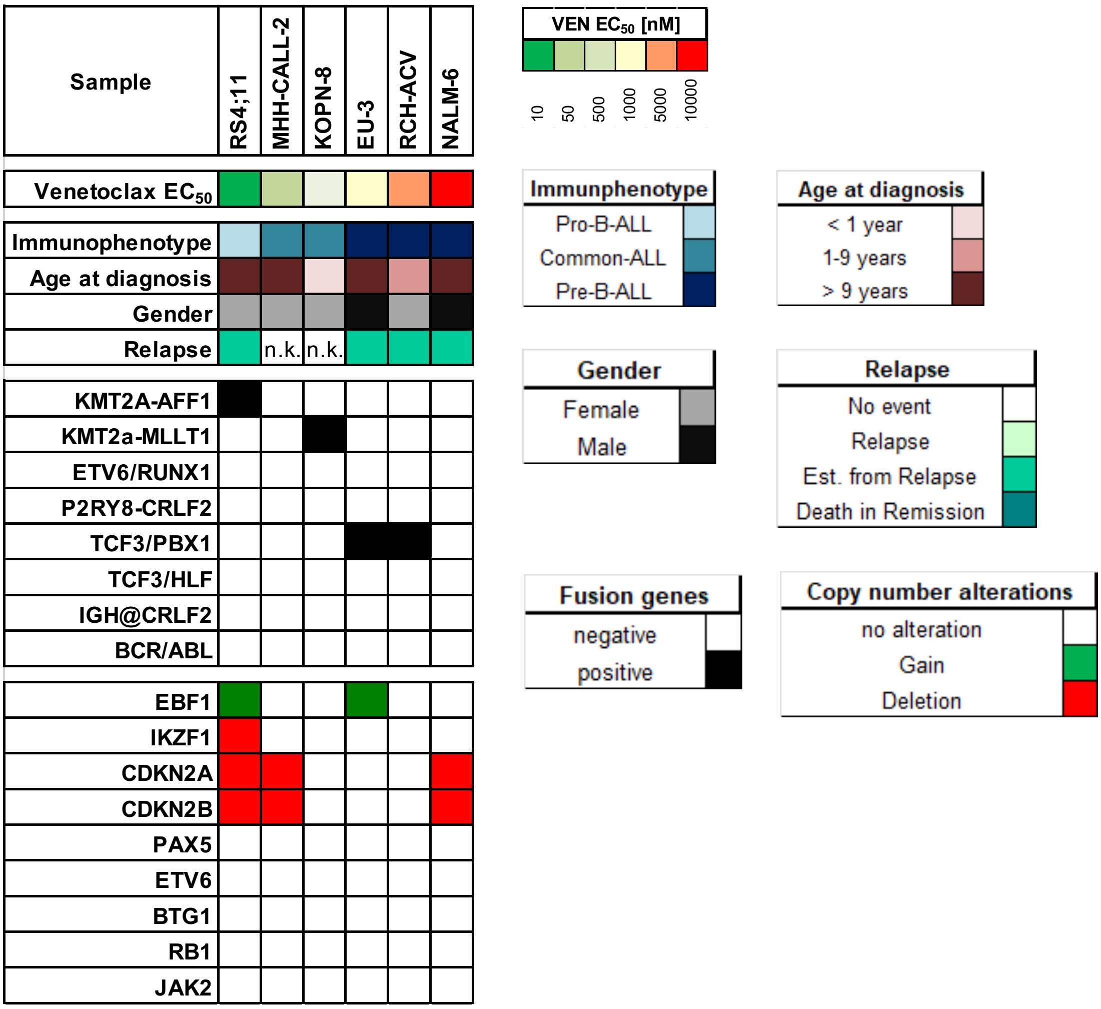

Suppl. Figure 7

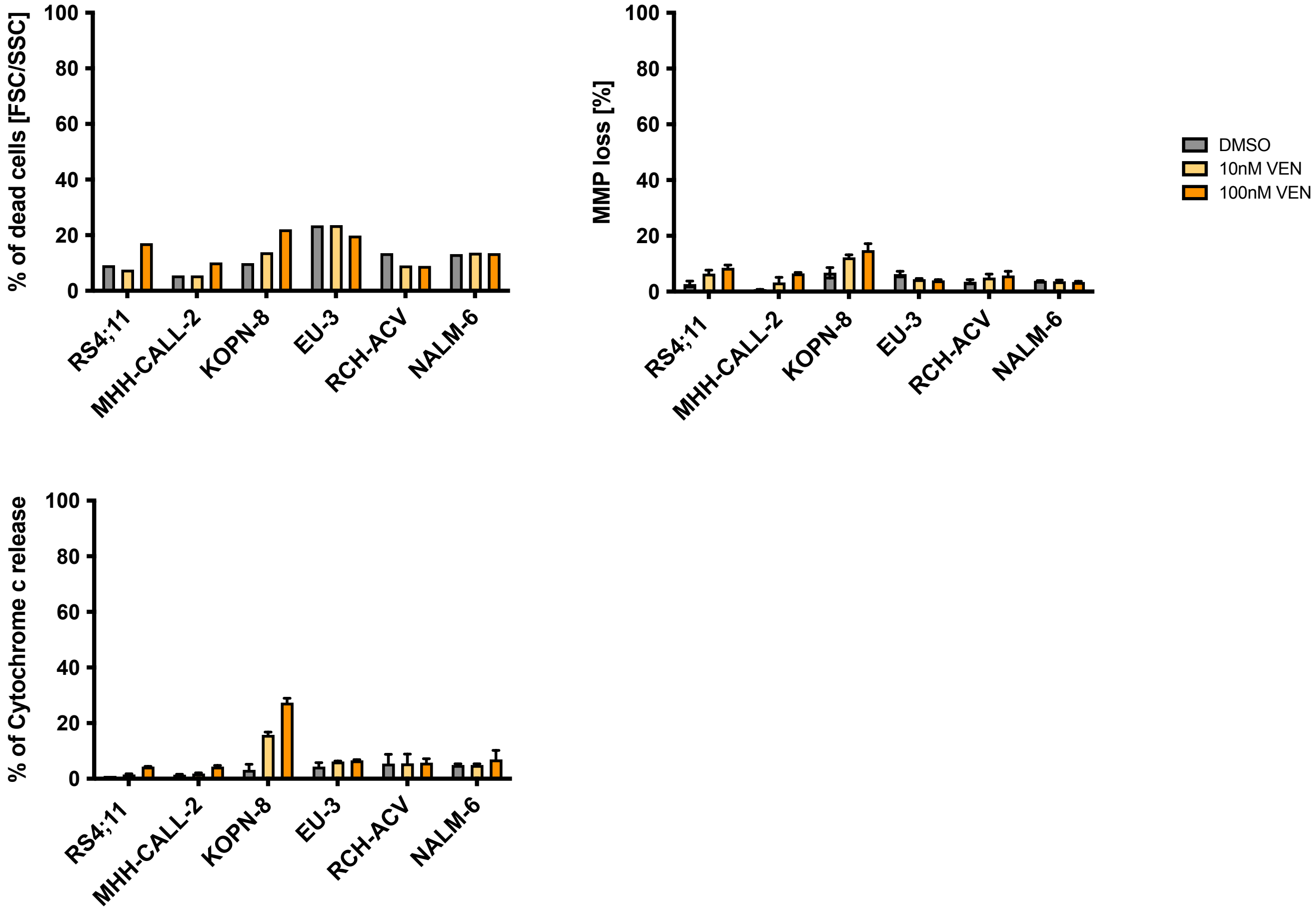

Suppl. Figure 8

A

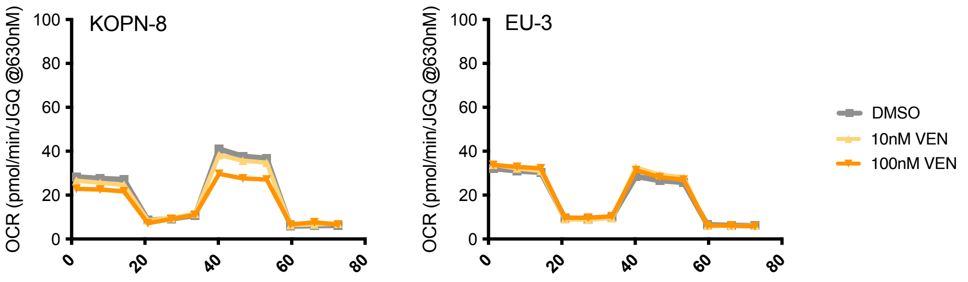

B

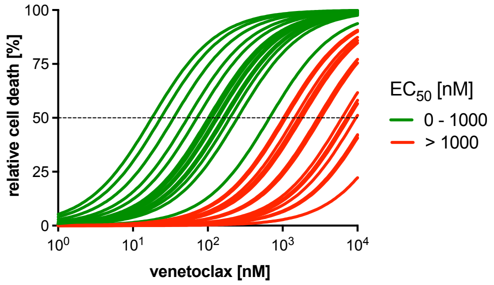

C

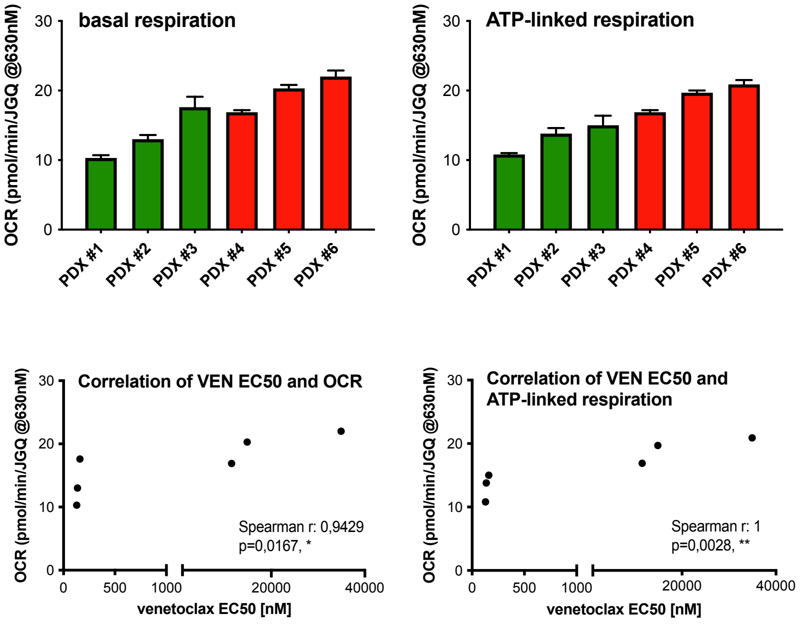

Suppl. Figure 9

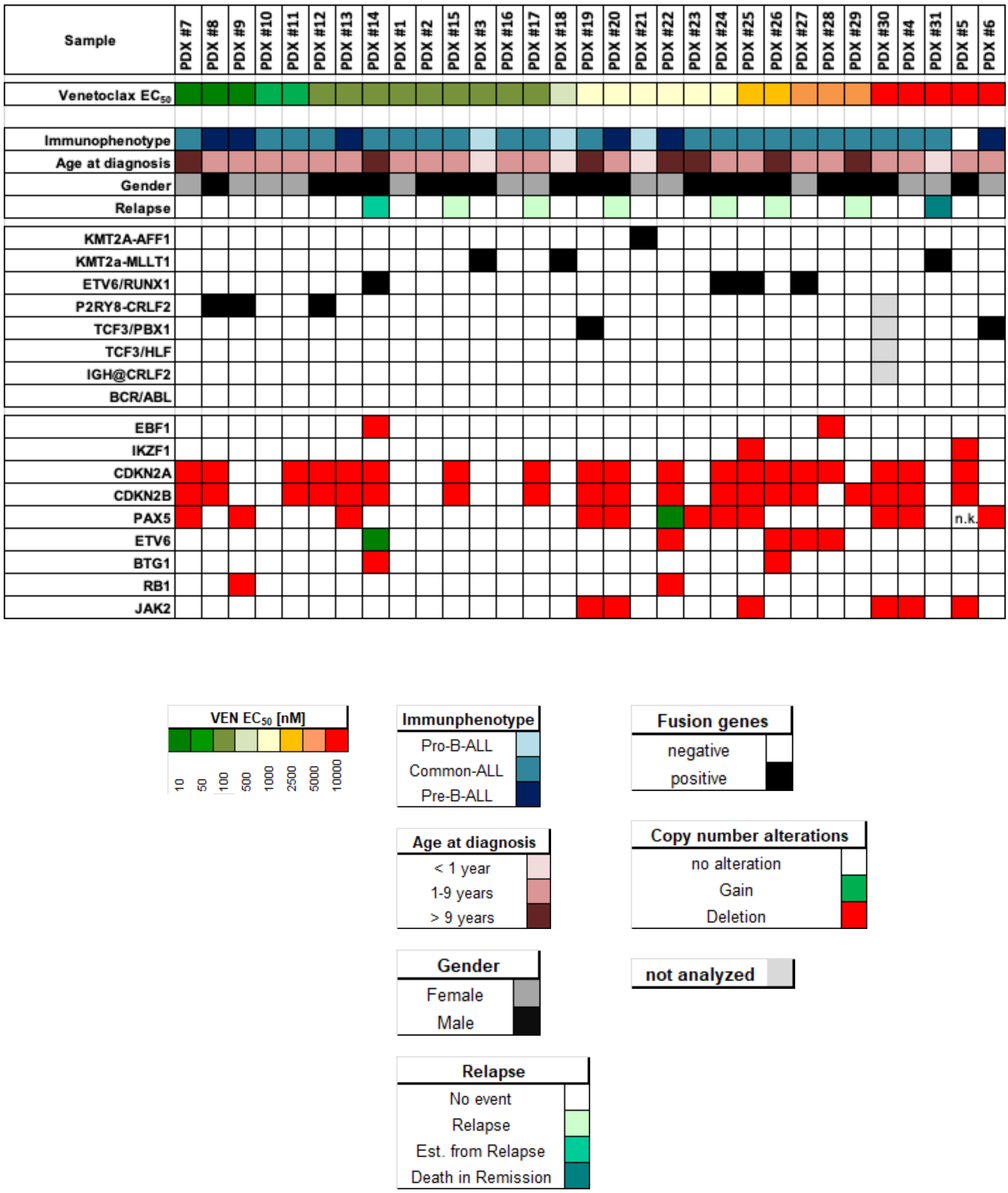

ID	Venetoclax EC50 [nM]
PDX #7	18
PDX#8	27
PDX #9	35
PDX #10	54
PDX #11	72
PDX #12	102
PDX #13	111
PDX #14	113
PDX #1	128
PDX #2	136
PDX #3	160
PDX #15	170
PDX #16	198
PDX #17	249
PDX #18	667
PDX #19	1028
PDX #20	1100
PDX #21	1201
PDX #22	1444
PDX #23	1637
PDX #24	1800
PDX #25	2967
PDX #26	3247
PDX #27	6209
PDX #28	7195
PDX #29	7677
PDX #30	9526
PDX #4	13694
PDX #31	13883
PDX #5	14851
PDX #6	34929

ID	Venetoclax EC50 [nM]
RS4;11	6
MHH-CALL-2	12
KOPN-8	415
EU-3	706
RCH-ACV	4630
NALM-6	12357

Suppl. Figure 11

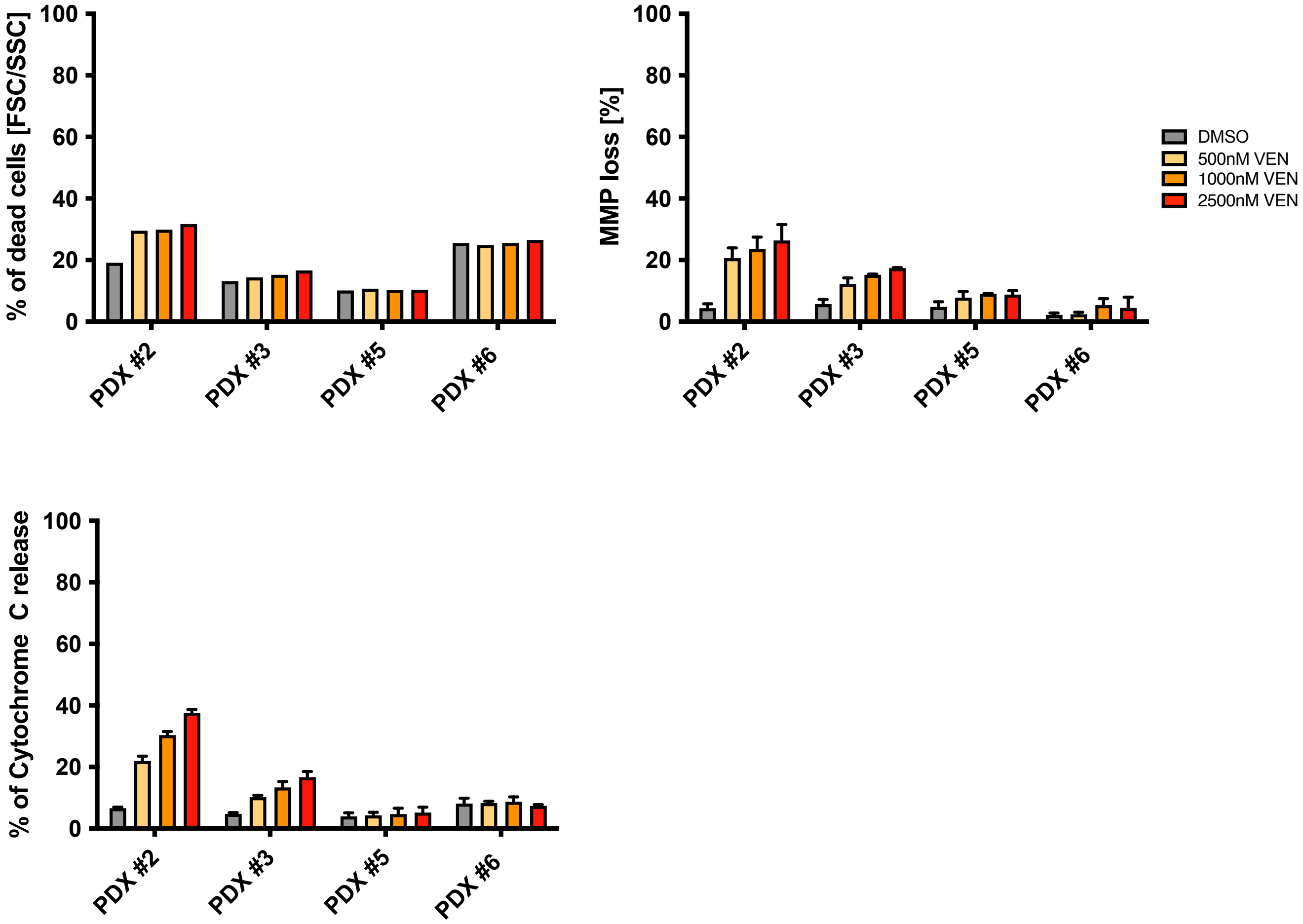

Suppl. Figure 12

A

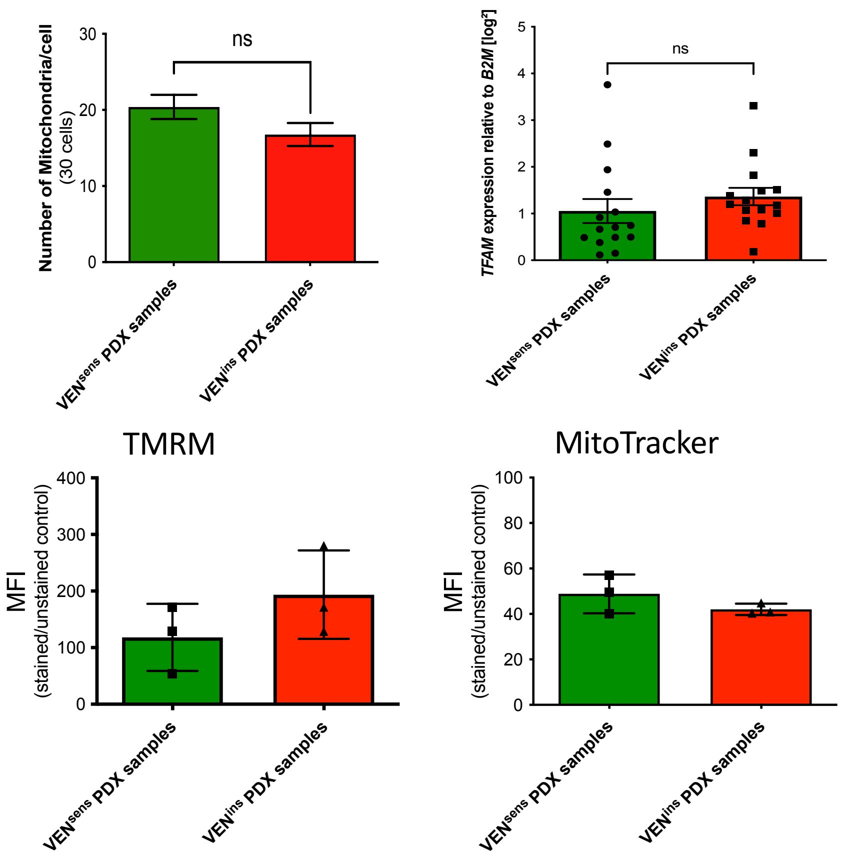

B

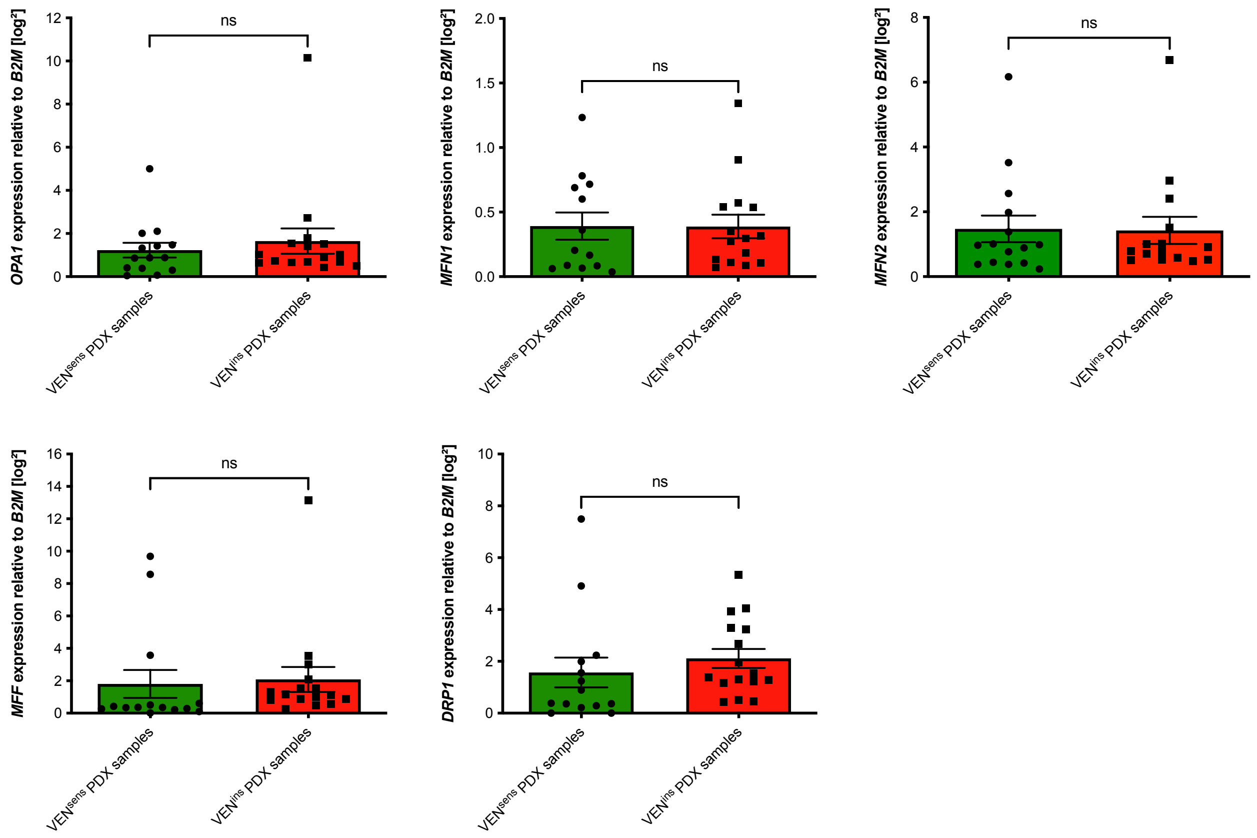

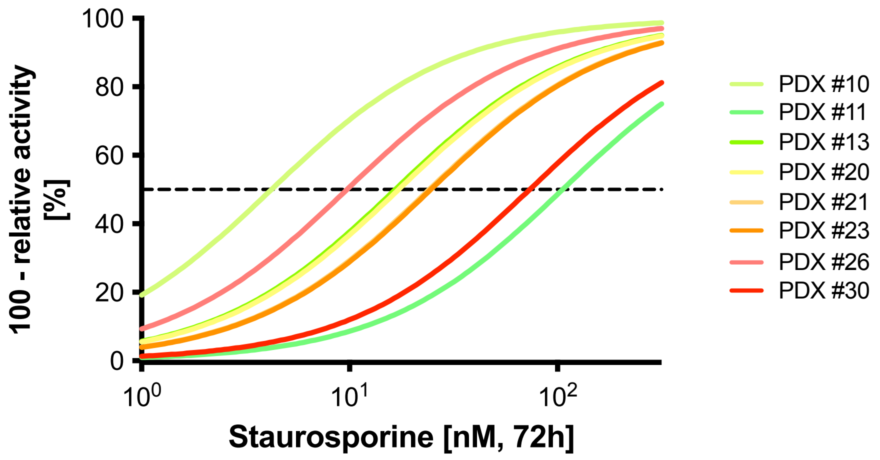

ID	VEN EC50 [nM]	Staurosporine EC50 [nM]
PDX #10	54	4
PDX #11	72	106
PDX #13	111	17
PDX #20	1100	17
PDX #21	1201	24
PDX #23	1637	25
PDX #26	3247	10
PDX #30	9526	73

Suppl. Figure 14

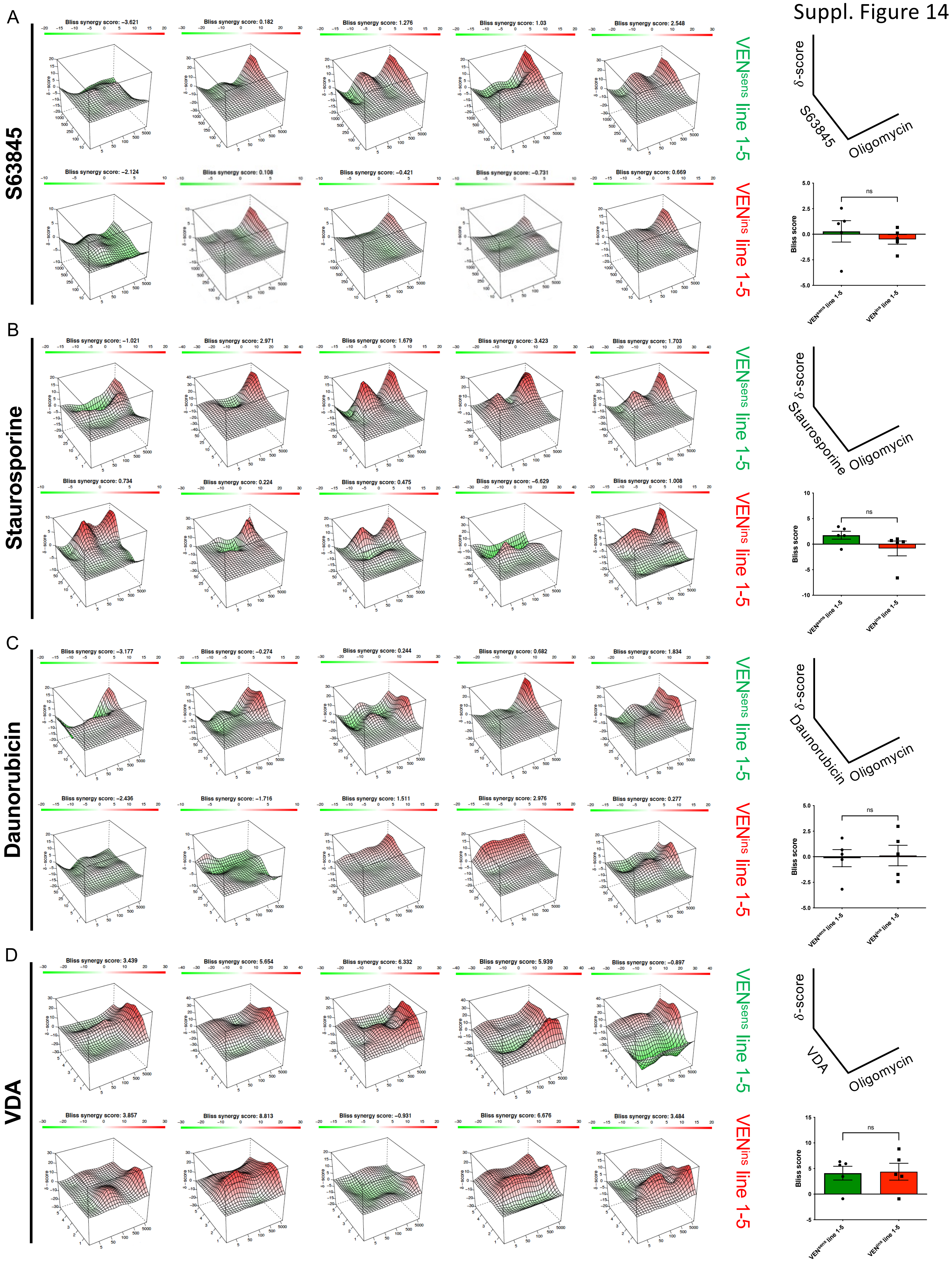

Suppl. Figure 15

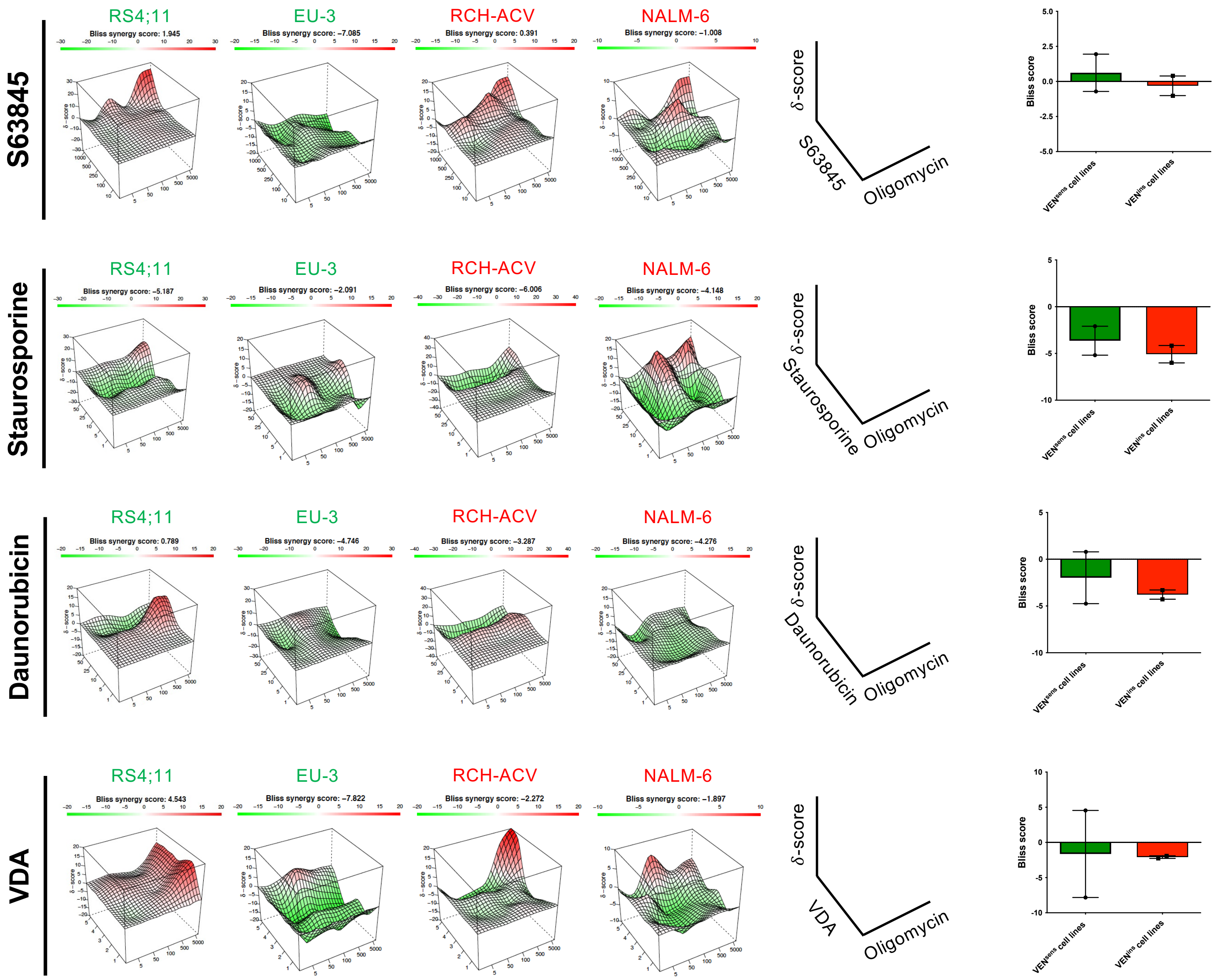

Suppl. Figure 16

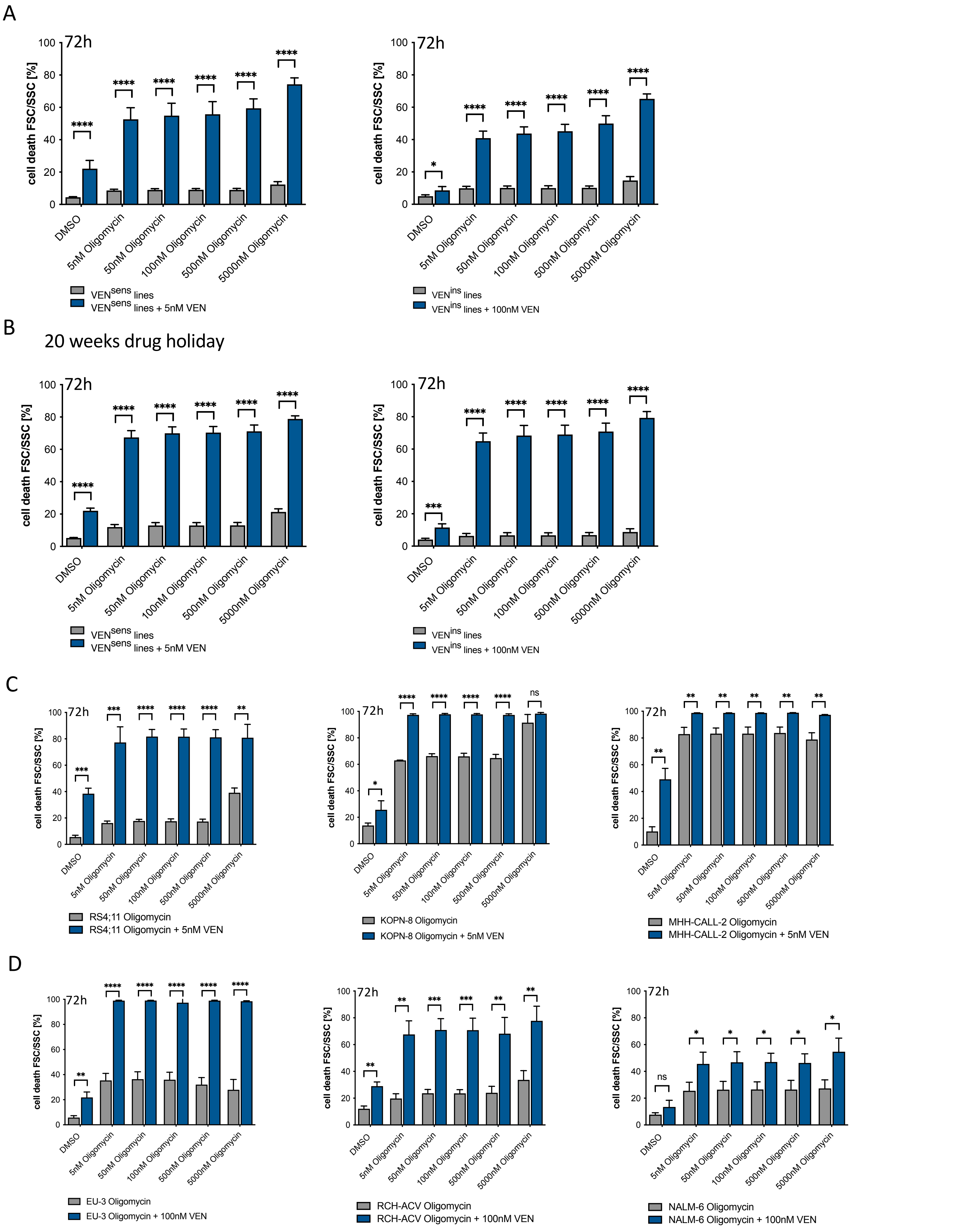

Supplement: Supplementary file 2 — Supplementary Figures [file 41419_2024_6864_MOESM2_ESM.pdf]
