# Supplementary material for: Venetoclax resistance in acute lymphoblastic leukemia is characterized by increased mitochondrial activity and can be overcome by co-targeting oxidative phosphorylation
Source: Cell Death Dis. 2024 Jul 3;15(7):475. doi: 10.1038/s41419-024-06864-7 (PMC11222427; doi:10.1038/s41419-024-06864-7)

Enzenmueller and Niedermayer *et al.*

Venetoclax Resistance in Acute Lymphoblastic Leukemia is characterized by Increased Mitochondrial Activity and Can Be Overcome by Co-targeting Oxidative Phosphorylation

Original data files to western blot analyses

Figure 2A

RS4;11 term
Whole cell lysate
Blot 1

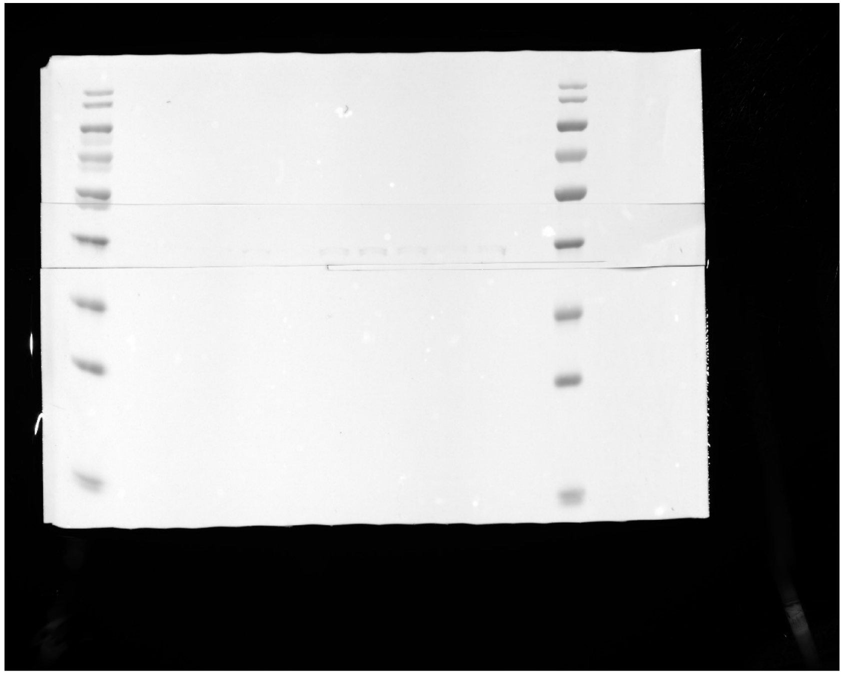

colorimetric

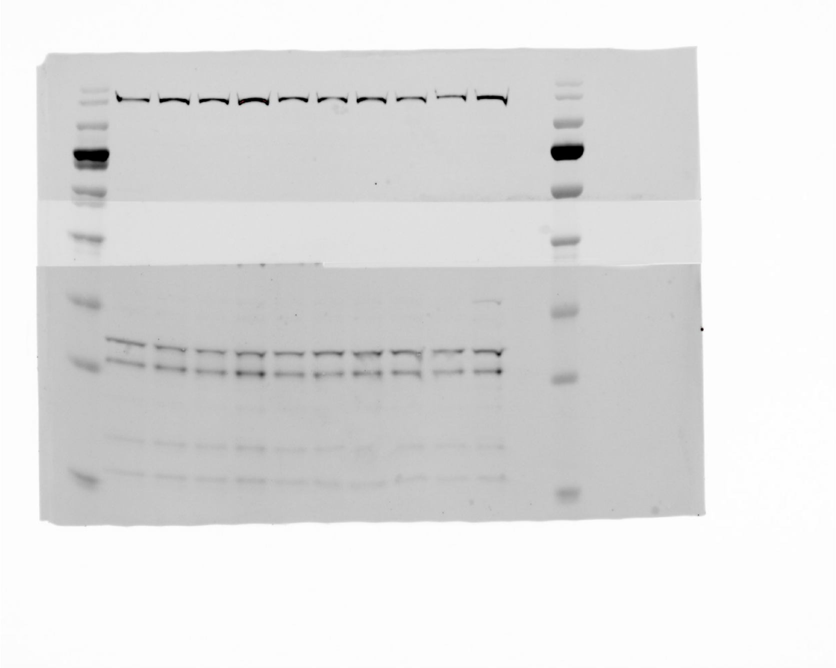

StarBright 700

RS4;11 term
Whole cell lysate
Blot 1

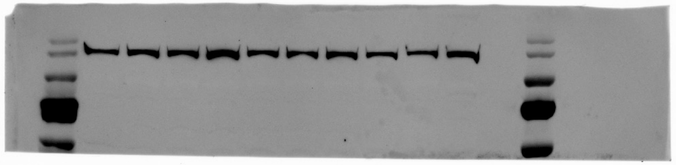

Vinculin

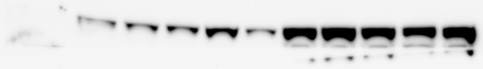

MCL-1

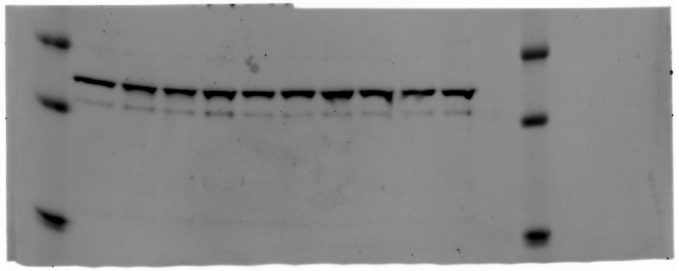

BCL-2

RS4;11 term
Whole cell lysate
Blot 2

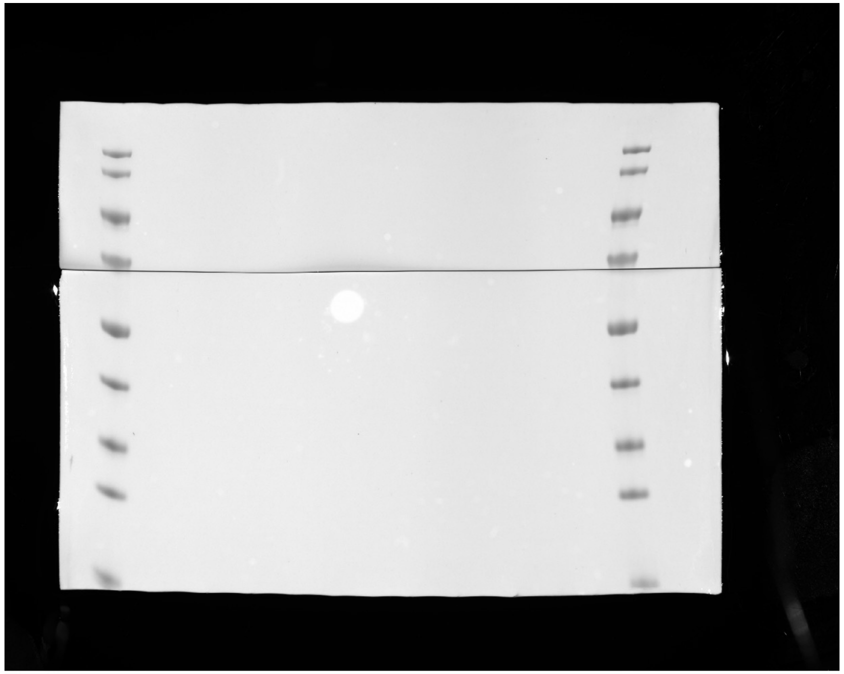

colorimetric

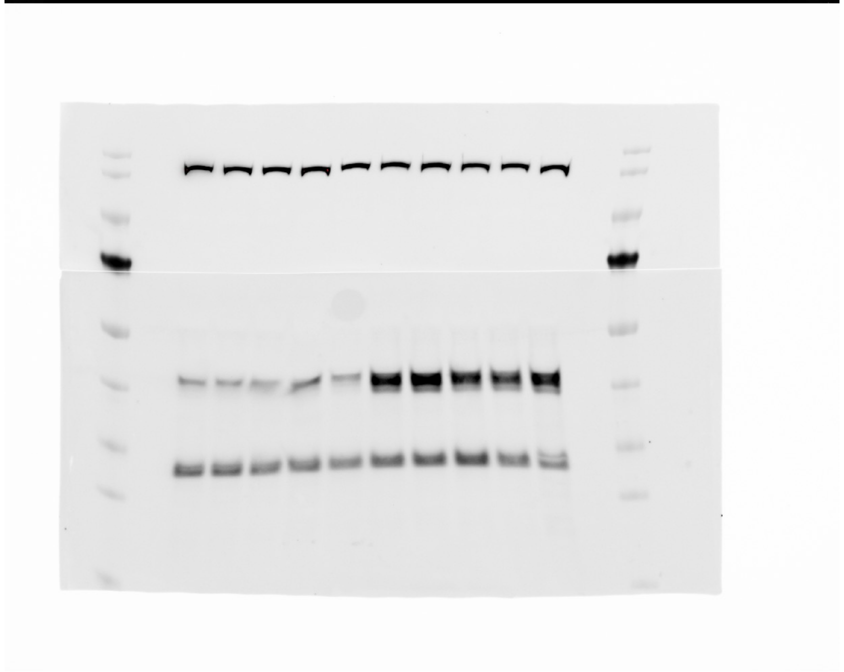

StarBright 700

RS4;11 term
Whole cell lysate
Blot 2

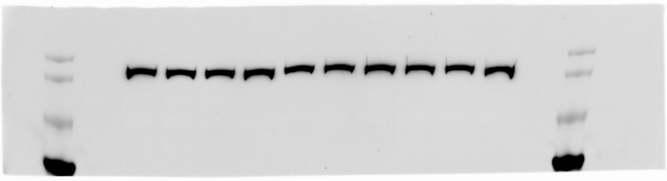

Vinculin

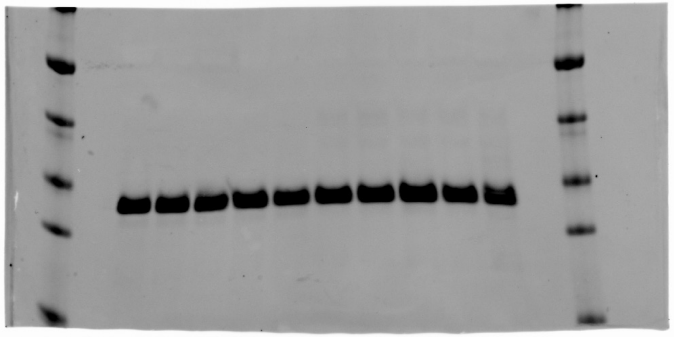

BCL-XL

Figure 2B

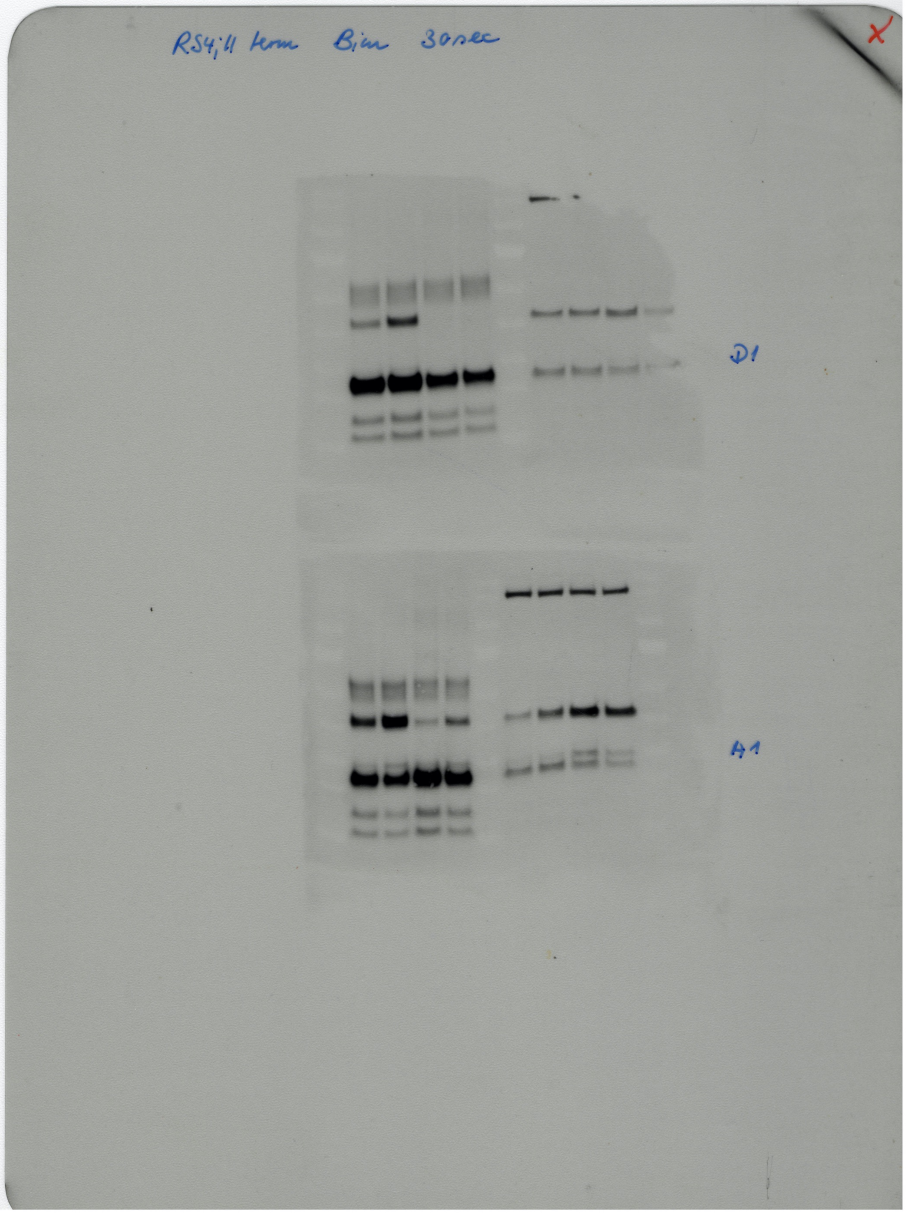
RS4;11 kom Bim 30sec
D1
A1

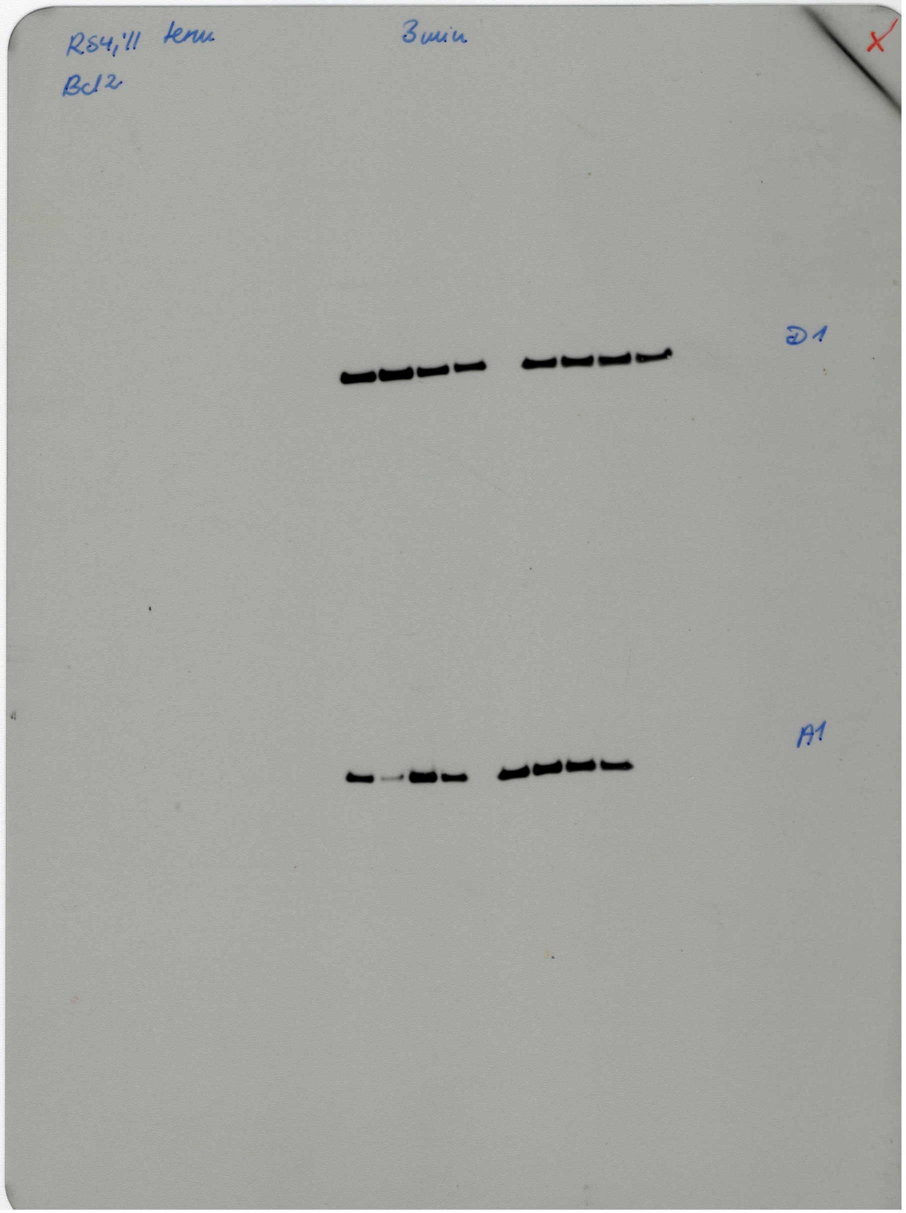
RS4;11
Bcl2
3min
D1
A1

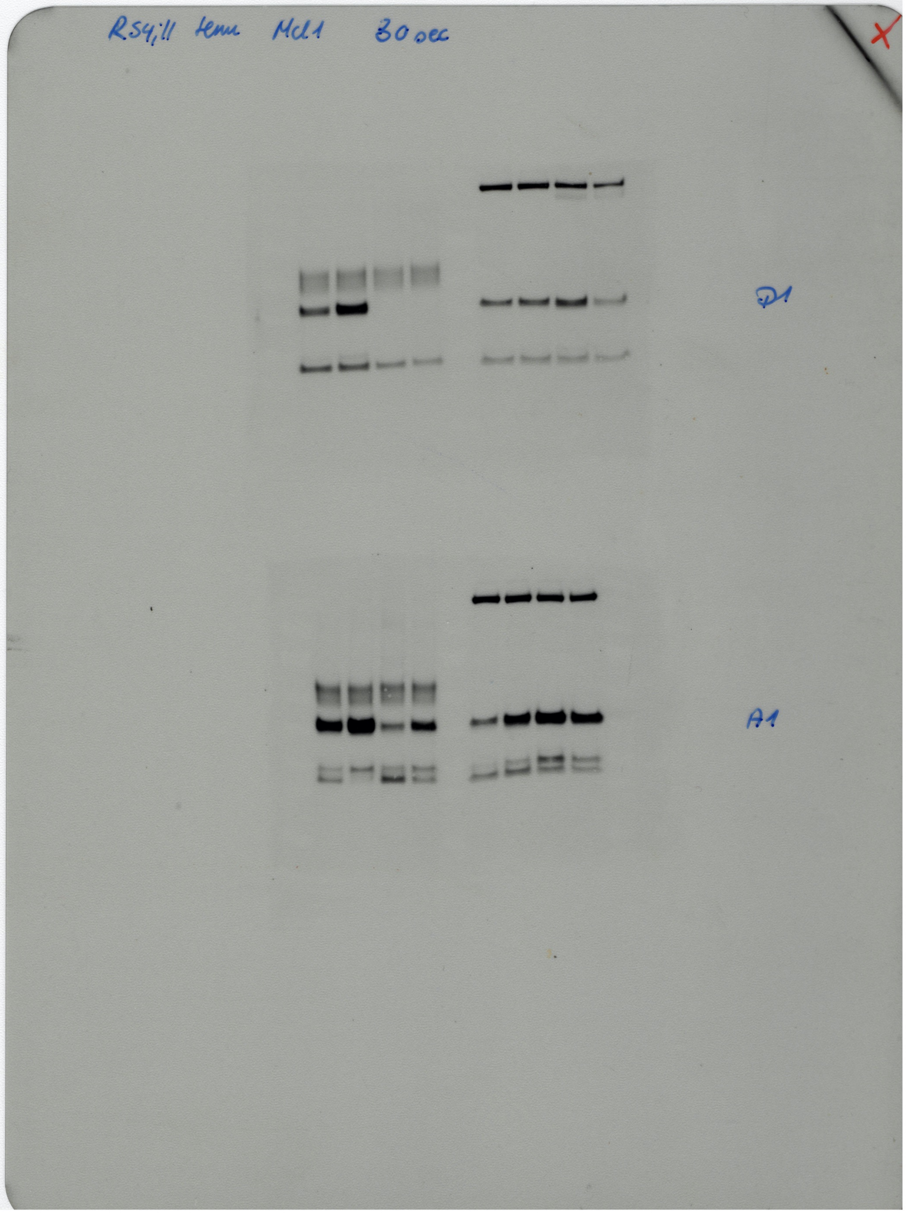
RS4;11
Venn
Mcl1
30 sec
D1
A1

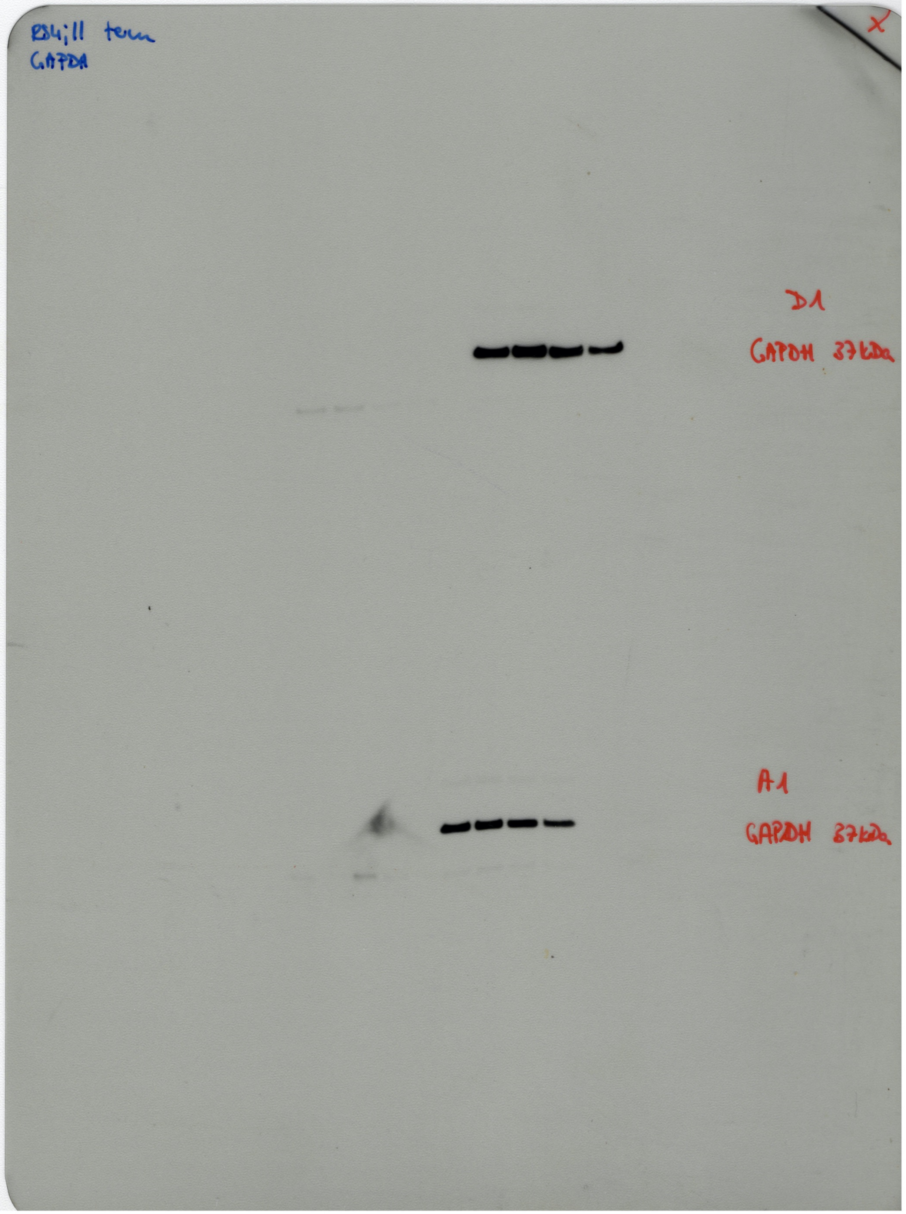
BS4;ll term
GAPDH
D1
GAPDH 37kDa
A1
GAPDH 37kDa

Figure 2F

RS4;11 term
Whole cell lysate

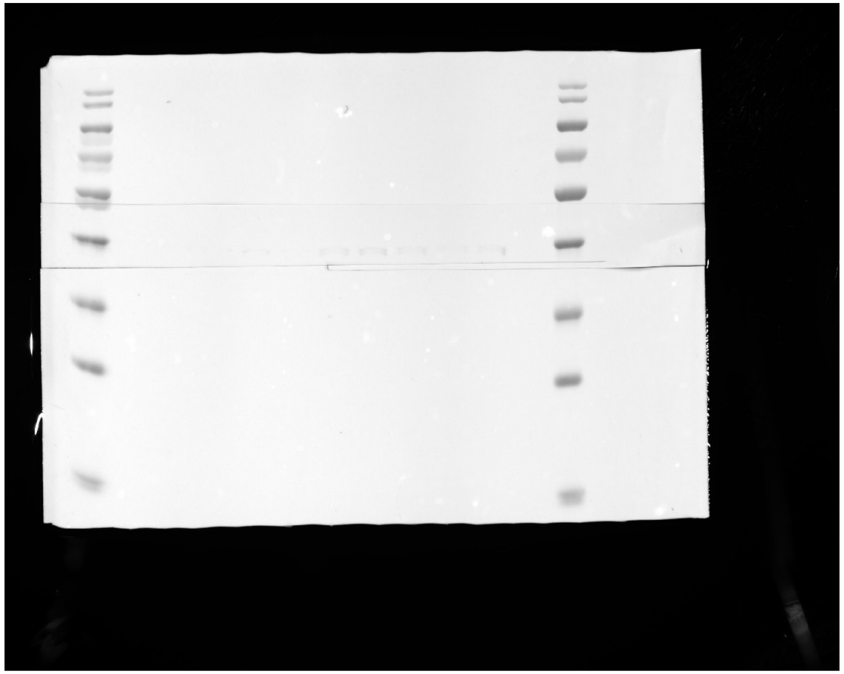

colorimetric

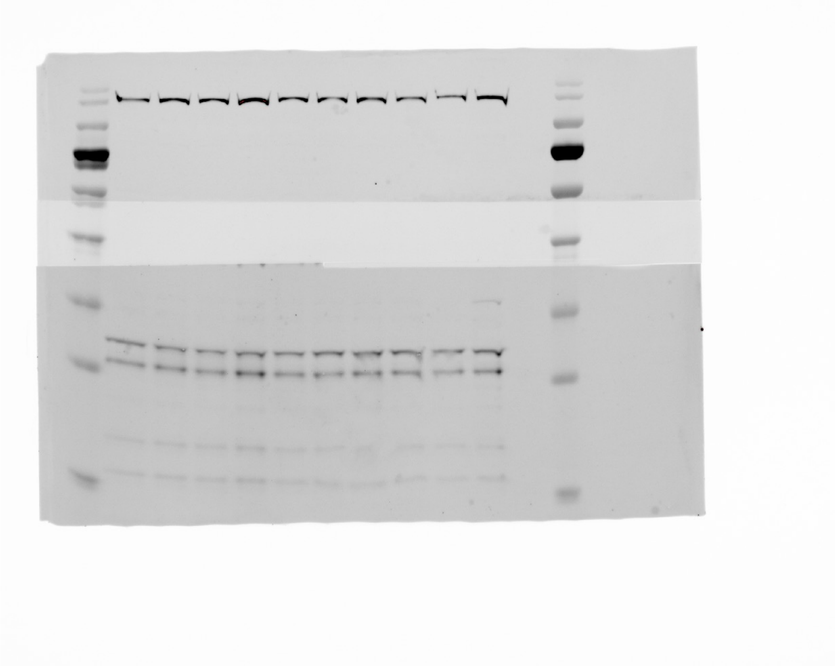

StarBright 700

RS4;11 term
Whole cell lysate

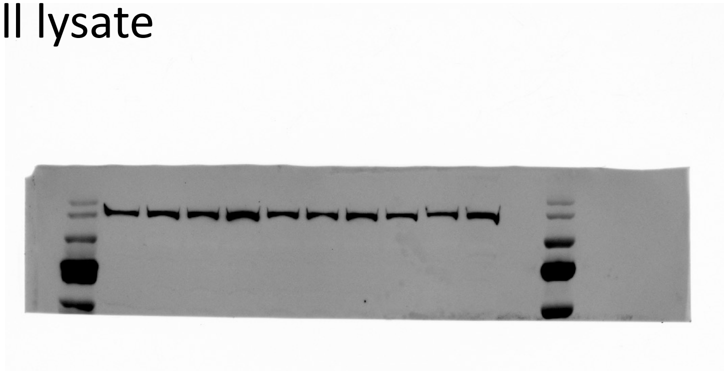

Vinculin

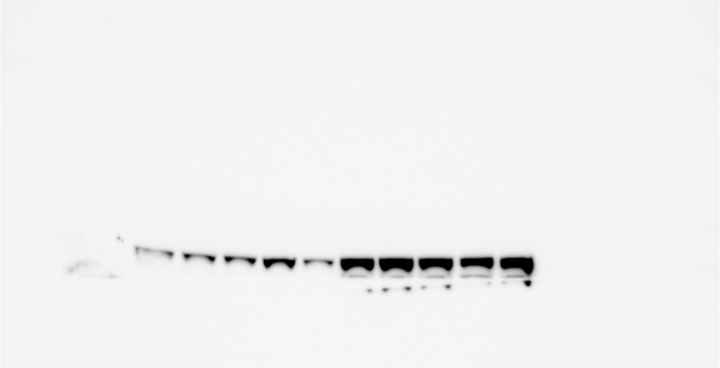

MCL-1

RS4;11 DH
Whole cell lysate

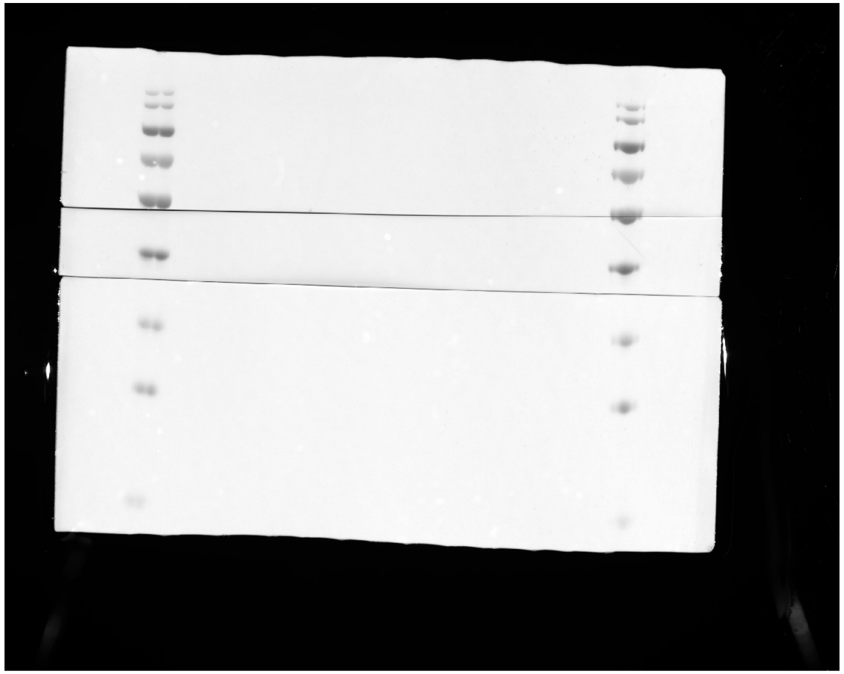

colorimetric

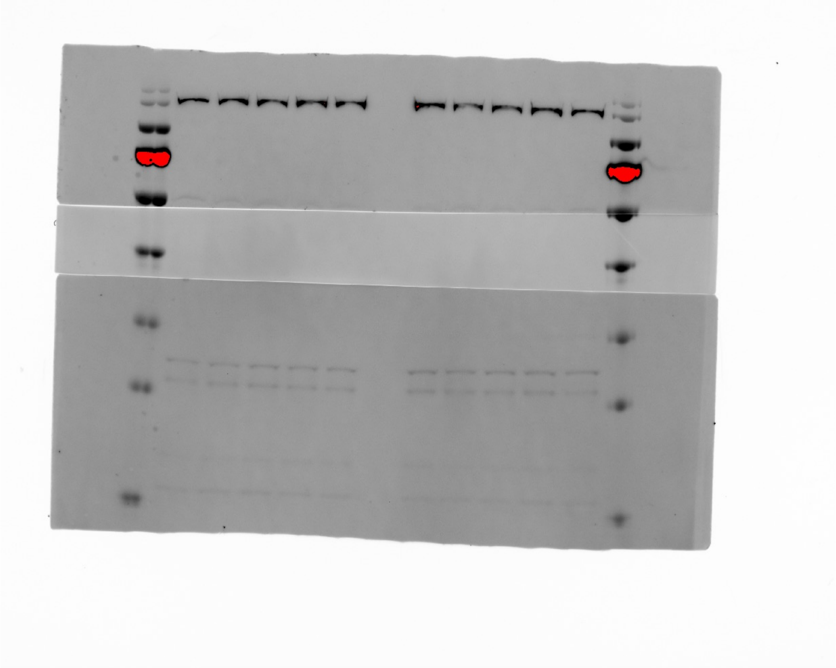

StarBright 700

RS4;11 DH
Whole cell lysate

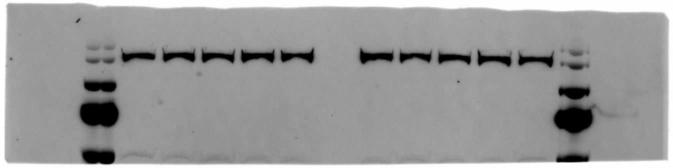

Vinculin

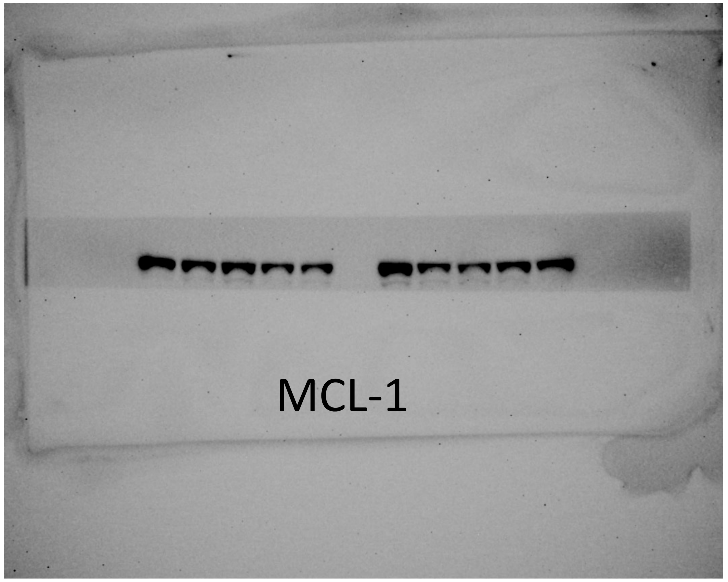

MCL-1

Figure 2l

RS4;11 term
BIM IP

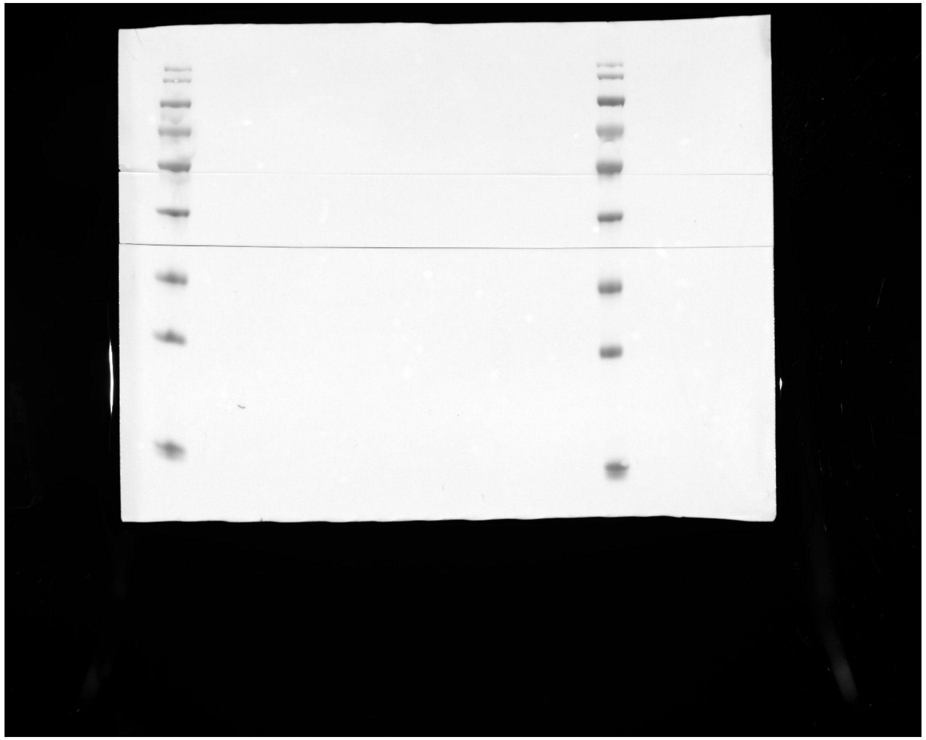

colorimetric

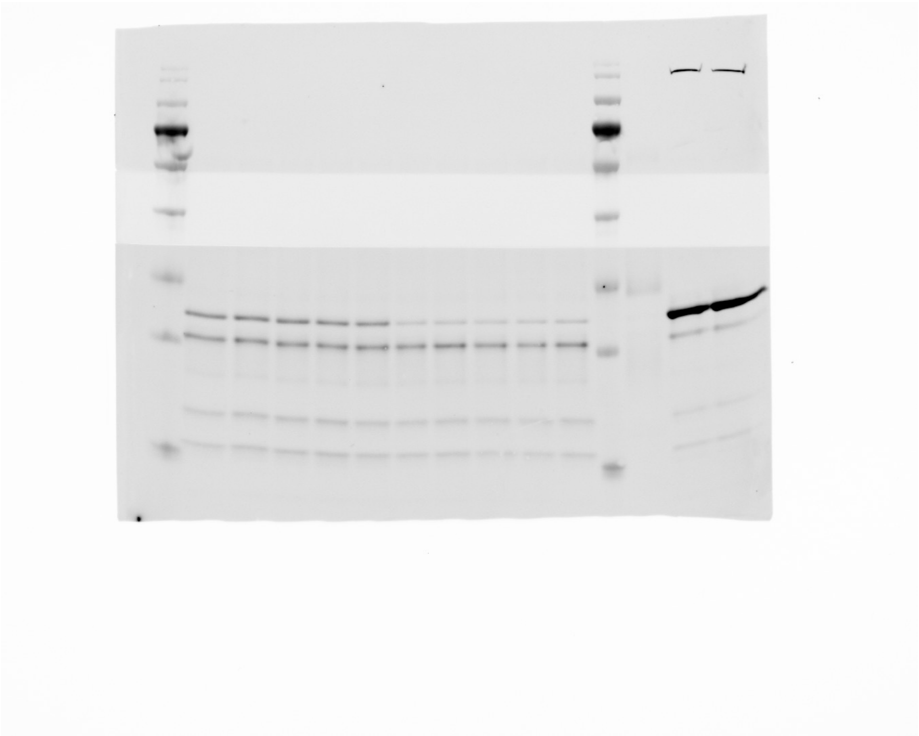

StarBright 700

RS4;11 term
BIM IP

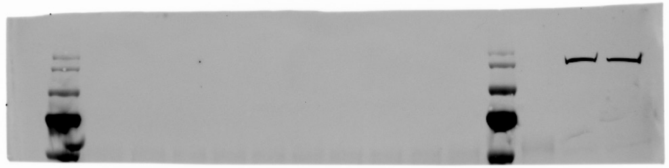

Vinculin

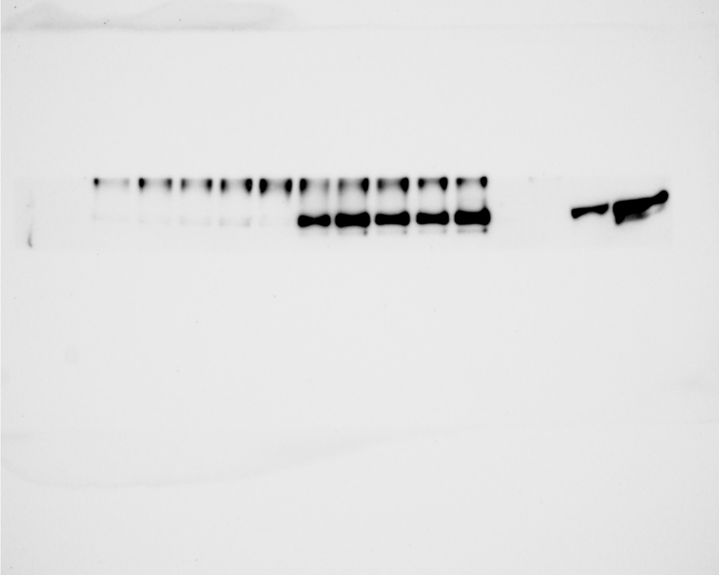

MCL-1

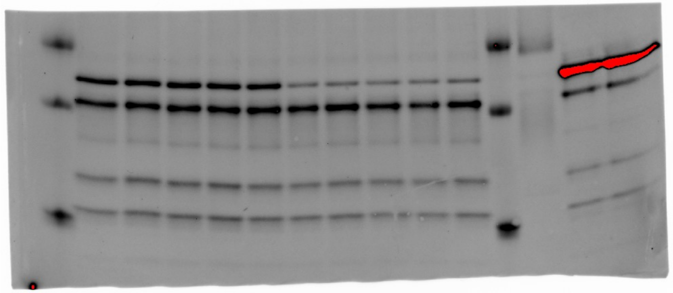

BIM

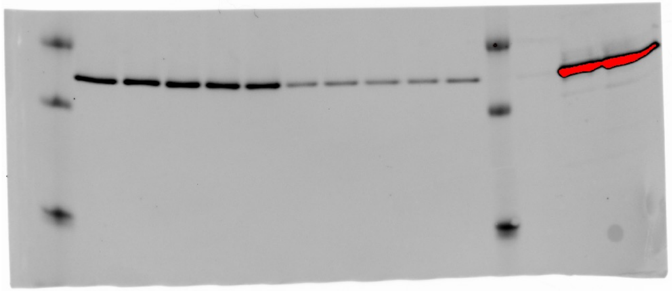

BCL-2

RS4;11 term
Whole cell lysate

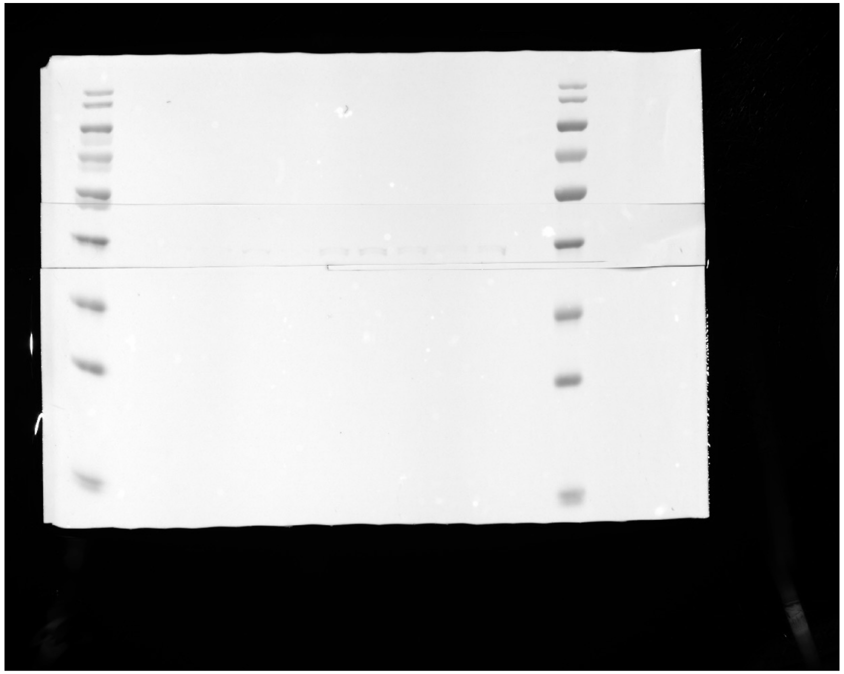

colorimetric

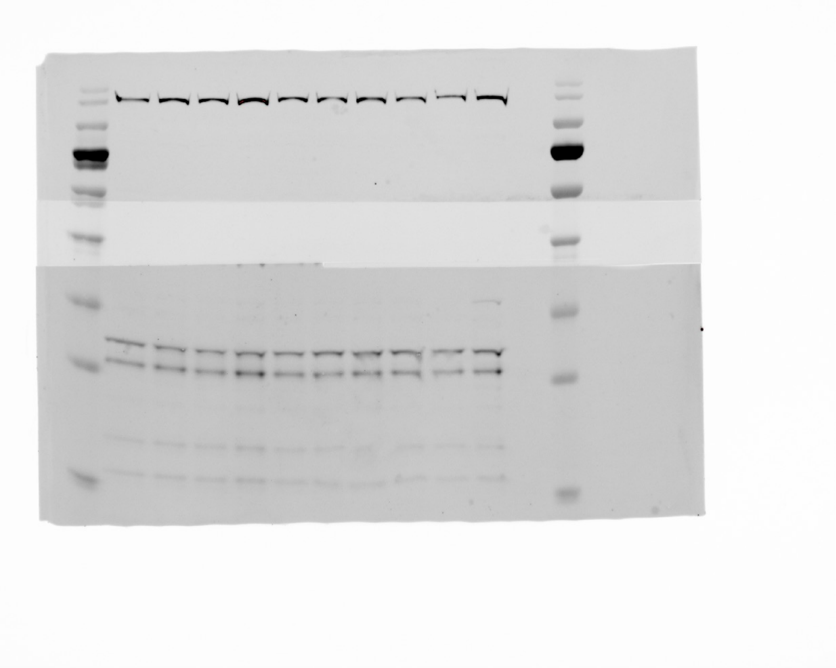

StarBright 700

RS4;11 term
Whole cell lysate

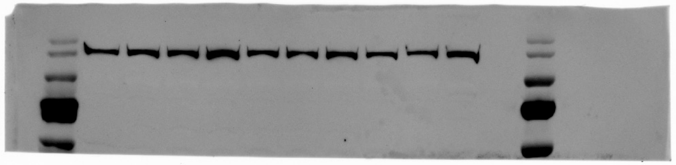

Vinculin

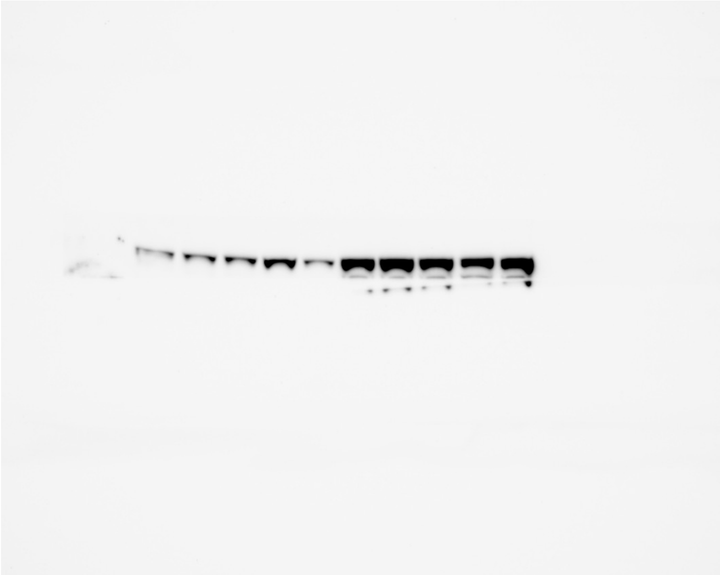

MCL-1

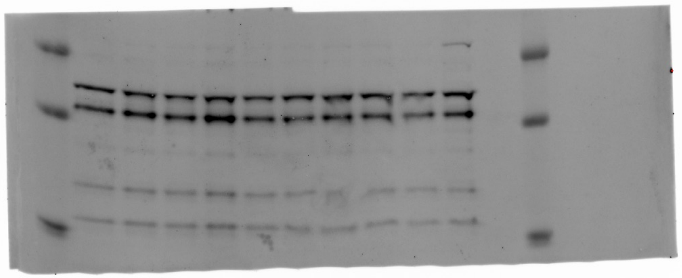

BIM

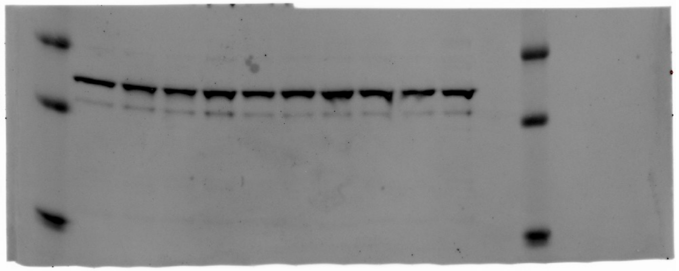

BCL-2

RS4;11 drug holiday
BIM IP

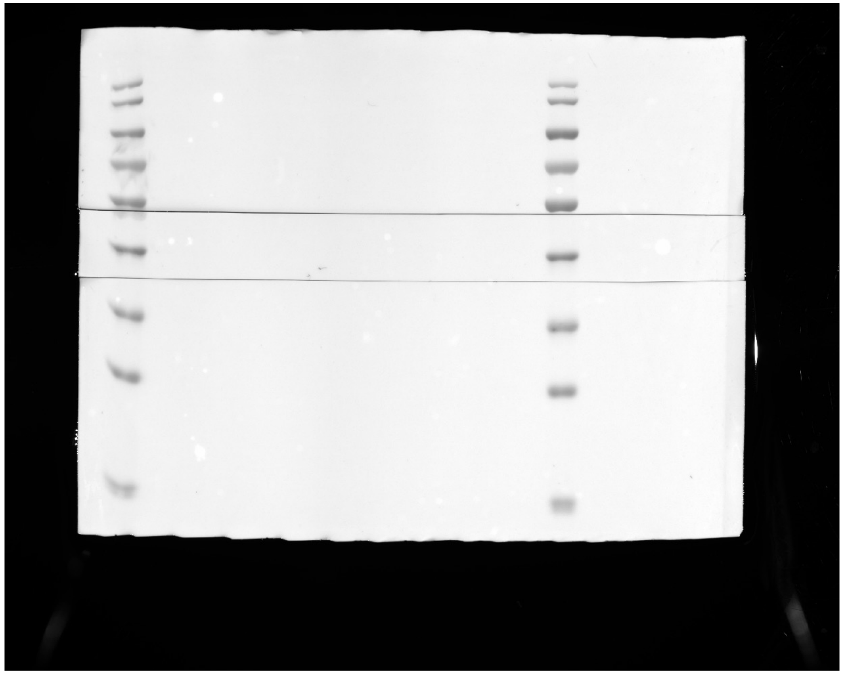

colorimetric

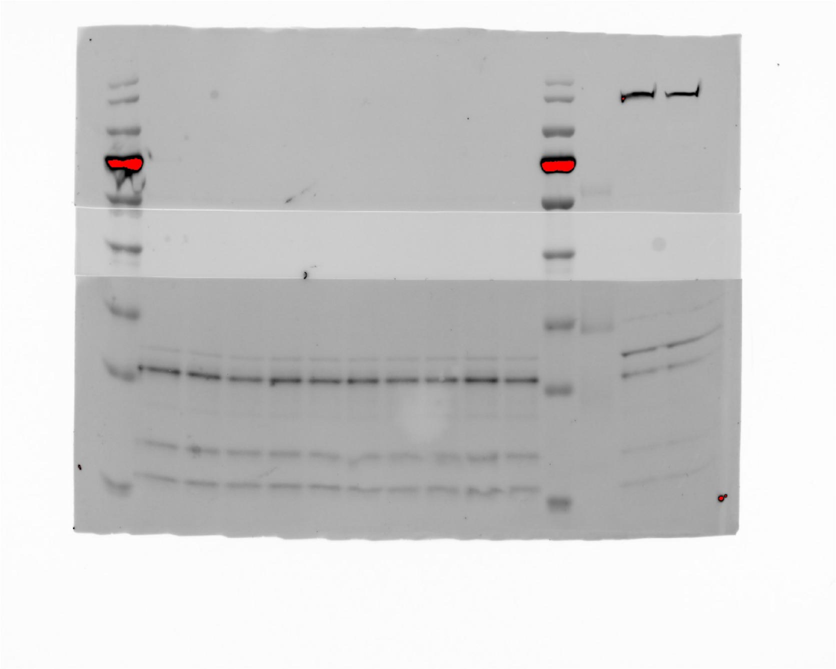

StarBright 700

RS4;11 drug holiday
BIM IP

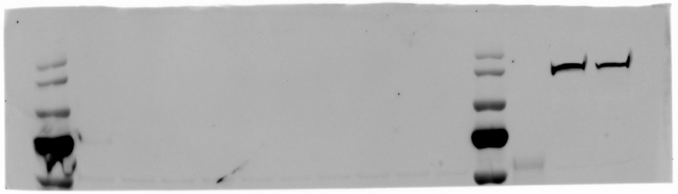

Vinculin

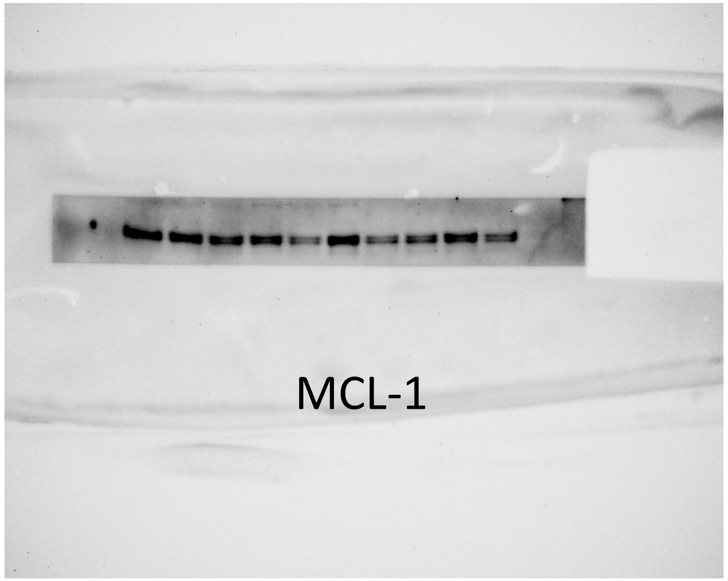

MCL-1

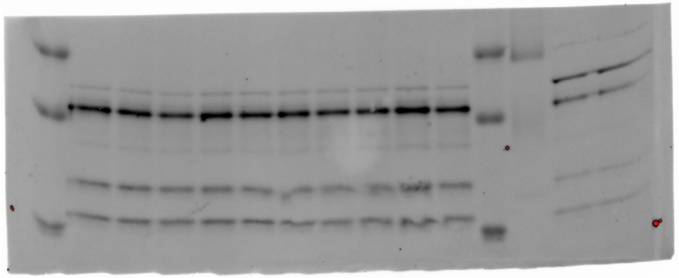

BIM

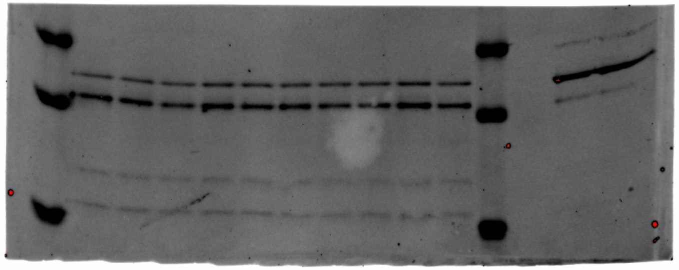

BCL-2

RS4;11 drug holiday
BIM IP

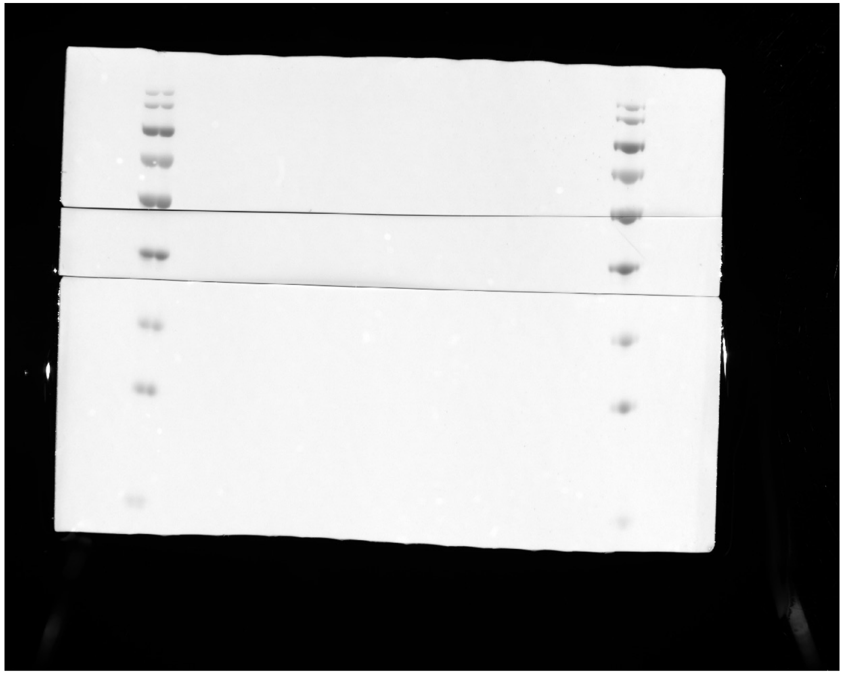

colorimetric

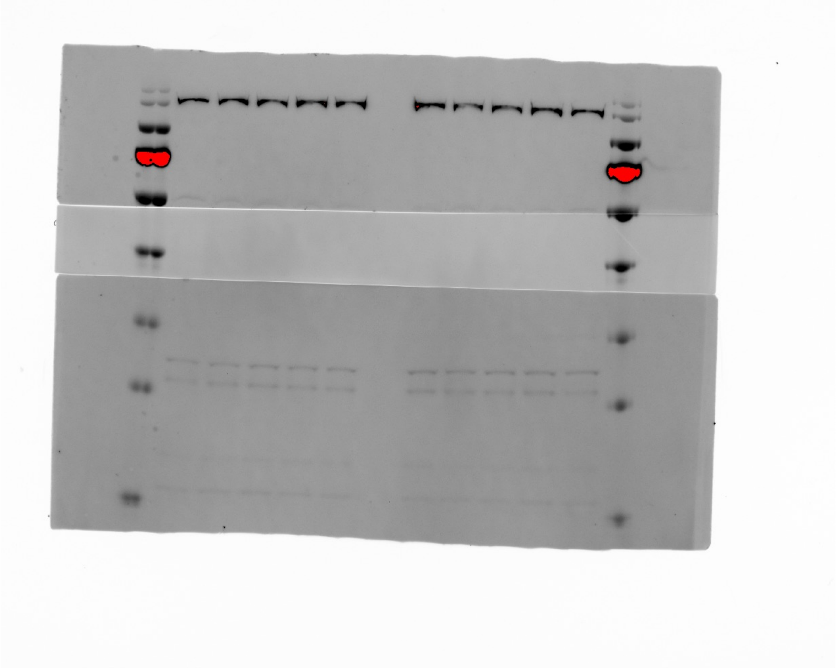

StarBright 700

RS4;11 drug holiday
Whole cell lysate

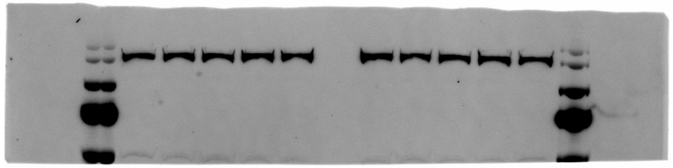

Vinculin

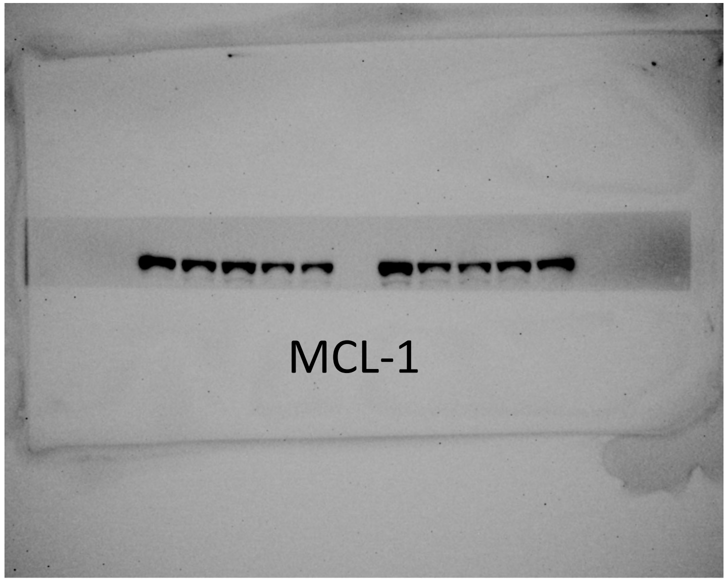

MCL-1

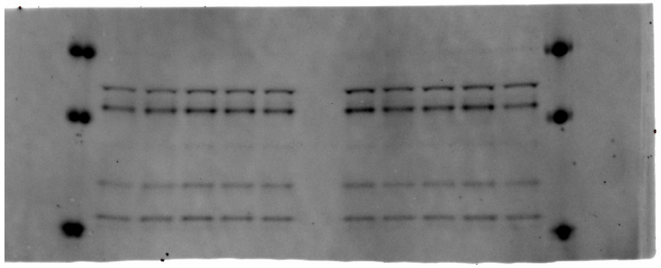

BIM

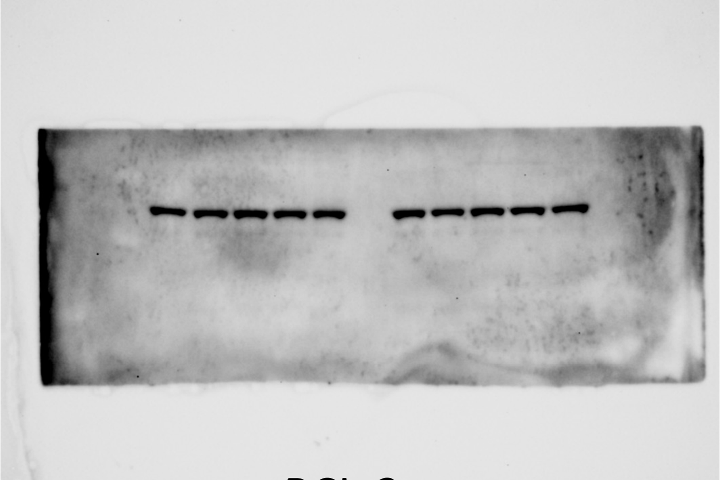

BCL-2

Figure 5D

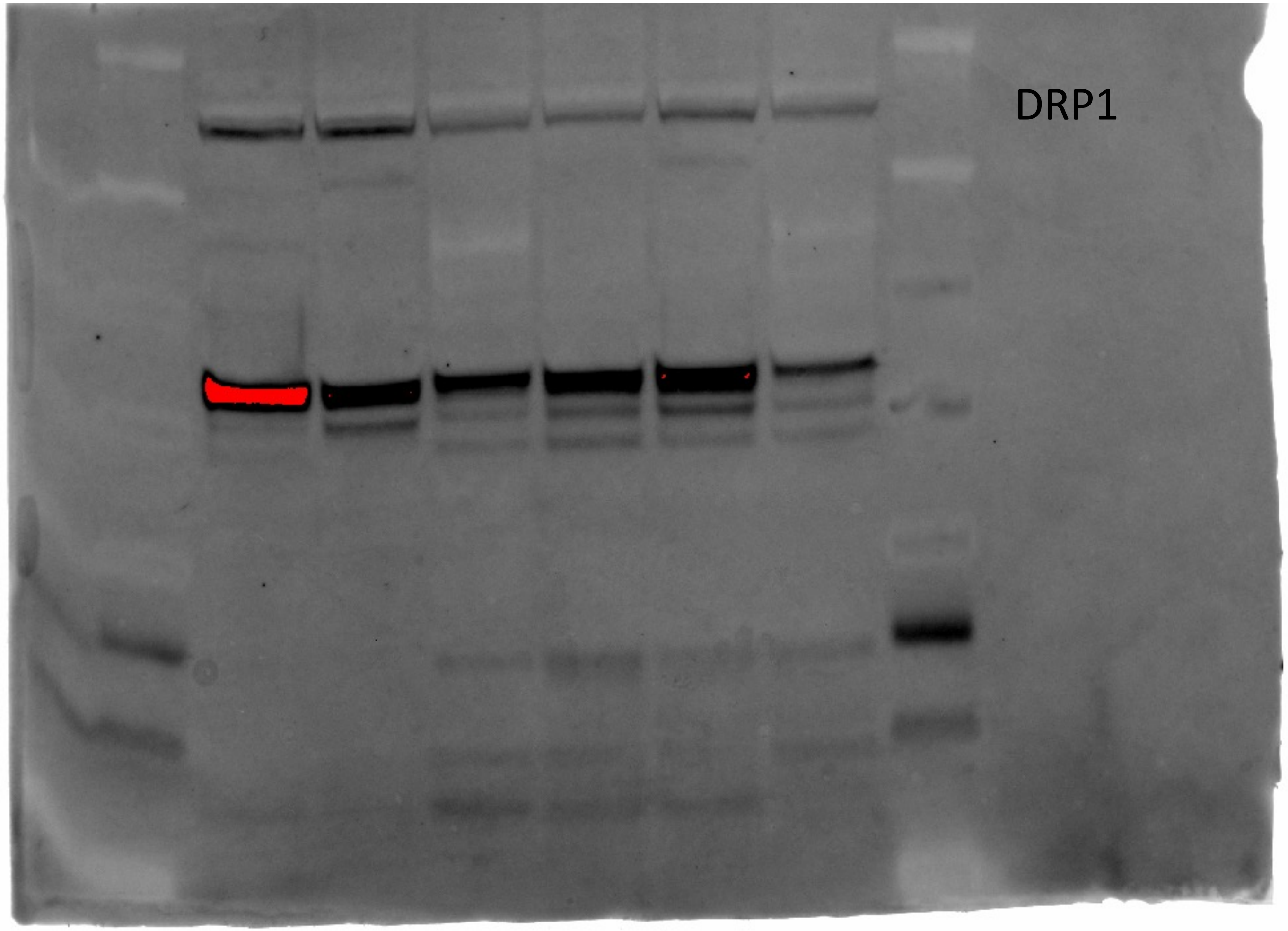
DRP1

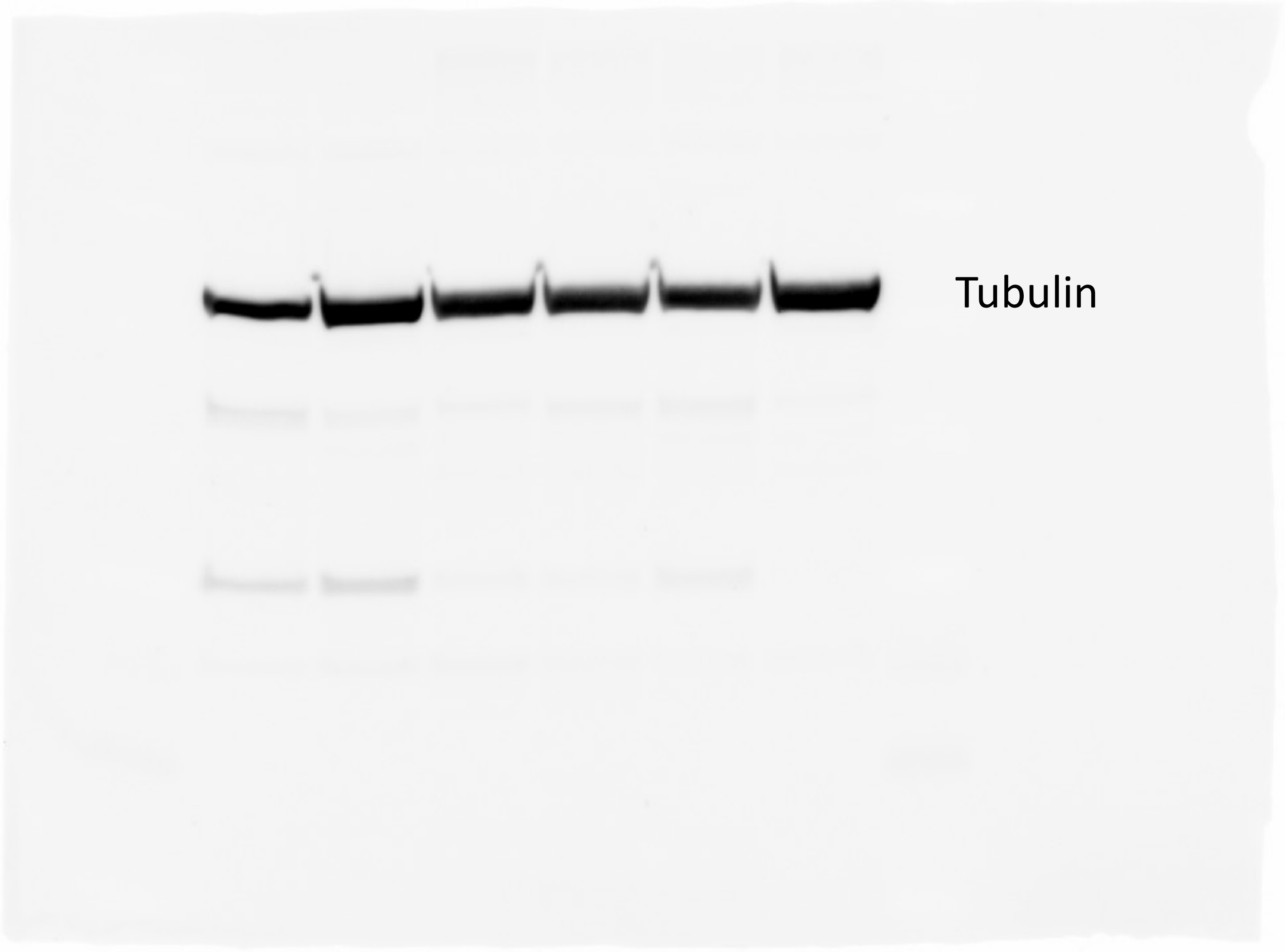
Tubulin

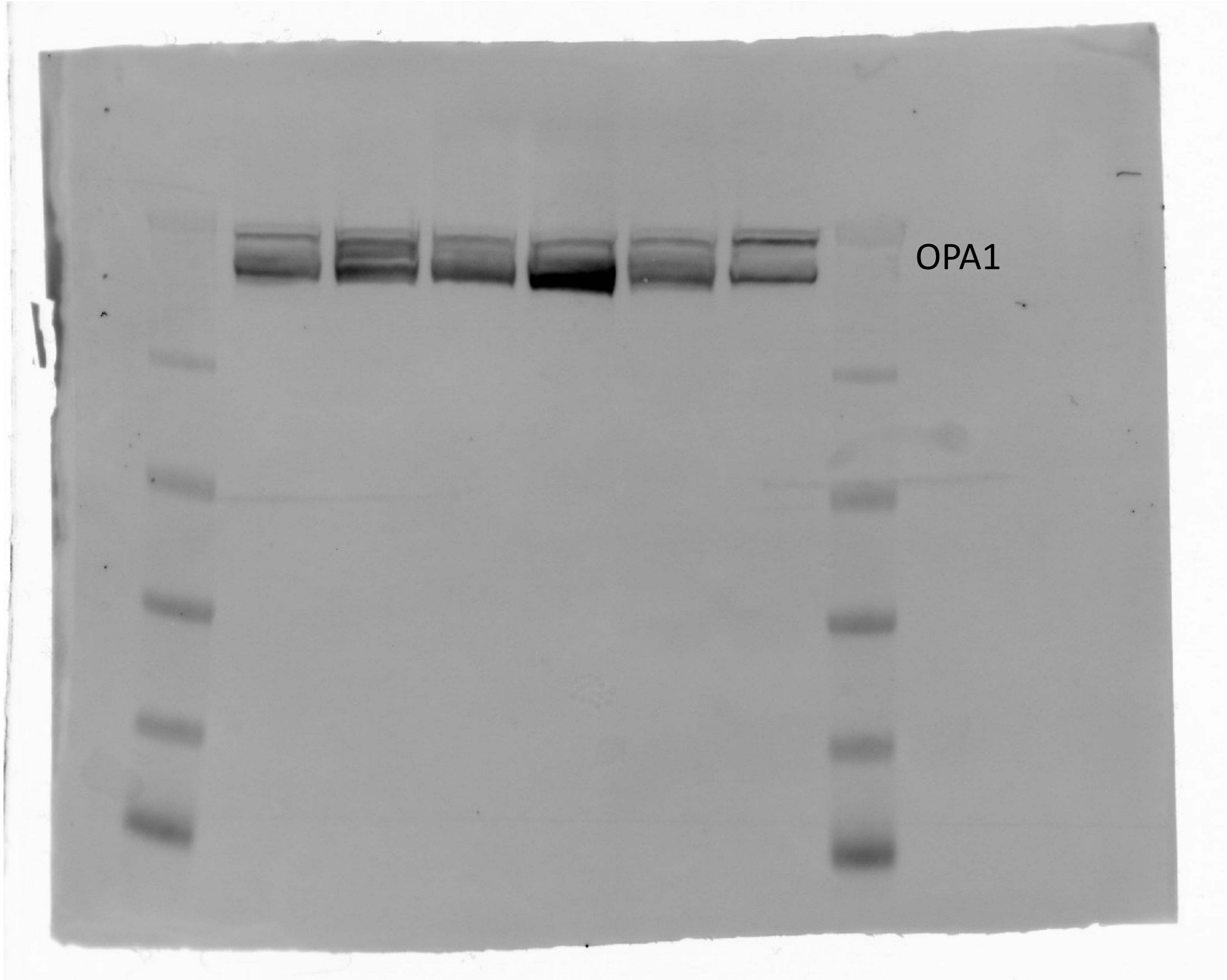
OPA1

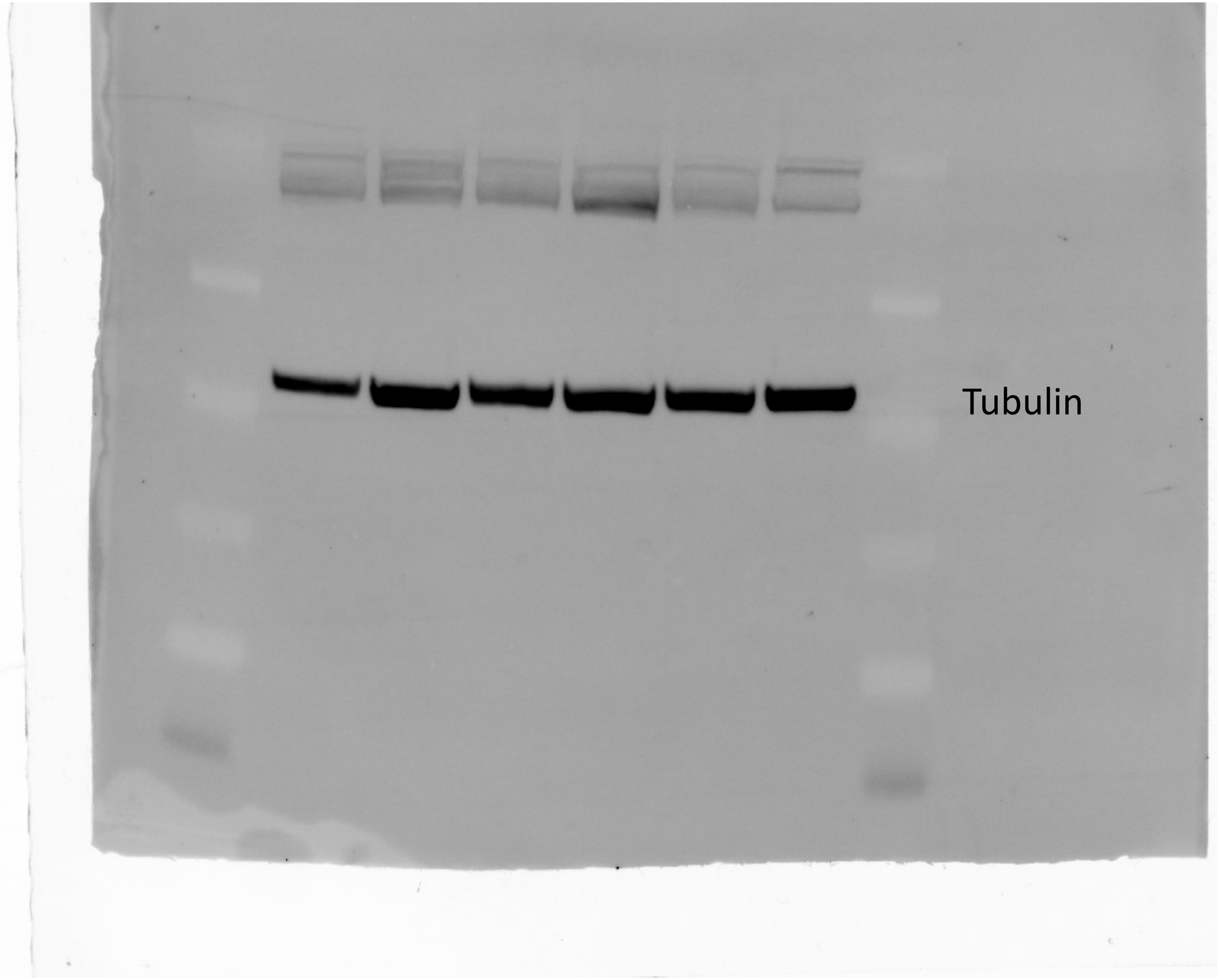
Tubulin

Supplementary Figure 2

Cell lines #1 + #2

BAK

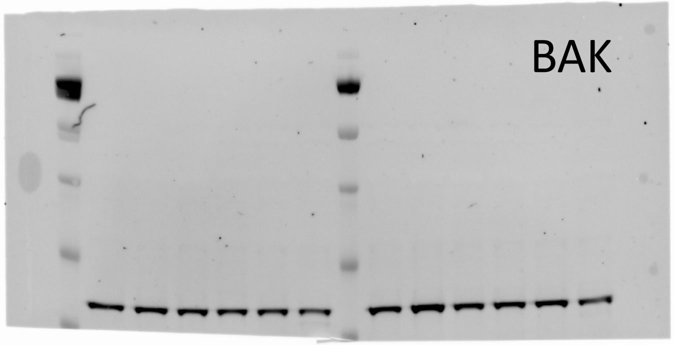

BAX

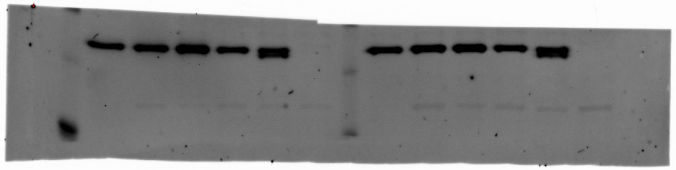

GAPDH

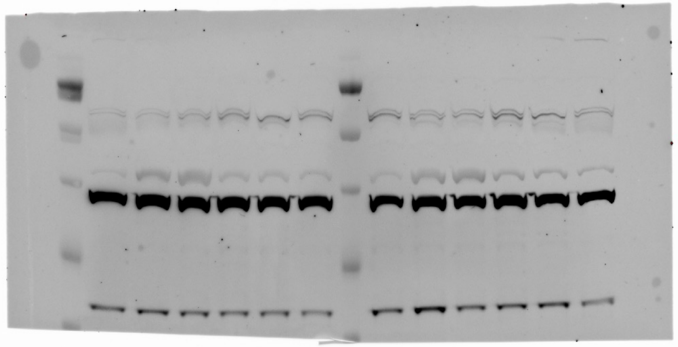

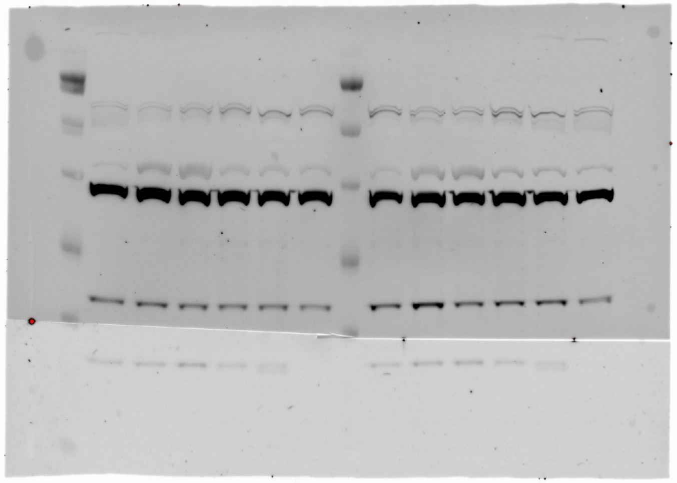

Complete blot

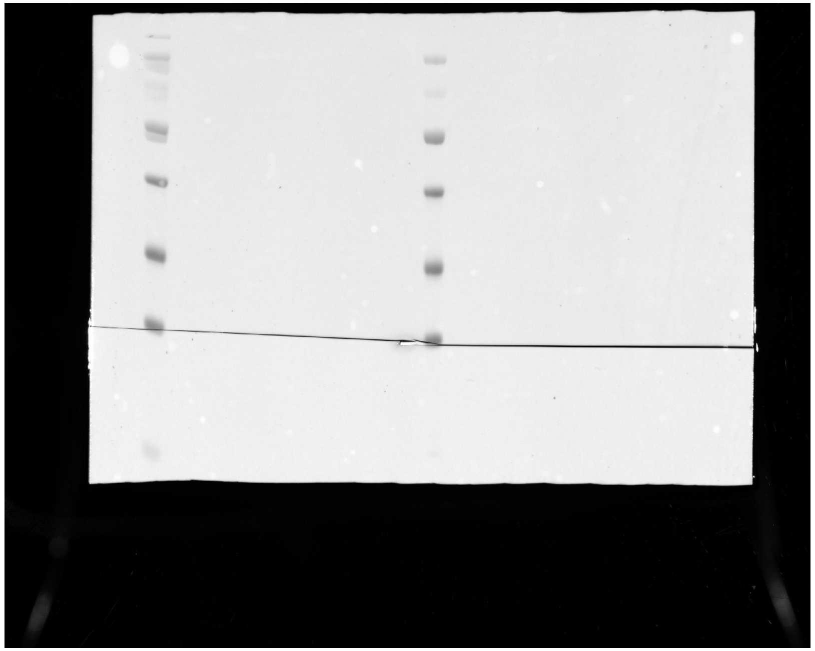

Cell lines #3

BAK

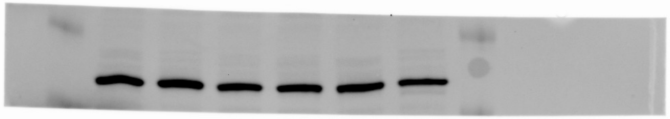

BAX

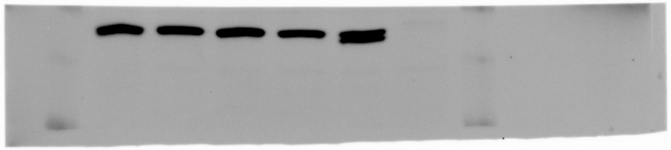

GAPDH

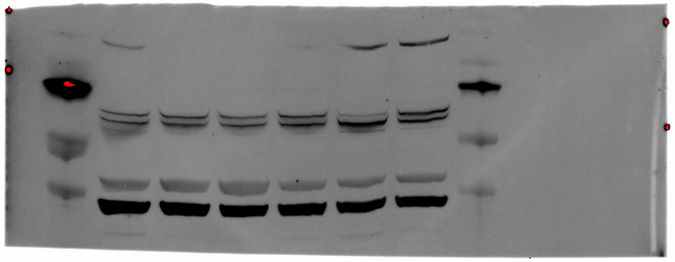

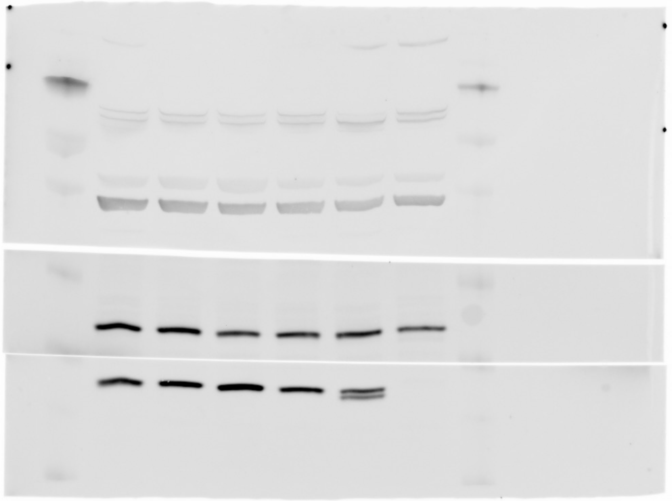

Complete blot

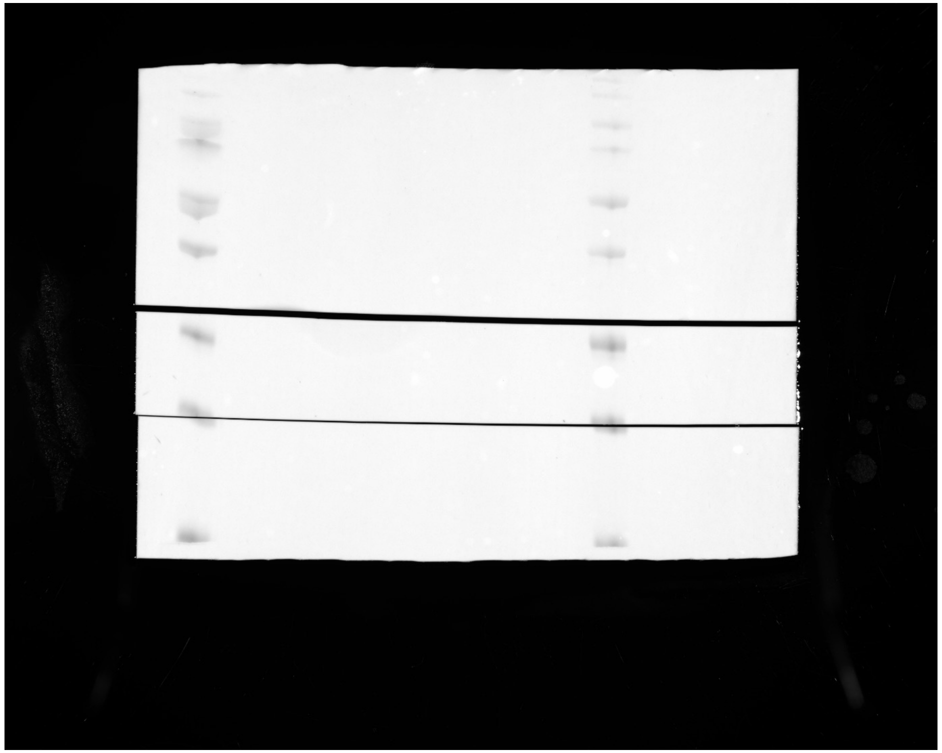

VENsens and VENinsens RS4;11

BAK

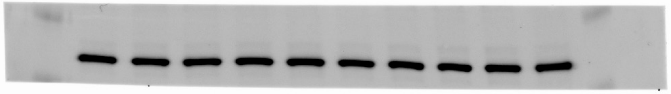

BAX

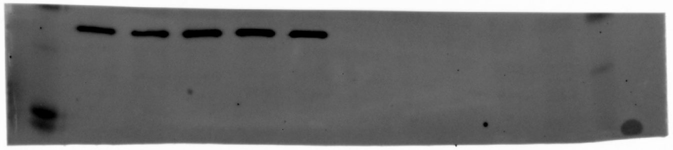

GAPDH

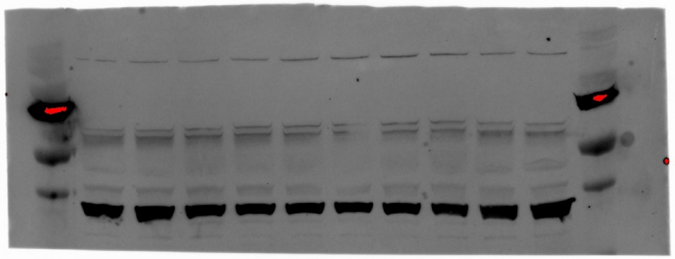

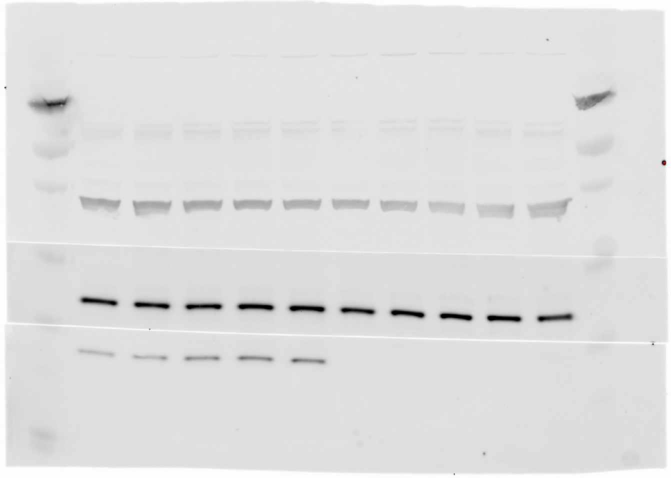

Complete blot

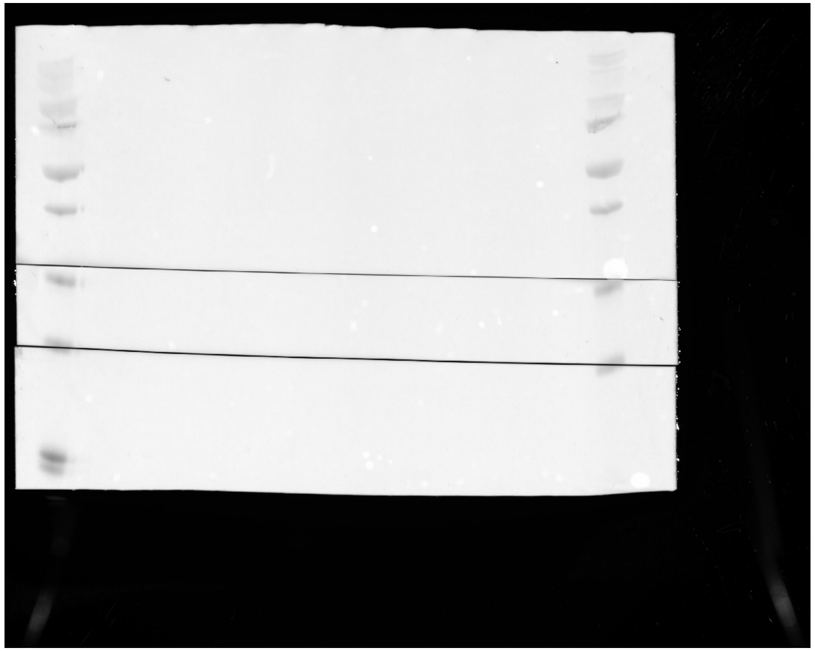

VENsens and VENinsens PDX

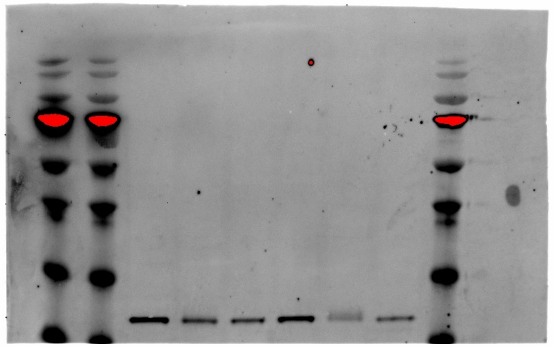

BAK

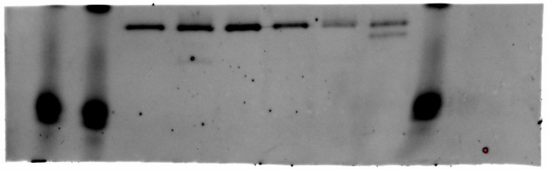

BAX

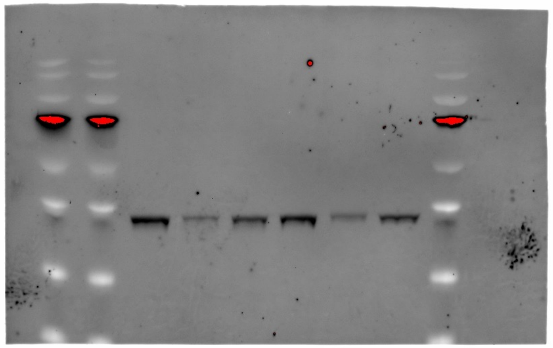

GAPDH

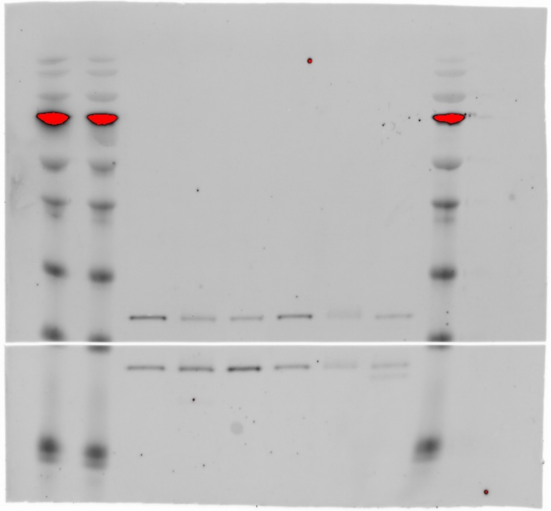

Complete blot

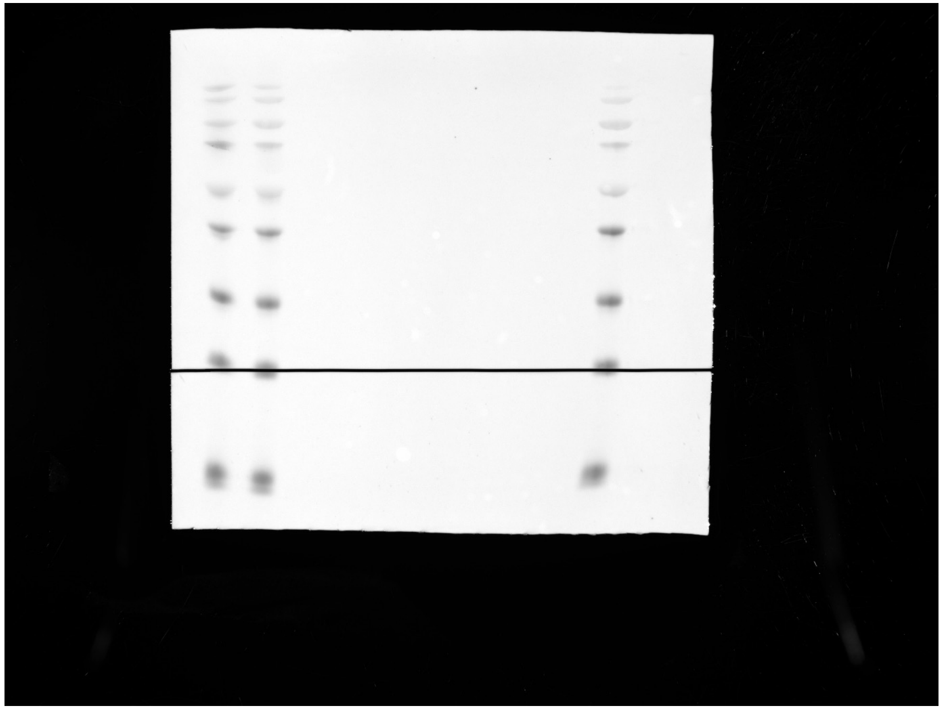

Supplementary Figure 3

RS4;11 VEN^{ins} 72h VEN vs 96h dh

MCL-1

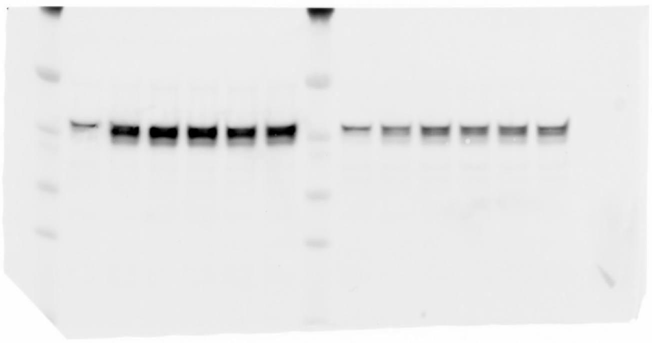

actin

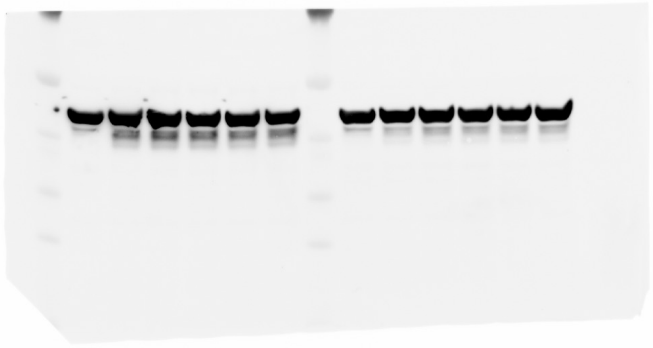

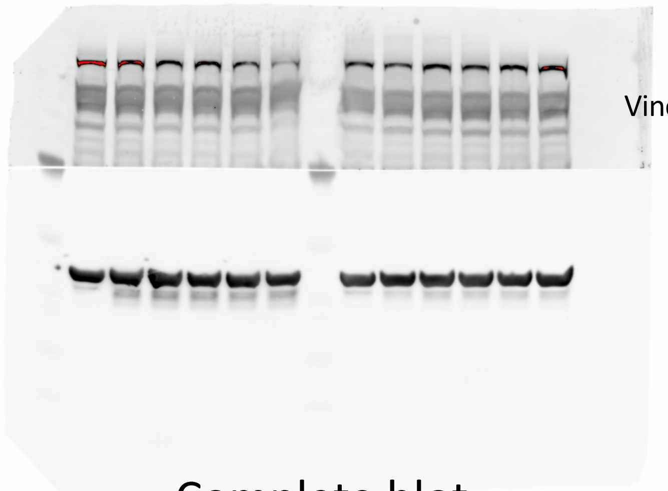

Vinculin did not work

Complete blot

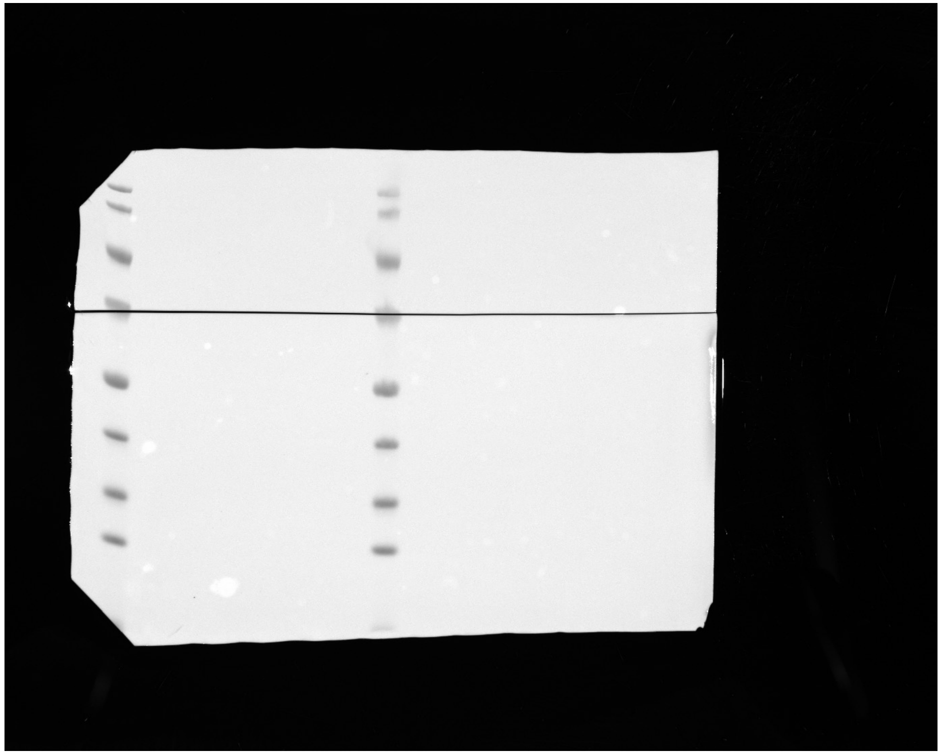

RS4;11 VEN^{sens} line 1 vs VEN^{ins} line 1

Time course

MCL-1

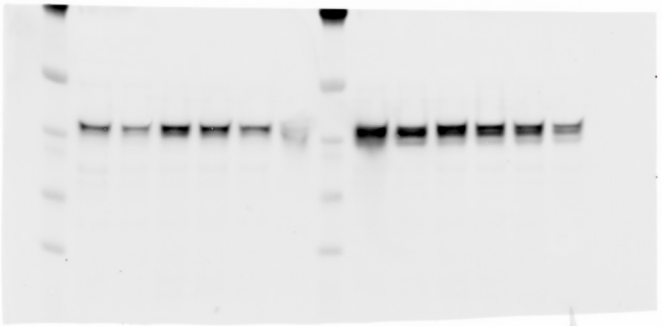

actin

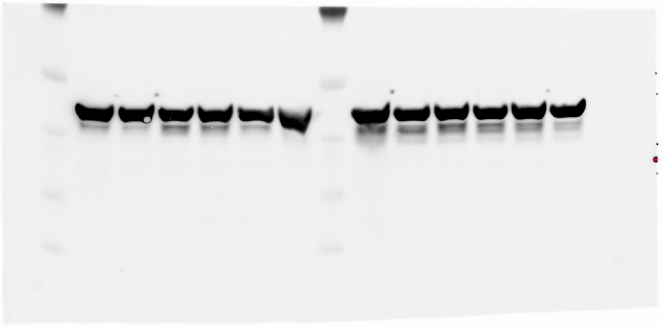

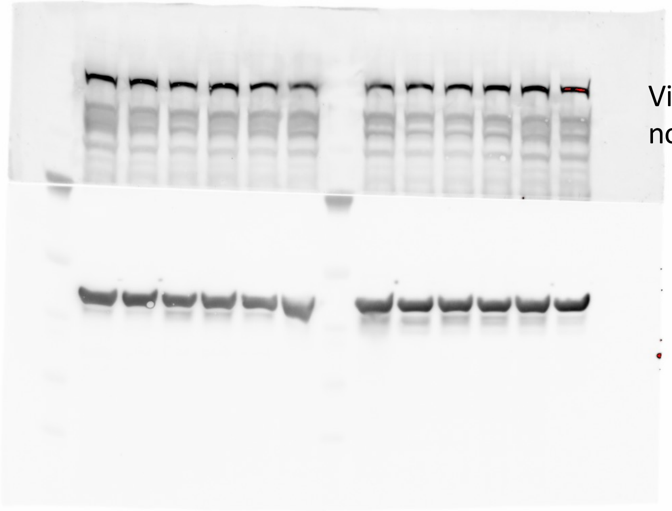

Vinculin did not work

Complete blot

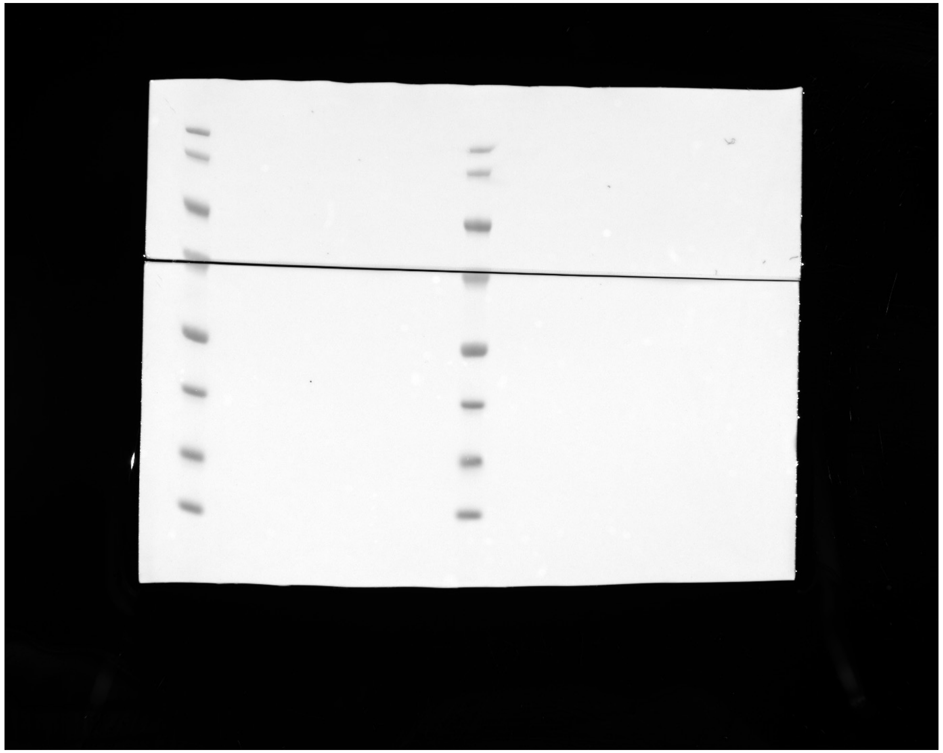

Supplement: Supplementary file 3 — Original Data File [file 41419_2024_6864_MOESM3_ESM.pdf]
